# Updated systematic review: associations between proximity to animal feeding operations and health of individuals in nearby communities

**DOI:** 10.1186/s13643-017-0465-z

**Published:** 2017-04-18

**Authors:** Annette M. O’Connor, Brent W. Auvermann, Rungano S. Dzikamunhenga, Julie M. Glanville, Julian P. T. Higgins, Shelley P. Kirychuk, Jan M. Sargeant, Sarah C. Totton, Hannah Wood, Susanna G. Von Essen

**Affiliations:** 1 Department of Veterinary Diagnostic and Production Animal Medicine, College of Veterinary Medicine, Iowa State University, Ames, IA USA; 2 Department of Biological and Agricultural Engineering, Texas A&M University, Amarillo, TX USA; 3 York Health Economics Consortium, University of York, York, UK; 4 School of Social and Community Medicine, University of Bristol, Bristol, UK; 5 Department of Medicine, Canadian Centre for Health and Safety in Agriculture, University of Saskatchewan, Saskatoon, Saskatchewan Canada; 6 Department of Population Medicine and Centre for Public Health and Zoonoses, University of Guelph, Guelph, Ontario Canada; 7 63 College Avenue West, Guelph, Ontario N1G 1S1 Canada; 8 Department of Environmental, Agricultural and Occupational Health, College of Public Health, University of Nebraska Medical Center, Omaha, NE USA

## Abstract

**Objective:**

The objective of this review was to update a systematic review of associations between living near an animal feeding operation (AFO) and human health.

**Methods:**

The MEDLINE® and MEDLINE® In-Process, Centre for Agricultural Biosciences Abstracts, and Science Citation Index databases were searched. Reference lists of included articles were hand-searched. Eligible studies reported exposure to an AFO and an individual-level human health outcome. Two reviewers performed study selection and data extraction.

**Results:**

The search returned 3702 citations. Sixteen articles consisting of 10 study populations were included in the analysis. The health outcomes were lower and upper respiratory tracts, MRSA, other infectious disease, neurological, psychological, dermatological, otologic, ocular, gastrointestinal, stress and mood, and other non-infectious health outcomes. Most studies were observational and used prevalence measures of outcome. An association between Q fever risk and proximity to goat production was reported. Other associations were unclear. Risk of bias was serious or critical for most exposure-outcome associations. Multiplicity (i.e., a large number of potentially correlated outcomes and exposures assessed on the same study subjects) was common in the evidence base.

**Conclusions:**

Few studies reported an association between surrogate clinical outcomes and AFO proximity for respiratory tract-related outcomes. There were no consistent dose-response relationships between surrogate clinical outcome and AFO proximity. A new finding was that Q fever in goats is likely associated with an increased Q fever risk in community members. The review results for the non-respiratory health outcomes were inconclusive because only a small number of studies were available or the between-study results were inconsistent.

**Systematic review registration:**

PROSPERO CRD42014010521

**Electronic supplementary material:**

The online version of this article (doi:10.1186/s13643-017-0465-z) contains supplementary material, which is available to authorized users.

## Introduction

Large-scale animal feeding operations (AFOs) are common in modern food production. Capacity varies, but it is not uncommon for facilities to house 1000 swine with multiple barns at a single site, feedlots to house 50,000 cattle, and poultry houses to house 250,000 hens. Communities near livestock facilities are exposed to odors and emissions. Study results suggest that livestock facilities that confine animals indoors for feeding represent a health hazard for surrounding communities. Numerous narrative reviews and opinion papers summarize the effects of these odors and emissions on community health. Our systematic review published in 2010 summarizes the community health effects of exposure to livestock production facilities [1]. That review employed many features of systematic review methodology, including assessment of risk of bias and extraction of magnitudes of effect. It revealed that many included studies used a hypothesis generation approach. Most studies on the topic use prevalence measures of disease, and it was difficult to reach strong conclusions about associations. The final conclusion from the original review was that “Few studies reported an association between surrogate clinical outcomes and AFO proximity. A negative association was reported when odor was the measure of exposure to AFOs and self-reported disease, the measure of outcome. There was evidence of an association between self-reported disease and proximity to AFO in individuals annoyed by AFO odor. There was inconsistent evidence of a weak association between self-reported disease in people with allergies or familial history of allergies. No consistent dose-response relationship between exposure and disease was observable.”

### Review objectives

Studies relevant to the original review question have been published since 2010. Therefore, the objective of this review was to update the 2010 review. The research question was: “What are the associations between animal feeding operations and measures of the health of individuals living near animal feeding operations, but not actively engaged in livestock production?” “Animal feeding operations” were defined as enterprises used to rear animals for food production on any scale. “Health” was defined as a state of complete physical, mental, and social well-being and not merely the absence of disease or infirmity, as defined by the World Health Organization. “Actively engaged” was defined as owning or working on a livestock production facility. Except for the definition for “health,” these definitions were used in the 2010 review. In this review, we expanded “health” to include antimicrobial resistance patterns of organisms cultured from individuals living near animal feeding operations.

## Methods

### Protocol and registration

Descriptions of the intended search strategy, eligibility criteria, study selection, data collection process, assessment of risk of bias, and the approach used for synthesis of results were included in protocol documentation prepared in advance. The PROSPERO (http://www.crd.york.ac.uk/PROSPERO) protocol registration number is CRD42014010521. The protocol has also been published [2].

### Eligibility criteria

The population of interest was people living in communities near and not near animal feeding operations that might reasonably be described as “industrial” or “modern.” Studies that assessed the effects of occupational exposure to livestock were excluded from the analysis. A value for distance for “near” an operation was not defined because we expected that most of the studies would not include this information. Study authors either reported populations as exposed or not exposed based on an author-defined cut-off or used an exposure gradient, and the lowest level of exposure was no exposure. For the exposure, we did not limit the definition of a livestock production facility to that described by the Environmental Protection Agency’s definition of a confined animal feeding operation (CAFO) because we expected that many studies would not provide sufficient information on livestock population size. Grass-based, nomadic, and confined smallholder operation production systems were not relevant to the review. The protocol did not include the “hobby farm” as an excluded population, but we excluded this operation type from the review. Studies that reported any measure of exposure to animal feeding operations (e.g., odor severity, endotoxin levels in air, distance from AFOs, modeled exposures based on extrapolation from empirical data) were eligible for inclusion. There were no restrictions on the date of the study.

The eligible outcomes were measured in individuals in the exposed or unexposed categories, or who were compared using some measure of degree of exposure. Based on the previous review results, most of the outcomes were expected to be respiratory- or mental health-related. However, other outcomes were also eligible. We did not exclude studies based on the approach used for outcome measurement because measurement error was included in the risk of bias assessment. Outcomes that did not represent direct health measures in humans (e.g., antimicrobial resistance patterns in soil or water resources) were not eligible.

Eligible study designs included observational studies of any health outcome or resistance of resident (colonized) bacterial populations measured directly in human subjects. Animal models of human disease were not included because it was unclear how an animal model of human disease would accurately reproduce short- and long-term effects of exposure to AFOs. The confounding effects were reduced by excluding studies that included only one unit of measurement (i.e., one farm per exposure group). Ecological study designs (i.e., the unit of measurement of the exposure and the outcome is a population aggregate) were not eligible because this design did not allow a direct comparison between exposure and outcome in individuals. Reports were not excluded based on language or publication year. News, editorials, and letters were not included because these were unlikely to describe original research.

### Information sources

Electronic searches of the MEDLINE® and MEDLINE® In-Process (via OvidSP) (1946–2014), Centre for Agricultural Biosciences (CAB) Abstracts (via Web of Knowledge) (1910–2014), and Science Citation Index (via Web of Knowledge) (1900–2014) databases were performed. The search strategy was adapted for each resource, while accounting for differences in search syntax and indexing. The last search was 3 October 2014.

In addition to searching bibliographical databases, the included articles’ reference lists were searched by hand to identify articles that were not identified by the electronic search. Citation searches of relevant studies were not performed. The protocol specified that when a search identified evidence published in non-peer-reviewed sources (e.g., theses or conference proceedings), searches of MEDLINE®, CAB Abstracts, and the Science Citation Index would be performed of the first author’s name to retrieve associated publications. However, all of the studies included in the review were from peer-reviewed sources and follow-up searches were unnecessary.

### Search

The search strategy used to identify articles on animal feeding operations and community health in Ovid MEDLINE® and MEDLINE® In-Process is presented in Additional file 1: Table S1. The strategy comprised two concepts (i.e., animal feeding operations (search lines 1–10) and community health (search lines 11–19)). Animal studies were removed in line 21. This search line excluded studies that only reported animal health outcomes.

Search strategy sensitivity was tested against the studies included in the prior version of the review. The search identified all of the studies indexed in MEDLINE®. This result suggested that the search had acceptable sensitivity. All search results were uploaded to EndNote bibliographic management software (Thomson Reuters, Philadelphia, PA, USA) and were de-duplicated.

### Study selection

The search results were also uploaded into online systematic review software (DistillerSR®, Ottawa, ON, Canada). The primary reviewers were veterinarians with post-graduate training in epidemiology and experience in systematic review methodology. During Level 1 screening, the two independent reviewers used the following question to assess the relevance of citation abstracts and titles:

1. Does the title and/or abstract describe primary research reporting associations between livestock (intensive, not pastoral) and human interactions (direct or indirect) and measures of human health measured in humans?

Citations were excluded if both reviewers answered “no” to this question. Although titles and abstracts not written in English were not considered, non-English papers with English titles and abstracts were included in the Level 1 screening. At the first level of relevance screening, reviewers were aware of the journal and author name(s) (a deviation from the original protocol). Non-English language articles were translated into English. The prior review identified two relevant German-language publications; a translation of each article was available.

Each citation that passed Level 1 screening progressed to Level 2. Two independent reviewers then assessed the full text of each article based on the above question. Each reviewer then used the following questions to assess each article retained after the full-text evaluation:

1. Does the study use a unit of analysis at the individual human level in the community (but not occupational, such as farm worker)?
2. Does the study include more than one unit of measurement of exposure?

The study was excluded if both reviewers answered “no” to either question. If both reviewers answered “yes” to both questions, the study progressed to the data extraction. At all stages of screening, disagreements between reviewers were resolved by consensus or, if necessary, by including a third reviewer and accepting the majority decision.

### Data collection process

Before the abstract and full-text screenings, the reviewers responsible for data extraction received training to ensure consistency. Data extraction was performed by at least two independent reviewers using a pretested form for the study- and outcome-level information (available at Iowa State University (ISU) Digital Depository (DD) URL). Conflicts were resolved by consensus or, when necessary, by the judgment of a third reviewer. Investigators were not contacted to confirm or obtain missing or unpublished data. Multiple reports of a single study were identified by one or more of the following characteristics: the study location, study name, sample size, study methodology, and authors’ names. All reports of a given study were considered during the data extraction and risk of bias assessment to obtain the most complete dataset. During data verification, each co-author was provided with the extracted data from a subset of papers and asked to verify its accuracy.

### Data items

For each study, reviewers extracted the study year, the time period, the study population’s location (country and region within the country), the size of the (human) source population, the size, age, sex, and socioeconomic status of the (human) study population, the size of the animal population in the source population, the metric(s) and units used to define the measured animal-related variable(s) (e.g., distance from the facility, odor, endotoxin levels), a description of the human community (e.g., “neighboring residents of animal farms in the Dutch provinces of Noord-Brabant and Limburg”), and the statistical approach the investigators used to analyze the data.

For the reported outcome measures, we extracted the assessed outcome type, the community exposure/animal variables, and the effect size estimate comparing exposed and unexposed people (e.g., a regression coefficient or a function of a regression coefficient, such as an odds ratio (OR)). We extracted the measure of precision of the effect estimate for all outcomes. In the protocol, we indicated that we would extract the number of people included in each category. However, this step was not performed because there were a large number of outcomes.

Experience from the previous review suggested that regression models were common and that models may be adjusted or unadjusted for known confounders. Therefore, we also extracted information about studied or assessed confounding variables and about confounders included in the final adjusted model. If the authors reported that an effect modifier was statistically significant and that the data were presented for each level of that effect modifier, then the data were extracted separately for each level of the effect modifier.

### Risk of bias in individual studies

Risk of bias was assessed at the study (if only one outcome) or outcome (if the study had multiple outcomes) level by two reviewers working independently using one of three pretested forms (i.e., a tool for non-randomized case-control and cross-sectional studies, a tool for non-randomized cohort studies, and a tool for non-randomized experimental studies (available at Additional file 1), as appropriate. The risk of bias in observational studies was assessed using the domains of confounding, selection, exposure measurement, missing data, outcome measurement, selection of reported result, and overall bias. The judgment outcome options were a low, moderate, serious, or critical risk of bias. The risk of bias in non-randomized experimental studies was assessed for the domains of selection, performance, detection, attrition, reporting, and other (i.e., any biases not included in the preceding five domains). The judgment outcome options were a low, high, or unclear risk of bias. A preliminary version of a new risk of bias assessment tool for non-randomized studies of interventions (ROBINS-I) was used for risk of bias evaluation for the observational studies [3]. The templates designed by [3] were modified after pilot-testing on two papers and were revised for the current review. All references to “interventions” in the ROBINS-I templates were changed to “exposures” because this term was more appropriate for the review question, which was about an exposure rather than an intervention. An added question asked reviewers to identify the objective versus subjective exposure/outcome combination being assessed.

Assessment of bias in the non-randomized experimental studies was performed using a modified Cochrane Risk of Bias Tool [4] (i.e., for the observational studies, a question was added to identify the subjective versus objective exposure/outcome category being assessed). We used the following guide for the question, “What was the risk of bias due to allocation method?”: If the authors did not describe the method used to randomize allocation, the risk of bias was unclear. If the authors described an appropriate method used to achieve randomization, the risk of bias was low. If the authors did not randomize allocation, the risk of bias was high. The second question in the Cochrane tool relating to allocation concealment (“Describe the method used to conceal the allocation sequence in sufficient detail to determine whether intervention allocations could have been foreseen in advance of, or during, enrollment.”) was dropped because it was irrelevant to the current review. We also added a comment to the “Other Bias” section of our tool. This comment asked the reviewers to consider pseudo-replication (repeated measures on non-independent units) and multiplicity, if relevant, during the risk of bias assessment.

### Summary measures

The primary reported measures of effect were ORs, prevalence ratios, and mean differences. The untransformed regression coefficients from linear models were used when these measures were the only measures reported by the authors.

### Planned methods of quantitative analysis

The protocol specified that we would perform a meta-analysis of each health metric (e.g., combine all upper respiratory disease outcomes, combine all gastrointestinal outcomes). The data evaluation revealed that this approach was not feasible because of the presence of multiplicity and non-comparability of outcomes. Therefore, the final approach to analysis was as follows: we grouped the data using broad outcome categories (i.e., lower respiratory tract, upper respiratory tract, MRSA, other infectious disease, neurological, psychological, dermatological, otologic, ocular, gastrointestinal, stress and mood, and other non-infectious health outcomes). Most measures of outcome were easily categorized. One exception was measurement of IgE, which was originally classified as both an upper and a lower respiratory outcome. Because each of the other outcomes was assigned to one category, we decided to group IgE with the upper respiratory outcomes for presentation purposes.

The extracted data were grouped into figures based on the reported effect measure. When applicable, they were also subgrouped within forest plots based on four risk of bias groups (i.e., subjective outcome/subjective exposure, subjective outcome/objective exposure, objective outcome/subjective exposure, and objective outcome/objective exposure). We did not calculate summary effect measures or perform quantitative assessments of heterogeneity. The open software package R’s ggplot command was used to create figures [5].

### Quantitative risk of bias across studies

No quantitative assessment of risk of small-study effects was performed due to the variations in effect measures, outcomes, and exposures.

### Summation of quality of evidence

We proposed to use the GRADE (Grading of Recommendations Assessment, Development and Evaluation) approach to summarize the body of evidence for each outcome category [6]. The GRADE categories are risk of bias, consistency, indirectness, precision, and publication bias.

### Additional analyses

Additional assessment of the potential for selective reporting bias included a comparison of findings reported in the abstracts with findings reported in the full texts. The purpose of this additional analysis was to compare the directions of the reported inferences between the abstracts and the full reports. For the abstract assessment, we counted the total number of results reported and the number of these results that indicated a harmful association with the risk factor. Harmful associations were deduced from the language used, reported *p* values <0.05 for harmful associations, or reported point estimates for associations for which the direction implied a harmful effect of exposure. For the full-text assessment, we counted the total number of ORs reported and the number of these ORs that indicated a harmful association with the risk factor. Restriction to ORs ensured that the direction of the association was clear (i.e., ORs > 1.0 indicated a risk effect). Such consistency is not possible when beta coefficients are used. For example, when the beta value represents a measure of function, then a negative number may indicate reduced lung function and, therefore, a risk event. If the beta value represents a log OR, then a positive number would result in an OR > 1.0 and also represent a risk event. This approach for selective reporting assessment was not included in the original protocol.

## Results

### Study selection

**Fig. 1:**
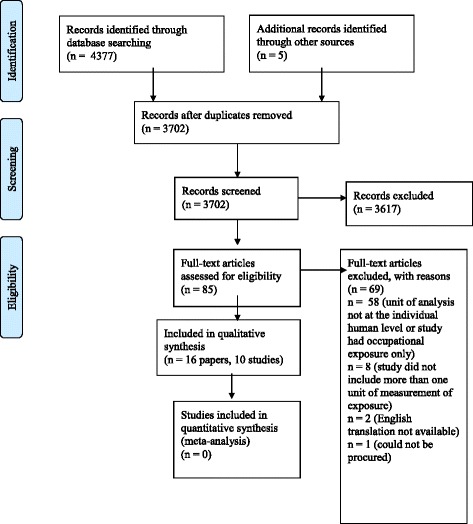
PRISMA (Preferred Reporting Items for Systematic Reviews and Meta-Analyses) flow diagram of the results of database searches for a systematic review of the associations between proximity to animal feeding operations and the health of individuals in nearby communities [32, 33]

The results for the total number of records screened, assessed for eligibility, included in the review, and excluded from the review, and the reasons for exclusion at Level 2, are presented in Fig. 1. The complete search strategies for each database, including the number of hits per line, are available elsewhere (ISU DD URL). The database searches retrieved 4377 records; 3697 records remained after de-duplication. The source of these records is available (ISU DD URL). Reference checking of relevant manuscripts retrieved an additional five records. Reasons for exclusion of records at Level 2, with citation information, are available (ISU DD URL). A total of 16 manuscripts and 10 study populations were identified for inclusion in the review.

### Study characteristics

The results for the characteristics of the 16 manuscripts and the 10 study populations are presented in Table 1 and Additional file 1: Table S2. Mixed populations of males and females comprised the study population for all the included studies. Only Bullers [7] reported the socioeconomic status of the subjects and stated that “most residents are in the low- to middle-income categories.”

**Table 1:** Summary of characteristics of studies included in a systematic review of the associations between proximity to animal feeding operations and the health of individuals in nearby communities

Study	Source	Study design	Country	Year(s) of study	*N* (study pop)	Age (study pop)	Statistical methods used to assess association(s) between exposure(s) and outcome(s)
Feingold et al. 2012 [17]	As for study	Observational: case-control	The Netherlands	2008–2011	87	NR	Multivariable approaches to dichotomous data with no fixed or random effects: logistic regression
Schiffman et al. 2005 [34]	As for study	Experimental (laboratory)	USA	NR	48	19–49 years	Multivariable approaches to categorical data with fixed and/or random effects: logistic regression/generalized linear model with logit link
							Multivariable approaches to continuous outcomes with fixed and/or random effects
The Netherlands study	Smit et al. 2014 [16]	Observational: cross-sectional	The Netherlands	2009	92,548	0–70 years	Multivariable approaches to categorical data with fixed and/or random effects: logistic regression/generalized linear model with logit link
	Smit et al. 2012 [19]	Observational: cross-sectional	The Netherlands	2009	92,548	0–70 years	Univariate approaches to categorical data: chi-square tests
							Multivariable approaches to categorical data with fixed and/or random effects: logistic regression/generalized linear model with logit link
Lower Saxony Lung Study	Schulze et al. 2011 [10]	Observational: cross-sectional	Germany	2002–2006	457	18–44 years	Multivariable approaches to dichotomous data with no fixed or random effects: logistic regression
							Multivariable approaches to continuous data with no fixed or random effects: linear regression
	Radon et al. 2007 [13]	Observational: cross-sectional	Germany	2002–2004	2425	18–45 years	Multivariable approaches to categorical data with fixed and/or random effects: logistic regression/generalized linear model with logit link
	Radon et al. 2005 [20]	Observational: cross-sectional	Germany	NR	6837 (questionnaire), 2812 (clinical assessment)	18–44 years	Multivariable approaches to categorical data with fixed and/or random effects: logistic regression/generalized linear model with logit link
AABEL Study	Hoopmann et al. 2006 [11]	Observational: cross-sectional	Germany	2001	7943 (questionnaires), 5136 (skin exam), 1552 (Phadiatop test)	5–6 years	Multivariable approaches to categorical data with fixed and/or random effects: logistic regression/generalized linear model with logit link
Schinasi et al. 2014 [18]	As for study	Observational: case-control	USA	2011	121 MRSA-positive patients and 122 MRSA-negative patients	18–65 years	Multivariable approaches to categorical data with fixed and/or random effects: logistic regression/generalized linear model with logit link
Mirabelli et al. 2006 [14]	As for study	Observational: cross-sectional	USA	2004	128,568	12–14 years	Multivariable approaches to categorical data with fixed and/or random effects: logistic regression/generalized linear model with logit link
Bullers 2005 [7]	As for study	Observational: cross-sectional	USA	1998–1999	82 (48 exposed, 34 unexposed)	Mean 57 years (exposed); mean 42 years (unexposed)	Univariable approaches to continuous outcomes (*t* test, ANOVA)
Schiffman et al. 1995 [35]	As for study	Observational: cohort	USA	NR	88	52 ± 13 years	Univariable approaches to continuous outcomes (*t* test, ANOVA)
CHEIHO Study	Avery et al. 2004 [21]	Observational: cross-sectional	USA	NR	15	33–77 years	Multivariable approaches to continuous outcomes with fixed and/or random effects
	Schinasi et al. 2011 [8]	Observational: cohort	USA	2003–2005	101	19–90 years	Univariable approaches to continuous outcomes (*t* test, ANOVA)
	Horton et al. 2009 [9]	Observational: cohort	USA	2003–2005	101	19.2–89.5 y	Multivariable approaches to categorical data with fixed and/or random effects: logistic regression/generalized linear model with logit link
	Wing et al. 2013 [22]	Observational: cohort	USA	2003–2005	101	Median 53 years	Multivariable approaches to categorical data with fixed and/or random effects: logistic regression/generalized linear model with logit link

*NR* not reported

### Risk of bias within studies

Results for the risk of bias within studies are presented in the figures along with the results for individual studies for each outcome category. The overall risk of bias for each study is presented in the last column on the right and is based on the highest risk of bias from the assessment of the separate bias domains. Because risk of bias is an outcome-level variable, the overall risk may vary within a study. For example, the Schinasi et al. [8] study had some outcomes that were assessed to have a serious, while others had a critical, risk of bias for lower respiratory disease. Some Horton et al. [9] study outcomes were assessed to have a serious risk of bias for psychological outcomes, and others were assessed to have critical risk of bias for stress-related outcomes. These differences reflected the types of approaches (i.e., subjective or objective) used to measure exposure and outcome variables.

### Results of individual studies

A total of 532 outcome and exposure relationships were extracted from the 16 studies. This result does not necessarily represent a complete list of all reported outcomes, because authors occasionally wrote only narratively about the associations or the variances could not be determined from the data presented (i.e., only the effect size or direction were reported).

The outcome categories reported by each manuscript are reported in Table 2. We collected and graphed data for all outcome groups. However, in the main text, we report the results for the outcome groups lower respiratory tract, upper respiratory tract, MRSA, and other infectious disease. The results for the neurological, psychological, dermatological, otologic, ocular, gastrointestinal, stress and mood, and other non-infectious health outcome groups are presented in Additional file 1. The results for respiratory outcomes are included in the main text because they were the most frequently assessed variables and represent the main outcome groups studied. We included MRSA and other infectious disease outcomes in the main text because these outcomes were identified by the updated review. The results for the other outcomes are presented in Additional file 1 because fewer results were different from the results of the prior review.

**Table 2:** Manuscripts included in the review (including Additional file 1) and the outcome categories reported by the investigators

Categorized class of outcome	Manuscript	Number of effect measures or *p* values reported
Antimicrobial resistance	Schinasi et al. [18]	13
	Feingold et al. [17]	3
Dermatologic	Schinasi et al. [8]	4
Eye	Schinasi et al. [8]	18
	Schiffman et al. [34]	2
Gastrointestinal diseases Lifestyle Lower respiratory	Schinasi et al. [8]	21
	Schiffman et al. [34]	2
	Mirabelli et al. [14]	2
	Smit et al. [16]	38
	Smit et al. [19]	9
	Schulze et al. [10]	3
	Schinasi et al. [18]	53
	Radon et al. [13]	32
	Hoopmann et al. [11]	6
	Mirabelli et al. [14]	89
	Radon et al. [20]	8
	Schiffman et al. [34]	4
Neurologic	Schinasi et al. [8]	14
	Horton et al. [9]	4
	Schiffman et al. [34]	2
Other	Smit et al. [19]	9
	Schinasi et al. [8]	21
	Schiffman et al. [34]	1
Otologic	Schinasi et al. [8]	7
Psychological	Horton et al. [9]	12
	Schiffman et al. [34]	8
	Schiffman et al. [35]	6
	Bullers [7]	3
Stress	Horton et al. [9]	4
	Schiffman et al. [34]	5
	Avery et al. [21]	4
	Schiffman et al. [35]	1
	Wing et al. [22]	48
Upper respiratory	Smit et al. [16]	13
	Schulze et al. [10]	2
Upper respiratory	Schinasi et al. [8]	29
	Radon et al. [13]	16
	Hoopmann et al. [11]	1
	Schiffman et al. [34]	15
Total		532

### Lower respiratory tract outcomes

**Fig. 2:**
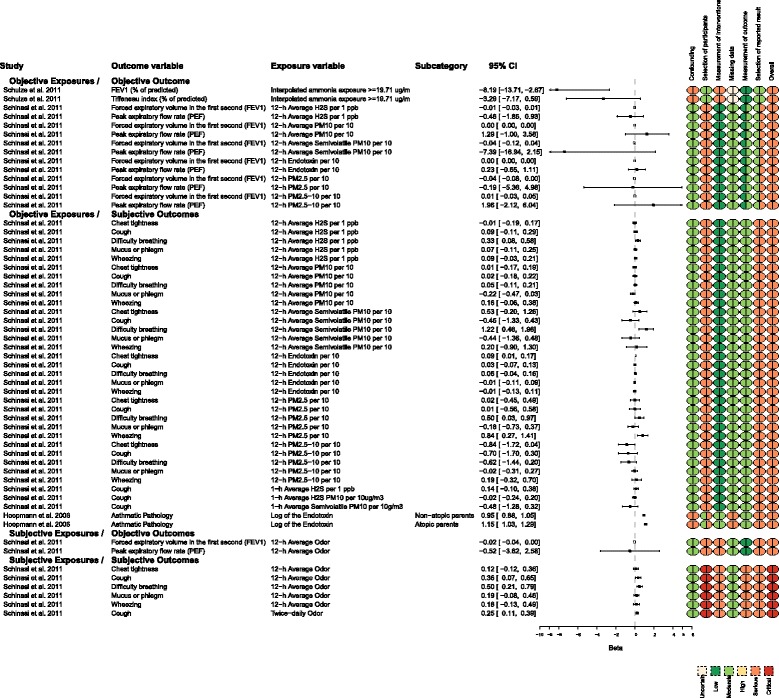
Measures of exposure and lower respiratory tract outcomes for which the effect size was reported as a regression coefficient (*β*)

**Fig. 3:**
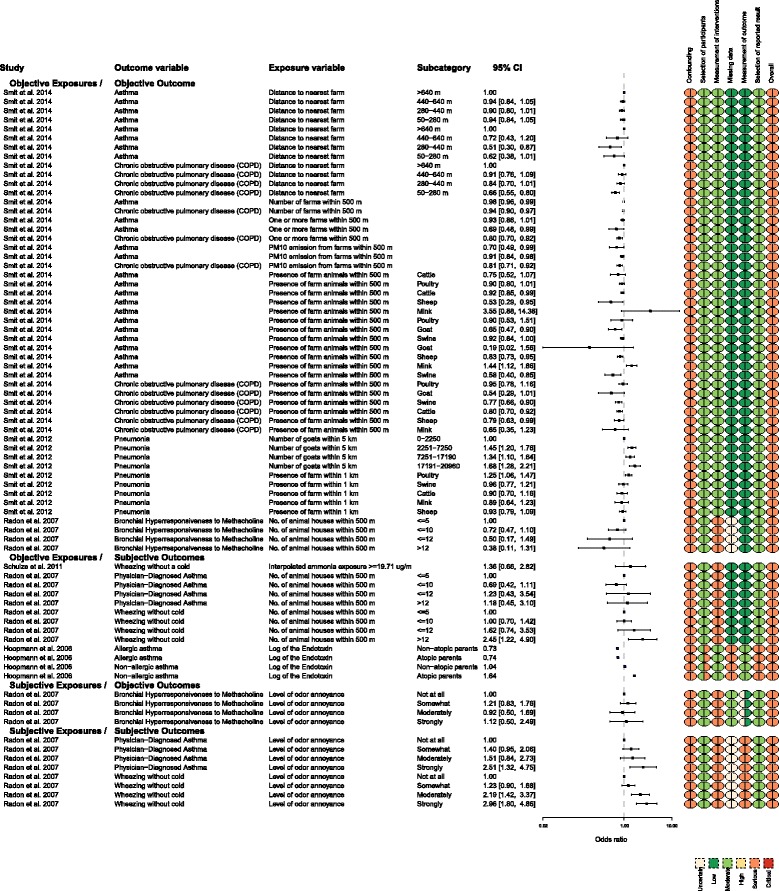
Measures of exposure and lower respiratory tract outcomes for which the effect size was reported as a prevalence odds ratio

Many studies reported outcomes associated with the lower respiratory tract [8, 10–14]. The reported effect measures were either regression coefficients (*β*s) (Fig. 2) or prevalence ORs (Fig. 3) and prevalence ratios [14]. Most of the extracted outcomes were taken from Schinasi et al. [8] and Mirabelli et al. [14]. With regard to Schinasi et al. [8], the article included results for multiple lower respiratory outcomes and multiple measures of exposure (Fig. 2). The authors reported the regression coefficients for every model. The scales of the regression coefficients differed between the outcomes. For example, some regression coefficients represented parameter estimates from a logistic model. Typically, these regression coefficients would have been converted to ORs. However, the authors did not present OR values and did not indicate the approach used to code categorical variables. Therefore, we were unable to convert these regression coefficients to ORs [15]. Other regression coefficients represented a one-unit change in unspecified units for the continuous metric (e.g., forced expiratory volume and peak expiratory flow rate). Overall, 146 regression coefficients were reported by Schinasi et al. [8].

Most of the regression coefficients reported for lower respiratory tract outcomes included a 95% confidence interval (CI), which included effect sizes associated with protective effects, risk effects, and no effect (i.e., the 95% CI included the null value). Three regression coefficient values had negative value beta estimates (<1.0), which suggested the presence of a risk effect of exposure on the outcome. However, the estimates lacked precision; they all had very wide CIs that included the null value. The values for these three intervals are presented in the objective exposures/objective outcomes subgroup results in Fig. 2. It was difficult to compare the precision with which the effect sizes were estimated because the scales of the underlying data informing the effect size differed across studies and outcomes within studies. The overall risk of bias was considered to be serious or critical for the studies that provided outcomes measured as regression coefficients.

There was no consistent evidence of an association between exposure (or higher levels of exposure) to animal facilities and higher odds of lower respiratory tract outcomes for the prevalence OR effect measure, except when the level of odor annoyance was used as the measure of exposure (subjective exposures/subjective outcomes subgroup) (Fig. 3). When proximity to animals was used as an objective measure of exposure and associated with an objective measure of lower respiratory health (bronchial hyper-responsiveness to methacholine), the higher levels of exposure were associated with numerically lower odds of disease (≤12 farms within 500 m of the subject’s residence, OR = 0.5, 95% CI 0.17, 1.49; >12 farms within 500 m, OR = 0.38, 95% CI 0.11, 1.31) [13] (Fig. 3). The precision of the effect size estimates was low (i.e., the intervals were wide), and the 95% CIs extended across a range that included a protective effect, no effect, and a risk effect. Mirabelli et al. [14] reported 89 prevalence ratios (PR) and these are reported in Additional file 1, and the same inference applies. Most prevalence ratio intervals included one, and no consistent dose-response effect was observed. For example, when the measure of exposure was distance to a swine operation, the comparison of no exposure to the most exposure (<2 miles), to middle exposure (between 2 and 3 miles), and to the lowest exposure had a PR of 1.01 (0.95–1.07), 1.12 (1.04–1.19), and 1.05 (1.00–1.10), respectively, in children with self-reported allergies. However, for the same population and a different exposure metric, a different association was observed. For example, when the measure of exposure was hog pounds (in millions) within 3 miles of school, the comparison of no exposure to the lowest level of exposure (from 0.1 to <0.2 million pounds) was associated with a higher point estimate of the prevalence ratio for current wheeze in children with self-reported allergies (PR = 1.97, 1.01–1.12), yet the comparison of no exposure to higher levels of exposure (>5.0 million pounds) had a PR of 1.0 (0.89–1.11). (See Additional file 1 for these data.)

When odor annoyance was compared with an objective measure of lower respiratory health (bronchial hyper-responsiveness to methacholine), the moderate and strong levels of annoyance exposure categories did not indicate a consistent dose-response direction (odor annoyance: somewhat, OR = 1.21, 95% CI 0.83, 1.76; moderate, OR = 0.92, 95% CI 0.5, 1.69; strong, OR = 1.12, 95% CI 0.5, 2.48) [13] (Fig. 3). The precision of these effect size estimates was low, and the 95% CIs extended across a range that included a protective effect, no effect, and a risk effect.

Many authors studied ordered levels of exposure (increasing or decreasing) to document a dose-response, which is important for investigation of causation (Fig. 3). The results for ordered levels of exposure are presented in Fig. 3. The referent exposure used by the authors is indicated with an OR = 1. When objective measures of exposure and outcome (the first subgroup in Fig. 3) were evaluated, there was consistent evidence that measures of higher exposure or closer proximity were associated with effect sizes that were numerically protective of the outcome (<1). However, the precision of these effect sizes meant that the CIs included values that indicated reduced, no difference in, and increased prevalence. For example, when asthma was associated with the highest level of exposure (closest proximity to the farm), the OR point estimate was 0.9 and the 95% CI was 0.8 to 1.01 [12].

The presence of goats within 500 m of the subject’s residence was numerically protective for asthma (OR point estimate = 0.19). However, the precision of this estimate was wide (95% CI 0.02, 1.56). When the metric for goat exposure was a density indicator (i.e., number of goats within 5 km of the subject’s residence) and the outcome metric was pneumonia, there was evidence of an association between higher goat density and lower respiratory disease. The prevalence OR for the highest goat density (17,191–20,960) was 1.68, which indicated an increased prevalence of disease. Although the precision was moderate, all of the values within the 95% CI were associated with increased prevalence. These apparently inconsistent findings were reported by the same authors in the same study population [12]. One explanation is that different mechanisms lead to the development of pneumonia versus asthma. The study was performed during a Q fever outbreak, and the finding suggested that exposure to goats was strongly associated with Q fever risk. The authors used pneumonia as a potential Q fever-related outcome, because pneumonia was the most frequent diagnosis among the notified Q fever patients in the Netherlands epidemic. The authors also noted that exposure to poultry was associated with increased prevalence odds of pneumonia. This association between goats and pneumonia was likely due to Q fever, rather than particulate or gaseous emissions.

The overall risk of bias was serious for all of the studies that reported prevalence ORs as measures of association (Fig. 3).

### Upper respiratory tract outcomes

**Fig. 4:**
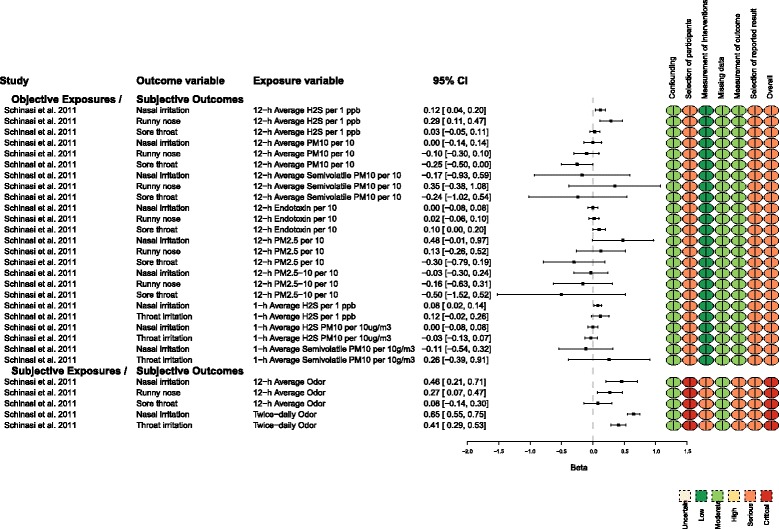
Measures of exposure and upper respiratory tract outcomes for which the effect size was reported as a regression coefficient (*β*)

**Fig. 5:**
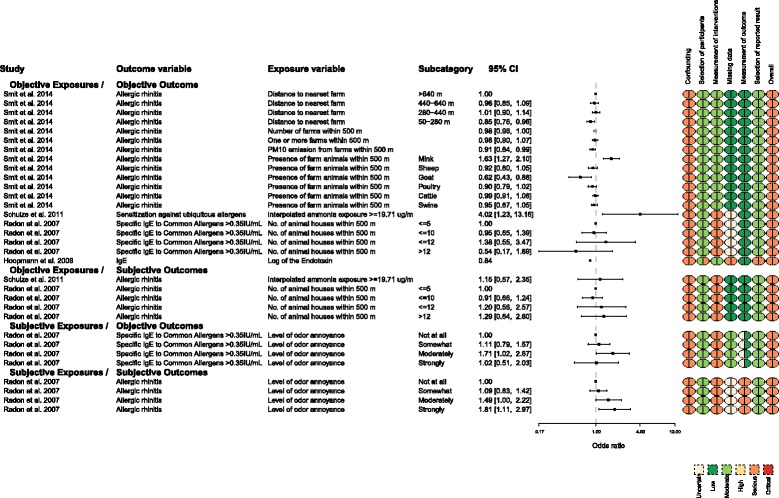
Measures of exposure and upper respiratory tract outcomes for which the effect size was reported as an odds ratio

Measures of upper respiratory tract health were commonly reported outcome variables. The measures of association reported were regression coefficients and prevalence ORs. These data are presented in Figs. 4 and 5. Schinasi et al. [8] was the only article that included data. They presented regression coefficients (*β*), and the interpretation of these coefficients differed, because some were from logistic and others were from linear models. There were inconsistent associations between the objective measures of exposure and the subjective measures of upper respiratory tract outcomes (Fig. 4). In some cases, the regression coefficients indicated increased disease at higher levels of exposure, which suggested that exposure was associated with increased disease or symptoms. In other cases, the regression coefficients for exposure indicated the presence of protective effects. For objectively measured exposure metrics and subjectively measured outcomes (first subgroup, Fig. 4), most effect sizes had levels of precision that were associated with protective effects, no effect, or risk effects. There were consistent associations between subjectively measured exposures (average 12-h odor levels and twice-daily odor levels) and outcomes (i.e., increased odor was associated with increased disease) (second subgroup, Fig. 4). Large positive effect sizes indicated higher values for measures of upper respiratory tract outcomes in participants who indicated that they were exposed to higher odor levels. For this subgroup, all of the values in the 95% CI were associated with increased prevalence, except for the sore throat outcome.

Four studies used prevalence ORs to measure associations between exposure to animal facilities and upper respiratory tract outcomes [10, 11, 13, 16] (Fig. 5). The associations were not consistent for the subgroups objective exposure/objective outcome, objective exposure/subjective outcome, and subjective exposure/objective outcome. The findings across dose gradient were also inconsistent. Some results indicated that compared with high-level exposures, lower- or medium-level exposures were associated with higher odds of disease.

When the measure of exposure was the level of odor annoyance, a dose-response was present if the outcome was measured subjectively (self-reported symptoms). The association was inconsistent when an objective measure of disease occurrence was assessed (specific IgE to common allergens). Moderate annoyance at odor was associated with the highest specific IgE response relative to “not at all annoyed” by odor. The precision of the result suggested the direction was consistently positive (OR = 1.71, 95% CI 1.02, 2.87). However, compared with “not at all annoyed,” individuals who responded “somewhat annoyed” (OR = 1.11, 95% CI 0.79, 1.57) and “strongly annoyed” (OR = 1.02, 95% CI 0.51, 2.03) had effect sizes more consistent with an interpretation of no effect (close to OR = 1). The precision of these estimates was low; the 95% CIs had values consistent with increased prevalence, no effect on prevalence, and decreased prevalence.

### MRSA outcomes

**Fig. 6:**
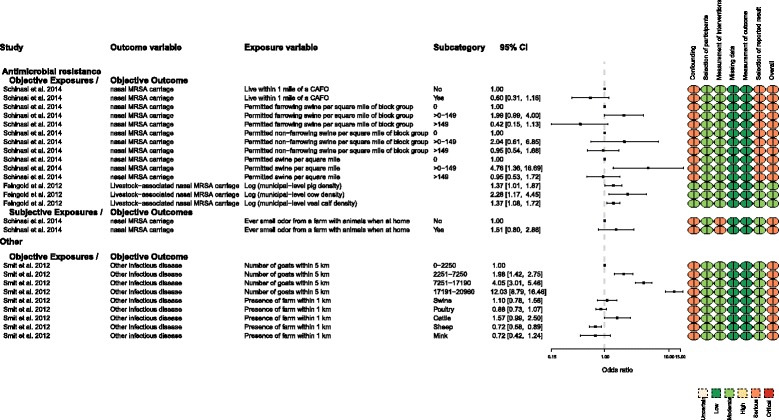
MRSA and “other infectious disease” outcomes for which the effect size was reported as an odds ratio

Two studies [17, 18] evaluated the association between human carriage of MRSA and proximity to animal feeding operations, which was a new outcome for this update. There was no consistent finding for this outcome. One study used a subjective measure of exposure (odor), and the patients were aware of their MRSA status at the time of exposure assessment. Schinasi et al. [18] found that the odds of being MRSA-positive were greater if the subject had ever smelled farm odors when at home (OR = 1.51) (Fig. 6). The precision of this estimate was moderate, but most of the effect sizes in the 95% CI were consistent with no effect (95% CI 0.8, 2.86). However, this association was not consistently observed for the same study participants when the exposure was an objectively measured metric, and the observed results did not document a consistent dose-response. For example, living <1 mile of an animal feeding operation was associated with a protective effect size for nasal MRSA carriage (OR = 0.6) [18]. However, the effect size precision was low and, for this outcome, most of the effect sizes in the 95% CI were consistent with no effect (95% CI 0.31, 1.16) (Fig. 6). Schinasi et al. [18] evaluated another exposure metric in the same population (the number of farrowing swine permitted within a 1-square-mile block of the participant’s residence) and explored whether a dose-response was present. However, when compared with the lowest exposure level, the middle exposure level had a higher prevalence of MRSA carriage than higher levels of exposure. For example, moderate exposure levels (between 0 and 149) were associated with a prevalence OR that suggested higher odds of nasal MRSA carriage in individuals, compared with individuals not exposed to swine (0). The authors reported moderate exposure using three metrics (farrowing swine, non-farrowing swine, and swine); the effect sizes and precision estimates were 1.99 (95% CI 0.99, 4.06), 2.04 (0.61, 6.85), and 4.76 (1.36, 16.69), respectively (Fig. 6). These estimates contrasted with higher levels of exposure (>149), which were associated with a prevalence OR that suggested lower odds of nasal carriage of MRSA. The authors used the three metrics to evaluate high levels of exposure; the effect sizes and precision estimates were 0.42 (95% CI 0.15, 1.13) for farrowing, 0.95 (95% CI 0.54, 1.68) for non-farrowing, and 0.95 (95% CI 0.53, 1.72) for swine (Fig. 6). Feingold et al. [17] did report an association between MRSA carriage and swine (1.37, 95% CI 1.01, 1.67), cattle (2.28, 95% CI 1.17, 4.15), and veal (1.37, 95% CI 1.08, 1.72) density. They did not report any inconsistencies in association because they only reported one exposure metric (the log of municipal density). It is unclear whether other exposure metrics were evaluated but not reported.

The overall risk of bias was serious for all MRSA outcomes reported by Schinasi et al. [18] and moderate for the outcome reported by Feingold et al. [17] (Fig. 6).

### Other infectious disease outcomes

This “other infectious disease” category of health outcome (Fig. 6) includes data from Smit et al. [19], who used this term. Smit et al. [19] reported a strong association and clear dose-response between goat density and “other infectious disease.” However, it was actually unclear what this outcome represented, or if it duplicates a prior analysis by these authors, which evaluated respiratory disease outcomes. Smit et al. [19] described this variable as: “In the Netherlands, Q fever is registered by GPs under the ICPC code ‘other infectious disease’ (A78). Despite the broad name, ‘other infectious disease’ is normally only used for patients with Q fever or Lyme disease.” This description suggests that during a Q fever outbreak, this “other infectious disease” would likely be Q fever, which is often but not always associated with respiratory disease.

The overall risk of bias was serious for the single study that reported “other infectious disease” outcomes (Fig. 6).

### Synthesis of results

No quantitative synthesis of the results was possible because of the diversity of metrics used to measure exposures and outcomes, even within a subgroup. The use of correlated outcomes and exposures also complicated the synthesis process.

### Risk of bias across studies

Risk of bias across the body of work was considered. First, with respect to publication bias, because a meta-analysis was not performed, we did not include a quantitative evaluation of the effect of small-study effects on outcomes. Another source of bias across the body of work was the potential for confounding to partly explain the findings. All but one of the studies were observational, so the potential for uncontrolled or residual confounding to affect the results of numerous studies across the entire body of work was significant. Because the studies were cross-sectional in nature, adjustment for confounders (even in regression models) would not provide protection against residual confounding. Overall, the assessment was that there was a high risk of bias for the entire body of work. This conclusion was based on the results of risk of bias from the individual studies.

### Additional analyses

Of 58 results reported in abstracts, 55 (95%) reported on risk effects of exposure (many with odor as the measure of exposure). The lack of an association was reported for three outcomes, and there were no protective associations. For comparison purposes, with respect to ORs, where values >1.0 always indicated increased harm associated with exposure, of 223 OR point estimates, 98 (44%) were ≤1.0 and 125 (56%) were >1.0.

### GRADE category: risk of bias within studies

Most of the studies used a cross-sectional observational design. Therefore, the major risk of bias within the studies related to use of prevalent cases instead of incidence as the outcome and to confounding.

With respect to measurement error, many outcomes were measured within studies, with varying misclassification risk. Some investigators used objective measures of disease (Lower Saxony study population) [10, 13, 20], while other investigators used solely self-assessed (and often recall-based) measures of disease (CHEIHO Study) [8, 9, 21, 22]. The potential for disease status mismeasurement was strongly suspected when subjective recall of disease was used. The expected direction of bias was away from the null, and >1.0 (increased disease with increased odor); the results were consistent with this assumption.

It is difficult to measure exposure to animal feeding operations. Some authors used proxies of exposure (e.g., animal distance from the subject’s residence or animal density around the subject’s residence). However, these proxies do not account for topography or wind direction, which affects true exposure. Some authors used odor as a subjective measure of exposure. Other authors used measures of particulate matter, or emissions such as ammonia, which can be direct measures of exposure. The Lower Saxony study originally used exposure data based on animal housing density [13, 20, 23]. In the new publication identified for this review from the Lower Saxony study [10], the investigators attempted to address the concerns about mismeasurement by using ammonia values based on measurements at 22 sampling sites and imputed values based on a computer algorithm on a subset of the population. However, the ammonia measurements were collected at least 1 year after the health outcomes were measured [10], so concerns about mismeasurement persisted. Overall, these problems with exposure measurement resulted in an increased risk of bias. The direction of bias was difficult to assess.

With respect to mismeasurement, it is often possible to argue for bias towards the null. However, we would argue that when subjective measures are paired (e.g., subjective measures of exposure such as odor and subjective measures of outcome such as self-reported disease), the direction of bias is likely non-differential and away from the null. The final concern was the effect of mismeasurement on confounding. Mismeasurement increased the potential for residual confounding and, therefore, for bias.

Selection bias was the third risk domain of concern for the studies included in the review. When a concern was identified, it was generally because we suspected that the approach to enrollment would have enrolled a higher proportion of the exposed/diseased population than the other populations and biased the observed associations away from the null (increasing adverse outcomes).

### Evidence of consistency

We considered three ways that an association could be consistent: (1) within an exposure measure (i.e., a consistent dose-response between exposure and disease), (2) within a study across outcomes (i.e., a consistent association with disease across measures of different exposures within a study), and (3) a consistent association between exposure and the disease across studies.

With respect to dose-response, there was no consistent evidence of an association unless odor was the exposure measure and self-reported disease was the health outcome. For other approaches to measuring exposure and disease, the possible dose-responses included:

- Evidence that increased exposure to animal operations reduced disease odds (COPD and distance to the nearest farm) (Fig. 3) [16]
- Evidence that increased exposure to animal operations increased disease odds (wheezing without cold and number of farms within 500 m) (Fig. 3) [13]
- Evidence of no association between levels of exposure to animal operations and disease odds (physician-diagnosed asthma and number of farms within 500 m) (Fig. 3) [13]
- U-shaped associations, which suggested the middle range of exposure was more protective of disease than high levels of exposure (asthma and distance to the nearest farm) (Fig. 3) [16]

Similarly, there were findings for upper respiratory disease outcomes:

- Evidence that increased exposure to animal operations reduced disease odds (allergic rhinitis and distance to the nearest farm) [16]
- U-shaped relationship, which suggested the middle range of exposure was associated with more disease than high levels of exposure (specific IgE to common allergens and level of odor annoyance (Fig. 5) [13]

The one finding that was consistent was the relationship between subjectively measured exposure metrics (i.e., odor) and subjectively measured health outcomes. More odor was associated with more self-reported adverse health outcomes. However, as an indication of the inconsistency in this body of evidence, when objective measures of exposure were used, the associations observed did not reflect the relationships. For example, for the same exposure level of annoyance, there was a strong dose-response for self-reported wheezing, but bronchial hyper-responsiveness to methacholine showed almost no evidence of an association.

### Precision

There were no conclusions about precision because no summary effects were calculated for the variety of exposure/outcome combinations. No panel was available to determine the optimal information criteria for each outcome and exposure metric. Some estimate intervals were very wide, and others were very narrow. It was very difficult to assess the meaning of precision for results reported as beta values. Some of these betas represented the change in the log of the odds of disease, and some values represented one-half the change in the log of the odds of disease (if deviation from mean coding was used). Other values represented the change in a one-unit scale for an exposure such as parts per billion with an outcome such as blood pressure.

### Directness/applicability

The body of work included populations from a variety of countries. We assumed that the characteristics of the people living near animal feeding operations have not changed significantly over the 20 years encompassed by the studies. How directly the livestock emissions measured in the older studies relate to more current exposures is unclear. Some studies are old (>10 years). Environmental regulations may have changed, and animal housing and manure management may have also changed dramatically. Levels of exposure may now be different. For example, it is unclear how applicable the results from the CHEIHO Study (study interval 2003–2005) are to current community members in North Carolina, the rest of the USA, or European communities currently living near agricultural production. The same can be said for the Lower Saxony Lung Study finding. A panel that aims to develop recommendations could determine if approaches used for housing and pollution control have changed significantly during the intervening period. Other reviews of this topic include populations exposed to other forms of air pollution, such as coal dust or traffic dust, or include animal models of pollution exposure. The decision about the applicability of these studies to the research question is based on judgment. We chose to not include these studies because of the difficulties with applicability.

### Publication bias

A quantitative assessment of publication bias was not possible. Our qualitative assessment suggested that selective reporting was not a large concern in this body of work, unless one considers the results reported in the abstracts. The abstracts of the included papers overwhelmingly reported outcomes that were statistically significant or had effect estimates that indicated harm associated with exposure, or reported general harm without reference to point estimates or *p* values. This pattern suggested that although the full papers had fewer issues related to selective reporting, the abstracts may have over-emphasized the negative effects of exposure.

### Other potential biases—multiplicity

A final comment about the literature in the area concerns the effect of multiplicity. Nearly 500 outcomes were extracted from the 16 publications and 10 study populations. However, more than 500 outcomes were mentioned in all the publications, combined. For several study populations, numerous correlated outcomes were compared with numerous correlated exposures. This approach increases the potential to discover important associations and also increases the potential for identification of false associations due to random error (increased type 1 error). The great advantage of this body of work, however, is that in the full text the authors appear to have been transparent about the number of hypotheses tested, but perhaps less so in the abstracts. This level of transparency is commendable because comprehensive reporting enables a more comprehensive assessment of the effect of multiple testing in the full text. Given the hypothesis-generating and exploratory nature of many of the studies in this body of work (cross-sectional studies of prevalent outcomes and no studies “powered” to a particular outcome), many would also argue that testing and reporting the results of multiple tests is appropriate. The alternative problem of publication bias based on statistical significance would be a more critical issue. Approximately 90% of assessed outcomes did not report a CI that excluded the null value. If publication was based only on 95% CIs that excluded the null (i.e., *p* values <0.05), the conclusions about association might have been different.

## Discussion

### Summary of evidence

The largest body of evidence associating animal feeding operations with measures of health was available for lower respiratory disease outcomes (Figs. 2 and 3). There were no associations between exposure and disease for objective measures of disease and objective measures of exposure. No consistent dose-response relationships between exposure and disease were found. The second most commonly assessed outcome was upper respiratory tract outcomes, and there was no evidence of an association between exposure and disease for any objective measures of disease and objective measures of exposure. No consistent dose-response relationships between exposure and disease were found.

We found that for the infectious disease outcome, Q fever, the evidence suggested a strong relationship between development of Q fever (and symptoms associated with Q fever) and proximity to goat production facilities. The MRSA outcome was a new outcome for this review. No conclusions were possible because the study results were inconsistent. Feingold et al. [17] did find evidence of a weak association between proximity and MRSA, with moderate risk of bias. However, [18] evaluated a dose-response and consistently found that community members closer to animal feedings operations, or with more exposure, had lower odds of being MRSA-positive. This study has a serious risk of bias, but the main concern was confounding and selective reporting of results.

The number of studies available to assess the remaining outcome categories was very small; often only one study was available. The potential for bias was considered to be critical or serious. Consequently, no conclusions about associations between exposure to livestock operations and those health outcome categories were possible.

The information provided in our review should be useful to other researchers or funding agencies as they seek to prioritize the next steps for study or decision-making. Our review presents all the data in one location and includes our assessment of the potential for bias. Because we used a comprehensive rather than a selective approach, interested community and industry groups can see all of the outcomes measured and our interpretations of the potential for bias. These groups may then apply the information to their communities.

Funding for this review was provided by the National Pork Board, so there may be some concerns about conflicts of interest. We attempted to alleviate these concerns. First, we were explicit about the eligibility criteria a priori and published the protocol [2]. Second, we did not exclude any exposures or disease outcomes from the review. We used a transparent approach to present the results for the associations. We did not rank health outcomes as critical, important, or not important. Third, we did not make recommendations. We presented the work in a comprehensive manner that included explicit reporting of the magnitudes of the effects and of our assessments of the risks of bias.

Some findings published during the period between our previous version of this review and the update are worthy of discussion. Two studies of one population found that goats are likely associated with increased risk of Q fever in surrounding communities (Smit et al. [19] and Morrow et al. [12]). We concluded that this association is likely a causal one, because the documentation of a dose-response and a strong effect increased confidence in the conclusion. This association is likely causal in community members, not just in individuals who experience occupational exposure. Smit et al. [19] and Morrow et al. [12] assessed pneumonia, asthma, and “other infectious diseases.” According to the authors, pneumonia and “other infectious diseases” are likely measures of Q fever. Interestingly, asthma, which we would not expect to be associated with Q fever, but with a different mechanism (likely particulate matter), was not associated with goat density. As this review was being finished, five more articles have been published by the same group, using what appears to be the same population [24–28]. Even if they were published earlier, some of these articles would not have been included in this review because different outcomes were examined (i.e., number of hospital visits, organisms in ambient air). Because the same study population was examined, it is unclear whether these new results contribute any new insights into the associations between Q fever, goats, and community health.

Assessment of the association between proximity to livestock operations and colonization with MRSA was identified as another new topic area. This association was evaluated by two studies [17, 18]. It was difficult to assess the likely causal associations between exposure to cattle (dairy or beef not specified) and MRSA because only one group [17] reported evaluation of this association. The associations with exposure to swine were not consistent, so it was difficult to assess the likelihood of a causal link. For example, the strongest consistent association with nasal MRSA colonization and exposure to swine was observed for the medium level of exposure. Higher levels of exposure were not associated with colonization. A dose-response relationship increases the potential for a causal association, so this finding decreased certainty of causality [29].

Additional studies reported more individual associations (beta and OR values) for the other health outcomes (upper and lower respiratory reported here, others in Additional file 1), but the conclusions of the prior review remained unchanged for these outcomes. Many of the new publications were based on the same study populations included in the prior review (albeit with new outcomes). The factors that limited the ability to make strong causal inferences were as follows: (1) the approaches used for enrollment often appeared to select for enrollment of exposed people of ill health who were identified based on association with grassroots activist groups, (2) the use of cross-sectional study designs and, therefore, prevalence of disease for many outcomes, and (3) inability to blind participants to exposure for many outcomes. Concerns about the effects of multiplicity on false discovery of significant findings still remain for this review update. This concern was present in the original review; >100 outcomes were assessed for some study populations. However, the authors of those studies were transparent about the numbers of outcomes assessed; they did not use selective reporting of significant outcomes.

### Limitations

It was necessary to change some aspects of the protocol for pragmatic reasons during the performance of the review. These changes were mostly related to summarization of the results. We originally proposed that we would perform a meta-analysis and use a process similar to GRADE to summarize the evidence [6]. We did not perform a meta-analysis because of the diversity of outcomes and exposures and GRADE also did not seem to provide a framework to address the multiplicity problem. One solution would have been to eliminate outcomes; however, as mentioned, perceptions of conflicts of interest meant we favored summarizing all the outcomes studied. Review performance did not deviate significantly from the protocol for the other aspects of the review.

This review topic has also been reviewed by others, but, to our knowledge, no other systematic reviews have been published. It is uncommon for other reviews to explicitly assess the risk of bias or to state explicit eligibility criteria. One review did discuss the limitations of the cross-sectional studies that included the use of prevalent outcomes [30]. The authors of that review concluded that “there was sufficient evidence of an association between living near IFAP (industrial food animal production) and respiratory outcomes, MRSA, Q fever, and stress/mood.” [30]. The review authors also urged the use of prospective designs to obtain stronger evidence; this approach would provide a way to reduce the biases present in the current body of work. Other reviews have reached stronger conclusions about the causal nature of the associations observed in these studies, in particular those that relate to respiratory disease. For example, the 2007 review of [31] concluded that the body of work was sufficient to say that “The current state of knowledge of community impacts of CAFOs warrants support for the American Public Health Association recommendation for a moratorium on all new CAFO construction.”

## Conclusions

This review revealed that there is sufficient evidence to conclude that communities living in proximity to goat production are at increased risk of Q fever. The association between MRSA colonization and proximity is unclear, mainly due to a lack of replication. The conclusions about associations with other outcomes, especially those related to upper and lower respiratory disease, are unchanged from the prior review: “There was inconsistent evidence of a weak association between self-reported disease in people with allergies or familial history of allergies. No consistent dose response relationship between exposure and disease was observable.” If questions about the health effects of living near animal production continue to be of interest, then large, long-term prospective studies will be required, especially if non-specific clinical symptoms are the outcomes of interest.

## Additional file

Additional file 1: Discussion about other outcomes included in the systematic review. Excel Spreadsheet with extracted data from review. Data extraction forms, risk of bias forms and search startergies used for review. **Figure S1.** Neurological and psychological symptoms and stress outcomes for which the effect size was reported as an odds ratio. **Figure S2.** Neurological symptoms for which the effect size was reported as a regression coefficient. **Figure S3.** Psychological outcomes for which the effect size was reported as a point estimate of the mean difference. **Figure S4.** Psychological outcomes for which the effect size was reported as a point estimate. **Figure S5.** Psychological outcomes for which the effect size was reported as a regression coefficient. **Figure S6.** Dermatologic, otologic, and optical outcomes for which the effect size was reported as a regression coefficient. **Figure S7.** Gastrointestinal and “Other” outcomes for which the effect size was reported as a regression coefficient (*β*). **Figure S8.** Stress outcomes for which the effect size was reported as a regression coefficient (*β*). **Figure S9.** Lower respiratory outcomes for which the effect size was reported as a prevalence ratio. (ZIP 1.40 mb)
