# Supplementary material for: Updated systematic review: associations between proximity to animal feeding operations and health of individuals in nearby communities
Source: Syst Rev. 2017 Apr 18;6:86. doi: 10.1186/s13643-017-0465-z (PMC5395850; doi:10.1186/s13643-017-0465-z)
Supplement: Additional file 1: — Discussion about other outcomes included in the systematic review. Excel Spreadsheet with extracted data from review. Data extraction forms, risk of bias forms and search startergies used for review. Figure S1. Neurological and psychological symptoms and stress outcomes for which the effect size was reported as an odds ratio. Figure S2. Neurological symptoms for which the effect size was reported as a regression coefficient. Figure S3. Psychological outcomes for which the effect size was reported as a point estimate of the mean difference. Figure S4. Psychological outcomes for which the effect size was reported as a point estimate. Figure S5. Psychological outcomes for which the effect size was reported as a regression coefficient. Figure S6. Dermatologic, otologic, and optical outcomes for which the effect size was reported as a regression coefficient. Figure S7. Gastrointestinal and “Other” outcomes for which the effect size was reported as a regression coefficient (β). Figure S8. Stress outcomes for which the effect size was reported as a regression coefficient (β). Figure S9. Lower respiratory outcomes for which the effect size was reported as a prevalence ratio. (ZIP 1.40 mb) [file 13643_2017_465_MOESM1_ESM.zip › Supplemental materialR1.docx]

# Additional file 1: Other outcomes included in the systematic review.

**Neurological outcomes.** Two publications provided estimates of associations of exposures with neurological outcomes (i.e., headache, dizziness, confused or unable to concentrate) (Horton et al. 2009; Schinasi et al. 2011). Both publications described the results for the participants of the CHEIHO Study, who were asked to self-report numerous outcomes. Horton et al. (2009) reported the associations as odds ratios (Figure S1). Schinasi et al. (2011) reported the regression coefficients (betas) from a logistic regression model (Figure S2). The direction of association was not consistent; sometimes the effect size indicated a protective effect or a risk factor. For the odor metrics, the effect measure was always in the direction that indicated increased risk of health outcomes in exposed individuals. The intervals of three (hydrogen sulfide, PM10, semivolatile PM10) of the four effect sizes reported by Horton et al. (2009) were associated with estimates that indicated risk, protection, or no association. The overall risk of bias for this exposure-outcome association was serious or critical. Horton et al. (2009) reported one effect measure for which the precision measure (95% confidence interval (CI)) only included estimates associated with harm: for the exposure "twice-daily odor rating" and the outcome "confused or unable to concentrate" (95% CI = 1.16, 1.50). The overall risk of bias for this exposure-outcome association was critical (Figure S1)

**Psychological outcomes.** Four publications with data from four studies reported associations between measures of exposure to animal feeding operations and psychological outcomes (Bullers 2005; Horton et al. 2009; Schiffman et al. 1995; Schiffman et al. 2005). The (Schiffman et al. 1995) and (Schiffman et al. 2005) articles only reported effect size estimates (no measures of variance) or p-values for associations (Figures S3, Figure S4, Table S3) . The Horton et al. (2009) study found effect sizes close to null values for psychological outcomes associated with objective measures of exposure. However, increased odds of adverse psychological outcomes were reported when the measure of exposure was based on the subjects' self-assessed 12-hour odor ratings (Figure S1). The overall risk of bias in all of these studies was high to critical. A critical concern with (Bullers 2005) study was the approach to the selection of participants, which was described as "a snowball sample of respondents who lived near industrial hog farms and had been identified by local grass-roots activists as individuals who were distressed about the effects of nearby hog farms" (Figure S5).

**Dermatological outcomes.** Only one study evaluated dermatological outcomes (Schinasi et al. 2011) (Figure S6). The investigators used a subjective self-assessment of skin irritation and measured associations against three similar objective measures of emissions from the facilities (1-hour averages of particulate matter and hydrogen sulphide levels) and one subjective measure of exposure (odor). Our major concern with the study was the approach to recruitment of participants, which was described as "Community-based organizations brought the issue to the attention of researchers at the School of Public Health at the University of North Carolina and have continued as partners in all research that has been conducted. In the CHEIHO Study members of community-based organizations participated as advisors in the study design and design of study instruments. They were integrally involved in the recruitment and training of study participants." We were also concerned about multiplicity, because Schinasi et al. (2011) used highly correlated exposure metrics to evaluate > 100 health outcomes.

**Otologic outcomes.** Only one study assessed an otologic outcome (difficulty hearing) (Schinasi et al. 2011) (Figure S6). The same outcome was compared with four correlated objective measures of exposure (12-h average H_2_S per 1 ppb, 12-h PM_2.5-10_ per 10 ppb, 12-h average PM_10_ per 10 ppb, and 12-h average semivolatile PM_10_ per 10 ppb), and two highly correlated subjective measures of exposure (average 12-hour odor and twice-daily odor). 12-h average semivolatile PM_10_ per 10 ppb was associated with an effect size that indicated increased risk (beta = 1.78, 95% CI 0.51, 3.05). However, for the same study participants, the other five exposure metrics had CIs that included the null, and four of those had point estimates less than the null value (0). These results were evidence of inconsistency. Similar to other findings from this study, the overall risk of bias was serious or critical mainly due to the approach used to recruit participants. We were also concerned about multiplicity, as previously discussed (Schinasi et al. 2011).

**Ocular outcomes.** Two studies assessed this outcome category. One study used an observational study design (Schinasi et al. 2011) (Figure S6). The other study used a cross-over experimental design, which included exposing participants to air from a swine confinement facility (Schiffman et al. 2005). The overall risk of bias was high for this outcome mainly because the participants knew their exposure status (i.e., they could smell the odor). For the observational study, the overall risk of bias for the ocular outcome was serious because of the approach used for study participant recruitment. The observational study used three highly correlated metrics of eye outcomes (burning eyes, eye irritation, and itching eyes) reported on the same participants (Figure S6). This study also used four highly correlated objective metrics of exposure (12-h average H_2_S per 1 ppb, 12-h PM_2.5-10_ per 10 ppb, 12-h average PM_10_ per 10 ppb, and 12-h average semivolatile PM_10_ per 10 ppb), and two highly correlated subjective measures of exposure (average 12-hour odor and twice-daily odor). The regression coefficients for eye irritation and measures of exposure were often positive (i.e., increased exposure was associated with a higher likelihood of eye-related symptoms). The exception to the positive association was the association between itching eyes and PM_2.5-10_ per 10 ppb (β = -0.08, 95% CI -0.43, 0.27).

The experimental study (Schiffman et al. 2005) reported p-values of 0.004 and 0.07 for the associations between differences in eye irritation for the exposed and unexposed groups. The p-value of 0.07 was for the same group measured at three times. The authors explained that "The p-value for time 3 is based on a test of whether the (time 2 – time 1) group differences persist at time 3. The time 3 test was performed only when group differences on (time 2 – time 1) were statistically significant." The results were not included in the figures because only p-values were provided. The risk of bias for this study was serious or critical, depending on the outcome.

**Gastrointestinal outcomes.** Gastrointestinal outcomes were assessed in only one publication (Schinasi et al. 2011). The associations with the exposures were reported as regression coefficients (Figure S7). The outcomes were self-assessed every 12 hours by each participant and represented the presence or absence of nausea, diarrhea, and poor appetite over the past 12 hours. Overall, there was no consistent direction of association between outcome and exposure. Only two effect sizes had intervals that did not include zero: poor appetite was positively associated with 12 h community-level average PM_10_ per 10 g/m^3^ (β = 0.51, 95% CI 0.12, 0.90), and nausea was negatively associated with 12 h community-level PM_2.5-10_ per 10 µg/m^3^ (β = -1.43, 95% CI -2.82, -0.04). There was an overall serious risk of bias associated with these objectively measured exposures. The associations with the subjectively measured exposures (hog odor) had an overall critical risk of bias due to the concerns previously discussed (relating to the CHEIHO study).

**Stress and mood outcomes.** Stress outcomes were assessed by Wing et al. (2013) and Avery et al. (2004), who reported the associations as regression coefficients (Figure S8), and by Horton et al. (2009), who reported the associations as odds ratios. Horton et al. (2009) reported a consistently positive association (point estimate and interval) between the exposure and the participants’ self-assessed levels of stress/annoyance, with one exception: stress or annoyance in the participants was not associated with neighborhood levels of PM_10_ (OR = 1.00, 95% CI 0.99, 1.01). Overall risk of bias for these associations was either serious (where the exposure was neighborhood levels of various pollutants) or critical, where the exposure was participant-assessed levels of odor, due to the issues with the CHEIHO study. These three studies were included in CHEIHO study.

Wing et al. (2013) used blood pressure as a measure of stress. Avery et al. (2004) assessed stress by measuring secretory immunoglobulin A in the saliva of study participants. Avery et al. (2004) found a consistent negative association between the log salivary IgA and participant-assessed hog odor outside their residences; however, the CIs included effect measures indicative of protective factors and risk factors (Figure S8). Wing et al. (2013) found that the effect measures for the associations between blood pressure and exposures were generally positive, with a few exceptions. Examples of associations include: neighborhood-level hydrogen sulfide and diastolic blood pressure in men (β = 0.48; 95% CI 0.11, 0.85), neighborhood-level hydrogen sulfide and systolic blood pressure in people with a score < 52 on the John Henryism Active Coping scale (β = 0.36, 95% CI 0.09, 0.63). Because the Wing et al. (2013) and Avery et al. (2004) studies were part of the CHEIHO study, the concerns about bias in that study also apply. Overall, the risk of bias was serious for both of these studies (Figure S8).

**Other non-infectious health outcomes.** Associations for the "other" outcome category reported as odds ratios are presented in Figure S7. The Schinasi et al. (2011) outcomes comprised fever, backache, and aching joints, and no consistent direction of association of these outcomes with the exposures was observed. Among the 21 results, three effect measures were associated with intervals that did not include both positive and negative effects. Aching joints was negatively associated with community-level 12 h average semivolatile PM_10_ per 10 µg/m^3^ (β = -0.93, 95% CI -1.85, -0.01) and positively associated with community-level PM_2.5-10_ per 10 µg/m^3^ (β = 0.30, 95% CI 0.01, 0.59). Backache was positively associated with community-level 12 h PM_2.5_ per 10 µg/m^3^ (β = 0.61, 95% CI 0.12, 1.10). The data were from the CHEIHO study. The overall risk of bias for associations with objectively assessed exposures was serious; it was critical for the subjectively assessed exposures (self-assessed hog odor outside the home). In this case, lack of consistency between associations was likely because outcomes that did not fit into the previously named outcome categories were placed into the “other” category. It is likely that there was variability between the underlying mechanisms associated with outcomes in the "other" category.

Additional effect measures (prevalence ratios) reported for the Lower Respiratory Outcome are reported in Figure S9. These are discussed in the main manuscript.

References

Avery RC, Wing S, Marshall SW, Schiffman SS. 2004. Odor from industrial hog farming operations and mucosal immune function in neighbors. Arch Environ Health 59:101-108.

Bullers S. 2005. Environmental Stressors, Perceived Control, and Health: The Case of Residents Near Large-Scale Hog Farms in Eastern North Carolina. Hum Ecol 33:1-16.

Horton RA, Wing S, Marshall SW, Brownley KA. 2009. Malodor as a trigger of stress and negative mood in neighbors of industrial hog operations. Am J Public Health 99 Suppl 3:S610-615.

Schiffman SS, Miller EA, Suggs MS, Graham BG. 1995. The effect of environmental odors emanating from commercial swine operations on the mood of nearby residents. Brain Res Bull 37:369-375.

Schiffman SS, Studwell CE, Landerman LR, Berman K, Sundy JS. 2005. Symptomatic effects of exposure to diluted air sampled from a swine confinement atmosphere on healthy human subjects. Environ Health Perspect 113:567-576.

Schinasi L, Horton RA, Guidry VT, Wing S, Marshall SW, Morland KB. 2011. Air pollution, lung function, and physical symptoms in communities near concentrated Swine feeding operations. Epidemiology 22:208-215.

Wing S, Horton RA, Rose KM. 2013. Air pollution from industrial swine operations and blood pressure of neighboring residents. Environ Health Perspect 121:92-96.

# Table S1. Search strategy used to identify articles on animal feeding operations and community health in Ovid MEDLINE® and MEDLINE® In-Process

| Search line | Search string |
| --- | --- |
| 1 | Animal Husbandry/ |
| 2 | Housing, Animal/ or Animal Feed/ |
| 3 | ((animal$1 or bovine or cow or cows or cattle or beef or pig or pigs or piglet$ or pork or swine or porcine or hog or hogs or finisher$ or sheep or murine or lamb or lambs or poultry or chicken$ or hen or hens or broiler$ or turkey$ or livestock or live stock or intensiv$ or industrial$ or confined or confinement or concentrated or large-scale or high density) adj4 (feed$ operation$ or feed$ facilit$)).ti,ab. |
| 4 | (cafo or cafos or afo or afos).ti,ab. |
| 5 | (feed lot$1 or feedlot$ or feedyard$ or feed yard$).ti,ab. |
| 6 | ((animal$1 or bovine or cow or cows or cattle or beef or pig or pigs or piglet$ or pork or swine or porcine or hog or hogs or finisher$ or sheep or murine or lamb or lambs or poultry or chicken$ or hen or hens or broiler$ or turkey$ or livestock or live stock) adj (density or operation$ or facility or facilities or confined or confinement)).ti,ab. |
| 7 | ((confined or confinement) adj3 (feed or feeding)).ti,ab. |
| 8 | ((intensive or intensively or intensity or large-scale or industrial or high-density) adj3 (farm or farms or farming or livestock or live stock)).ti,ab. |
| 9 | ((animal production or livestock production or live stock production) adj (operation$ or facility or facilities)).ti,ab. ( |
| 10 | or/1-9 |
| 11 | Environmental Health/ |
| 12 | environmental exposure/ or inhalation exposure/ |
| 13 | environmental pollutants/ or exp air pollutants/ or water pollutants/ |
| 14 | Environmental Illness/ |
| 15 | Environmental Monitoring/ |
| 16 | (public health$ or environmental health$ or environmental medicine or community health$).ti,ab,jn,jw. |
| 17 | ((public or community or communities or resident$ or residence$1 or living or neighbor$ or neighbour$ or family or families or local$1 or population$1 or populace or school$1 or preschool$ or highschool$ or nursery or nurseries or playgroup$ or play group$ or kindergarten$ or inhabitant$ or household$ or house hold$ or town$ or village$ or city or cities or settlement$) adj5 (proximity or vicinity or location$1 or located or nearby or near or close or closely)).ti,ab. |
| 18 | ((community or communities or resident$ or residence$1 or neighbor$ or neighbour$ or family or families or local$1 or populace$1 or school$1 or preschool$ or highschool$ or nursery or nurseries or playgroup$ or play group$ or kindergarten$ or inhabitant$ or household$ or house hold$ or town$ or village$ or city or cities or settlement$) adj5 (health or disease$1 or sickness$ or illness$ or infect$ or impact$ or effect$1 or exposure$1 or expose$1 or outcome$1 or symptom$1 or risk$1)).ti,ab. |
| 19 | or/11-18 |
| 20 | 10 and 19 |
| 21 | exp animals/ not humans/ |
| 22 | (news or editorial or letter).pt. |
| 23 | foot ortho$.ti,ab. |
| 24 | 20 not (21 or 22 or 23) |
| 25 | remove duplicates from 24 |

# Table S2. Characteristics of studies included in a systematic review of the associations between proximity to animal feeding operations and the health of individuals in nearby communities

| Study | Source | Exposure variable(s) | What was the size of the animal population under study? | Community studied |
| --- | --- | --- | --- | --- |
| Feingold et al. 2012 | As for study | Municipal-level pig density (# per ha) AND Municipal-level cow density AND Municipal-level veal calf density | NR | Cases were defined as livestock-associated nasal MRSA carriers diagnosed in the Netherlands between 2003 and September 2005, identified as "the first in a cluster of persons who tested positive for LA-MRSA from a given reference laboratory." Controls were index patients matched to the cases from the same laboratory testing positive for a typeable strain of MRSA. |
| Schiffman et al. 2005 | As for study | Subjects were exposed to either cleaned air (control) or diluted swine air (exposed) made by drawing emissions from an adjacent swine building and diluting it with cleaned air. | Not applicable (laboratory study) | Subjects were healthy adult volunteers who responded to advertisements posted in workplaces throughout the Research Triangle community of North Carolina (Durham, Chapel Hill and Raleigh). |
| The Netherlands study | Smit et al. 2014 | Presence of one or more farms within 500 m and 1000 m from the home address AND Total number of farms within 500 m and 1000 m AND  Distance to nearest farm AND The presence of a specific type of animal farm (swine, poultry, cattle, goat, sheep or mink) within 500 m and 1000 m AND PM_10_ emission from all farms within 500 m and 1000 m from the home address | Assessed indirectly based on provincial environmental licenses for keeping livestock: The median (IQR) of permitted animals per farm in the study area was 1242 (591–2441) pigs (n=3383 farms), 29 400 (11 800–72 000) chickens or other poultry (n=1005 farms), 122 (60–196) cows (n=4298 farms), 1309 (480–1726) goats (n=77 farms), 115 (80–200) sheep (n=230 farms) and 4000 (2800–6000) mink (n=111 farms) | Neighbouring residents of animal farms in the Dutch provinces of Noord-Brabant and Limburg, a highly populated area in the south of the Netherlands with a high density of farm animals. |
|  | Smit et al. 2012 | Presence of farm animals within 1 km of patient by species AND Number of goats within 5 km | In total there were 180 registered goat farms in this area with an average (permitted) number of 1307 goats (SD 1.195). | Patients registered in "general practices outside the larger cities in the eastern part of the province of Noord-Brabant and the northern part of the province of Limburg, a region with a high density of farm animals" |
| Lower Saxony Lung Study | Schulze et al. 2011 | Interpolated ammonia exposure | NR | German citizens living in one rural town with a high density of intensive livestock production in Lower Saxony. |
|  | Radon et al. 2007 | Level of odor annoyance AND Number of animal houses within 500 m. | NR | German citizens living in one of four rural towns in Lower Saxony, northwestern Germany, with a high density of animal feeding operations. |
|  | Radon et al. 2005 | Level of odor annoyance AND Number of animal stalls within 500 m of the subject's home. | NR | "German citizens living in four communities with a high animal-holding concentration in the rural district of Vechta and Cloppenburg"  **Article was translated from German into English.** |
| AABEL Study | Hoopmann et al. 2006 | Endotoxin at the subject's home, originating from animal stalls | A total of about 12,000 registered animal stalls (poultry, cows, pigs) | 5-6 year old children during the medical examination of school children upon their enrollment, from region of Lower Saxony with intensive agriculture (counties of Cloppenburg, Emsland, Oldenburg and Vechta)  **Article was translated from German into English.** |
| Schinasi et al. 2014 | As for study | Permitted swine per square mile of residential block group AND Permitted farrowing swine per square mile of residential block group AND Permitted non-farrowing swine per square mile of residential block group AND Distance of home to a CAFO AND Presence of farm odor at subject's residence | NR | Patients admitted to the Vidant Medical Center in eastern North Carolina who were tested for nasal carriage of MRSA using a PCR test. |
| Mirabelli et al. 2006 | As for study | Miles to nearest swine CAFO AND Number of hog pounds (in millions) within 3 miles of school AND Livestock odor outside + inside school | NR | Students (12-14 years old) who participated in the North Carolina School Asthma Survey |
| Bullers 2005 | As for study | Proximity of residence to CAFOs. | NR | Experimental group interviews were conducted in 1999 among residents of a rural area in the coastal plain of North Carolina. This is a sparsely populated rural agricultural county, which is the second largest hog-producing county in the USA. Virtually all of the family-run hog farms in this area have been replaced by industrial hog farm operations. The control group interviews were conducted among residents of another predominantly rural coastal plain county. Although County statistics of the control county show higher socioeconomic status and more urbanization, respondents were recruited from the western part of the county, which is very comparable to the experimental group county (with the exception of the industrial hog farm presence). |
| Schiffman et al. 1995 | As for study | Proximity of residence to hog operations. | NR | Residents of North Carolina; controls and exposed were matched by age, race, gender, years of education, and number of chronic illnesses experienced. |
| CHEIHO Study | Avery et al. 2004 | Intensity of perceived odor at home | NR but investigators stated that "The permitted number of animals in each operation ranged from 1,000 to 12,000" | Non-smokers, recruited in conjunction with local community organizations, living within 2.4 km of an intensive hog operation with at least one neighbor within 0.4 km of their home who was also willing to participate. |
|  | Schinasi et al. 2011 | Strength of odor at the subject's home AND 1-h and 12-h average hydrogen sulfide per 1 ppb, 1-h and 12-h average PM_10_ per 10 µg/m^3^ AND 1-h and 12-h average semi-volatile PM_10_ per µg/m^3^ | "There was a median of 9 hog operations within 2 miles of participating communities, and the median number of hogs within that radius was approximately 42,000" | Residents of 16 eastern North Carolina communities who were non-smoking volunteers residing within 1.5 miles of at least one hog operation, recruited through community-based organizations. |
|  | Horton et al. 2009 | Neighborhood hydrogen sulfide AND Neighborhood PM_10_ (µg/m^3^) AND Neighborhood semi-volatile PM_10_ (µg/m^3^) AND Strength of odor at the home | NR | Residents of 16 eastern North Carolina rural communities who were non-smoking volunteers residing within 1.5 miles of at least one hog operation, recruited through community-based organizations. |
|  | Wing et al. 2013 | Strength of hog odor at the home AND Neighborhood hydrogen sulfide (ppb) AND Neighborhood PM_10_ (µg/m^3^) AND Neighborhood semi-volatile PM_10_ (µg/m^3^) | NR | Residents of 16 eastern North Carolina rural communities who were non-smoking volunteers residing within 1.5 miles of at least one hog operation, recruited through community-based organizations. |

# Table S3. P-values reported for associations assessed by Schiffman et al. 2005.

| Health Outcome Evaluated | Effect measure tested | p-value reported |
| --- | --- | --- |
| Nasal lavage - Percent epithelial cells | Beta-regression coefficient | 0.02 |
| Nasal lavage - Percent lymphocytic cells | Beta-regression coefficient | 0.08 |
| Nasal lavage - IL-8 (pg/mL) | Beta-regression coefficient | 0.11 |
| Nasal lavage - Absolute epithelial cells | Beta-regression coefficient | 0.15 |
| Nasal lavage - Percent PMNs | Beta-regression coefficient | 0.22 |
| Nasal lavage - Absolute PMNs | Beta-regression coefficient | 0.27 |
| Temperature | Beta-regression coefficient | 0.27 |
| Diastolic blood pressure | Beta-regression coefficient | 0.29 |
| Digit span score | Beta-regression coefficient | 0.35 |
| Nasal lavage - IL-1e(pg/mL) | Beta-regression coefficient | 0.38 |
| Mood scores (POMS) - Anxiety | Beta-regression coefficient | 0.39 |
| Mood scores (POMS) - Depression | Beta-regression coefficient | 0.45 |
| Blood pressure ratio (systolic to diastolic) | Beta-regression coefficient | 0.52 |
| Mood scores (POMS) - Fatigue | Beta-regression coefficient | 0.52 |
| Mood scores (POMS) - Vigor | Beta-regression coefficient | 0.52 |
| Mood scores (POMS) - Total mood | Beta-regression coefficient | 0.55 |
| Respiratory rate | Beta-regression coefficient | 0.57 |
| Salivary IgA (ug/mL) | Beta-regression coefficient | 0.57 |
| Systolic blood pressure | Beta-regression coefficient | 0.7 |
| Nasal lavage - Cell counts | Beta-regression coefficient | 0.76 |
| Heart rate | Beta-regression coefficient | 0.78 |
| Nasal lavage - Absolute lymphocytic cells | Beta-regression coefficient | 0.78 |
| Percent change FVC | Beta-regression coefficient | 0.8 |
| Mood scores (POMS) - Confusion | Beta-regression coefficient | 0.83 |
| Percent change FEF 25-75% | Beta-regression coefficient | 0.88 |
| Mood scores (POMS) - Anger | Beta-regression coefficient | 0.97 |
| Percent change FEV1 | Beta-regression coefficient | 0.98 |
| Self-reported symptoms - Headache | Odds ratio | 0.001 |
| Self-reported symptoms - Eyes irritated | Odds ratio | 0.004 |
| Self-reported symptoms - Headache (Group differences at Time 3) | Odds ratio | 0.01 |
| Self-reported symptoms - Nausea | Odds ratio | 0.014 |
| Self-reported symptoms - Eyes irritated (Group differences at Time 3) | Odds ratio | 0.07 |
| Self-reported - Itchy throat | Odds ratio | 0.12 |
| Self-reported symptoms - Nasal secretion | Odds ratio | 0.22 |
| Self-reported symptoms - Sore throat | Odds ratio | 0.27 |
| Self-reported symptoms - Nasal irritation | Odds ratio | 0.34 |
| Self-reported symptoms - Nausea (Group differences at Time 3) | Odds ratio | 0.57 |
| Self-reported symptoms - Cough | Odds ratio | 0.66 |
| Self-reported symptoms - Nasal congestion | Odds ratio | 0.76 |

# Figure S1. Neurological and psychological symptoms, and stress outcomes for which the effect size was reported as an odds ratio.

# Figure S2. Neurological symptoms for which the effect size was reported as a regression coefficient.

# Figure S3. Psychological outcomes for which the effect size was reported as a point estimate of the mean difference

# Figure S4. Psychological outcomes for which the effect size was reported as a point estimate).

# Figure S5. Psychological outcomes for which the effect size was reported as a regression coefficient).

# Figure S6. Dermatologic, otologic, and optical outcomes for which the effect size was reported as a regression coefficient*.*

# Figure S7. Gastrointestinal and "Other" outcomes for which the effect size was reported as a regression coefficient (β)*.*

# Figure S8. Stress outcomes for which the effect size was reported as a regression coefficient(β).

**Figure S9. Lower respiratory outcomes for which the effect size was reported as a prevalence ratio**
