# Supplementary material for: Updated systematic review: associations between proximity to animal feeding operations and health of individuals in nearby communities
Source: Syst Rev. 2017 Apr 18;6:86. doi: 10.1186/s13643-017-0465-z (PMC5395850; doi:10.1186/s13643-017-0465-z)
Supplement: Additional file 1: — Discussion about other outcomes included in the systematic review. Excel Spreadsheet with extracted data from review. Data extraction forms, risk of bias forms and search startergies used for review. Figure S1. Neurological and psychological symptoms and stress outcomes for which the effect size was reported as an odds ratio. Figure S2. Neurological symptoms for which the effect size was reported as a regression coefficient. Figure S3. Psychological outcomes for which the effect size was reported as a point estimate of the mean difference. Figure S4. Psychological outcomes for which the effect size was reported as a point estimate. Figure S5. Psychological outcomes for which the effect size was reported as a regression coefficient. Figure S6. Dermatologic, otologic, and optical outcomes for which the effect size was reported as a regression coefficient. Figure S7. Gastrointestinal and “Other” outcomes for which the effect size was reported as a regression coefficient (β). Figure S8. Stress outcomes for which the effect size was reported as a regression coefficient (β). Figure S9. Lower respiratory outcomes for which the effect size was reported as a prevalence ratio. (ZIP 1.40 mb) [file 13643_2017_465_MOESM1_ESM.zip › Additional Supporting Material for CAFO review- ISU_DDR1.docx]

Additional Materials To Support : **Updated systematic review of the association between proximity to animal feeding operations and the health of individuals in nearby communities**

Annette M. O'Connor ^1^, Brent W. Auvermann ^2^, Rungano S. Dzikamunhenga ^1^, Julie M. Glanville ^3^, Julian P.T. Higgins ^4^, Shelley P. Kirychuk ^5^, Jan M. Sargeant ^6^, Sarah C. Totton,^7^ Hannah Wood ^5^, and Susanna G. Von Essen ^8^

SM 1: Data extraction form used to collect study-level information in a systematic review of the association between proximity to animal feeding operations and the health of individuals in nearby communities. 3

SM 2: Data extraction form used to collect outcome-level information in a systematic review of the association between proximity to animal feeding operations and the health of individuals in nearby communities. 13

SM 3: Risk-of-bias tool for non-randomized studies of exposures (case-control and cross-sectional studies) for a systematic review of the association between proximity to animal feeding operations and the health of individuals in nearby communities (modified from a preliminary version of the ROBINS-I tool. 35

SM 4: Risk-of-bias tool for non-randomized studies of exposures (cohort studies) for a systematic review of the association between proximity to animal feeding operations and the health of individuals in nearby communities (modified from a preliminary unpublished version of the ROBINS-I tool). 59

SM 5: Risk-of-bias tool used for non-randomized experimental studies in a systematic review of the association between proximity to animal feeding operations and the health of individuals in nearby communities (modified from the Cochrane Risk-of-Bias Tool, Table 8.5a in Higgins and Green 2011). 85

SM 6: Full search strategy and results for a database search of Ovid MEDLINE® In-Process & Other Non-Indexed Citations and Ovid MEDLINE® (dates covered: 1946 to Present; date of search 15 September 2014) for a systematic review of the association between proximity to animal feeding operations and the health of individuals in nearby communities. 92

SM 7: Full search strategy and results for a database search of Science Citation Index (Web of Knowledge; dates covered 1900 to 13 September 2014; search date 15 September 2014) for a systematic review of the association between proximity to animal feeding operations and the health of individuals in nearby communities. 97

SM 8: Full search strategy and results for a database search of CAB Abstracts (Web of Knowledge) (dates covered 1910 to present; date searched 3 October 2014) for a systematic review of the association between proximity to animal feeding operations and the health of individuals in nearby communities. 101

SM 9: Results of multiple database searches for a systematic review of the association between proximity to animal feeding operations and the health of individuals in nearby communities 105

SM 1: Data extraction form used to collect study-level information in a systematic review of the association between proximity to animal feeding operations and the health of individuals in nearby communities.

| Study-Level Information Form | | |
| --- | --- | --- |
| Question | Style | Response |
| Q1. What is the study design? | Radio | Observational: cross-sectional  Observational: cohort  Observational: case-control  Experimental |
| Q2. What is the month, year the study was started? | Text |  |
| Q3. What is the month, year the study ended? | Text |  |
| Q4. What is the location of the study population (country)? | Radio | The Netherlands  USA  Germany  Option for reviewer to add another country as needed. |
| Q5. What is the study location area (i.e. region within country)? | Checkbox | Eastern part of the province of Noord-Brabant  Northern part of the province of Limburg  Eastern North Carolina  Lower Saxony, northwestern Germany  North Carolina  Noord-Brabant and Limburg  A rural area in the coastal plain of North Carolina  Experimental laboratory  Regions of Cloppenburg, Emsland, Oldenburg and Vechta  Option for reviewer to add new location as needed |
| Q6. What is the size of the human population in the source population? (maximum no. of ppl data was collected on)? | Text |  |
| Q7. What is the size of the study population? | Text |  |
| Q8. What is the age of the study population? | Text |  |
| Q9. What is the sex of the study population? | Radio | Males  Females  Both males and females  Not specifically reported |
| Q10. What is the socioeconomic status of the study population? | Checkbox | Not specifically reported  "Most residents are in the low- to middle-income categories"  Option for reviewer to add another answer as needed |
| Q11. What is the size of the animal population under study? | Text |  |
| Q12. What is the unit of concern for the outcome? | Radio | The individual  Option for reviewer to add another answer as needed |
| Q13. What are the "animal'' variables used? | Checkbox | Presence of farm animals within 1 km of patient by species  Number of goats within 5 km  Within 1.5 miles of at least one hog operation  Interpolated ammonia exposure  Presence of one or more farms within 500 m and 1000 m from the home address  Total number of farms within 500 m and 1000 m  Distance to nearest farm  The presence of a specific type of animal farm (swine, poultry, cattle, goat, sheep or mink) within 500 m and 1000 m  PM_10_ emission from all farms within 500 m and 1000 m from the home address  Hog operations In North Carolina  Exposure to intensive agriculture  Exposure to hogs only  Within 2 mile radius of one of two cattle farms  Dairy farms  Exposure to dairy farms  Exposure to diluted swine air  Permitted swine per square mile of block group  Live within one mile of a CAFO  living within 2.4 km (1.5 mi) of an intensive hog operation  Lagrange propagation model was developed to estimate the barn-related bioaerosol emissions (endotoxin, fungal concentration, total dust and total bacterial concentration) at the residence of each individual test subject  Distance of school from nearest swine CAFO  Hog pounds (in millions) within 3 miles of school  Livestock odor inside and/or outside school  Option for reviewer to add a new answer as needed |
| Q14. Describe the community being studied. | Checkbox | Being within 5km of farm keeping goats as the main type of animal, or other livestock farms with at least 50 goats  Participants spent 10 minutes outdoors at preselected morning and evening times approximately 12 hours apart. While outside, they rated, on a scale of 0 (none) to 8 (very strong), the strength of the hog odor they recalled having smelled during each of the 12 preceding hours. Participants then returned indoors and rated hog odor present during the 10 minutes outside on the same 9-point scale  Exposure to H_2_S, semi-volatile PM_10_, PM_10_, PM_2.5-10_, PM_2.5_ and endotoxin  Living in a residence whose interpolated ammonium exposure was in the 3 highest quartiles (≥19.71 µg/m^3^)  Living within 500 m of at least one animal farm  Living near a hog operation  Residence within 1 mile of a swine or poultry CAFO, swine densities(total, farrowing, and non-farrowing) in the block group of residence, ability to ever smell odor from an animal farm when at home  Cases were defined as MRSA nasal carriers, based on a positive result from the rapid PCR screen that was administered at hospital admission.  Neighbouring residents of animal farms in the Dutch provinces of Noord-Brabant and Limburg, a highly populated area in the south of the Netherlands with a high density of farm animals.  Eligible participants in the CHEIHO study were non-smoking adults who lived within 1.5 miles of at least 1 industrial hog operation and were willing to collect data twice daily for approximately 2 weeks.  Resident of 4 rural towns in Germany  5-6 year old children during the medical examination of school children upon their enrollment, from region of Lower Saxony with intensive agriculture (counties of Cloppenburg, Emsland, Oldenburg and Vechta)  Experimental group interviews were conducted in 1999 among residents of a rural area in the coastal plain of North Carolina. This is a sparsely populated rural agricultural county with a relatively stable population of just under 50,000 residents. Most residents are in the low- to middle-income income categories. This county is the second largest hog-producing county in the US. Virtually all of the family-run hog farms in this area have been replaced by industrial hog farm operations. The control group interviews were conducted among residents of another predominantly rural coastal plain county. Although County statistics of the control county show higher SES and more urbanization, respondents were recruited from the western part of the county, which is very comparable to the experimental group county (with the exception of the industrial hog farm presence).  Within a 2-mile radius around the dairy farms within a single census block  Students (12-14 years old) who participated in the North Carolina School Asthma Survey  Option for reviewer to add new answer as needed |
| Q15. What is the definition of an ‘‘unexposed'' person or community? | Checkbox | Being > 5 km of farm keeping goats as the main type of animal, or other livestock farms with at least 50 goats  Each participant served as her or his own control  Living in a residence whose interpolated ammonia exposure was in the first quartile (<19.71 µg/m^3^)  Living > 500 m but less than 1000m of at least one animal farm  Not described (assumed to be "not" living near a hog operation)  Controls were non-MRSA carrying patients based on a negative PCR screen. One control was matched to each case based on age (±5 y) and gender. When more than one patient was an eligible match for a case, a random number generator was used to select the potential control.  Children of parents from a region of intensive agriculture in Lower Saxony, including the regions of Cloppenburg, Emsland, Oldenburg and Vechta  Option for reviewer to add another answer as needed. |
| Q16. What statistical approach is used to assess the association? (check all that apply) | Checkbox | Univariate approaches to categorical data : chi-square tests  Multivariable approaches to categorical data with fixed and/or random effects: logistic regression/ generalized linear model with logit link  Univariable approaches to continuous outcomes (t test, ANOVA)  Multivariable approaches to continuous outcomes with fixed and/or random effects  Other  Multivariable approaches to dichotomous data with no fixed or random effects: logistic regression  Multivariable approaches to continuous data with no fixed or random effects: linear regression  Option for reviewer to add another answer as needed |

SM 2: Data extraction form used to collect outcome-level information in a systematic review of the association between proximity to animal feeding operations and the health of individuals in nearby communities.

| Outcome-level Data Extraction Form | | |
| --- | --- | --- |
| Question | Style | Response |
| Q1. Specify the outcome variable. | Radio | Pneumonia  Other infectious disease  Eye irritation  Nasal irritation  Throat irritation  Skin irritation  Cough  Wheezing without a cold  Allergic rhinitis  Sensitization against ubiquitous allergens  Tiffeneau index (% of predicted)  Forced expiratory volume in the first second (FEV_1_) (% of predicted)  Asthma  Chronic obstructive pulmonary disease (COPD)  Tension  Depression based on POM (Profile of Mood States) score  Anger based on POM score  Vigor based on POM score  Fatigue based on POM score  Confusion based on POM score  TMD (total mood disturbance score)  Peak expiratory flow rate (PEF)  FEV_1_  Stressed or annoyed  Nervous or anxious  Gloomy, blue, or unhappy  Angry, grouchy, or bad-tempered  Confused or unable to concentrate  Change in activities in relation to average odor during the previous 12 h  Wheezing without cold  Physician-diagnosed asthma  Specific IgE to common allergens > 0.35 IU/mL  Bronchial hyper-responsiveness to methacholine  FEV_1_ (% Predicted)  Average number of headache episodes  Average number of stuffy nose/sinuses  Average number of runny nose  Average number of burning nose/sinuses  Average number of sore throats  Average number of plugged/popping ears  Average number of scratchy throats  Average number of mucus/phlegm episodes  Average number of excessive coughing episodes  Average number of shortness of breath episodes  Average number of tightness in chest episodes  Average number of wheezing episodes  Average number of strange breathing sounds  Average number of heartburn episodes  Average number of nausea/vomiting episodes  Average number of no appetite episodes  Average number of diarrhea episodes  Average number of burning eyes episodes  Average number of tearing eyes episodes  Average number of dry/scaly skin  Average number of skin rash or irritation episodes  Average number of skin redness episodes  Average number of joint/muscle pain episodes  Average number of unexplainably tired episodes  Average number of blurred vision episodes  Average number of dizzy/faint episodes  Average number of hearing problems episodes  Average number of chest pain episodes  Average number of fever/chills episodes  Average number of fainted episodes  Average number of "can't open windows" episodes  Average number of "can't go outside" episodes  Systolic blood pressure (SBP)  Diastolic blood pressure (DBP)  SBP in age ≤ 53.7 y  SBP in age > 53.7 y  SBP in women  SBP in men  SBP in butanol threshold ≤ 40 ppm  SBP in butanol threshold > 40 ppm  SBP for John Henryism Active Coping (JHAC) score ≤ 52  SBP for JHAC score > 52  SBP for no BP meds  SBP for any BP meds  DBP for age ≤ 53.7 y  DBP for age > 53.7 y  DBP for women  DBP for men  DBP for butanol threshold ≤ 40 ppm  DBP for butanol threshold > 40 ppm  DBP for JHAC score ≤ 52  DBP for JHAC score > 52  DBP for no meds  DBP for any BP meds  Heart rate  Respiratory rate  Temperature  Blood pressure ratio (systolic to diastolic)  Percent change FEV_1_  Percent change FVC (forced vital capacity)  Percent change FEF 25–75% (averaged forced expiratory flow between the full expiration of 25 and 75% of the total FVC)  Salivary IgA (µg/mL)  Mood scores (POMS) – Anxiety  Mood scores (POMS) - Total mood  Digit span score  Nasal lavage - IL-8 (pg/mL)  Nasal lavage - IL-1β (pg/mL)  Nasal lavage - Cell counts  Nasal lavage - Percent epithelial cells  Nasal lavage - Percent lymphocytic cells  Nasal lavage - Percent polymorphonuclear cells (PMNs)  Nasal lavage - absolute epithelial cells  Nasal lavage - absolute lymphocytic cells  Nasal lavage - absolute PMNs  Self-reported symptoms – headache  Self-reported symptoms - sore throat  Self-reported - itchy throat  Self-reported symptoms - eyes irritated  Self-reported symptoms - nasal congestion  Self-reported symptoms - nasal secretion  Self-reported symptoms - nasal irritation  Self-reported symptoms – cough  Self-reported symptoms – nausea  Psychological distress  Non-cold-related rhonchal breathing sounds  Asthmatic pathology  Tiffenau Index median deviation  Runny nose  Mucus or phlegm  Sore throat  Wheezing  Difficulty breathing  Chest tightness  Burning eyes  Itching eyes  Nausea  Diarrhea  Poor appetite  Headache  Dizziness  Aching joints  Difficulty hearing  Fever  Backache  Nasal MRSA carriage  Log salivary IgA concentration (ug/ml)  Log salivary IgA secretion rate (ug/min)  Current wheeze children with self-reported allergies  Current wheeze children without self-reported allergies  Current wheeze in all children  Current wheeze without physician diagnosis in children with self-reported allergies  Severe wheeze in children with self-reported allergies  Physician-diagnosed asthma in children with self-reported allergies  Current wheeze without physician diagnosis in children with no self-reported allergies  Severe wheeze in children with no self-reported allergies  Physician-diagnosed asthma in children with no self-reported allergies  Current wheeze in children with no self-reported allergies  Frequent severe wheeze in children with no self-reported allergies  Current wheeze without physician diagnosis in all children  Severe wheeze in all children  Frequent severe wheeze in all children  Physician-diagnosed asthma in all children  Frequent severe wheeze in children with self-reported allergies  Asthma-related physician visit, emergency visit and/or hospitalization in the past year, self-reported allergies  Asthma medication use in past year, self-reported allergies  Activity limitations in past year as a result of asthma symptoms  Missed school in past year as a result of asthma symptoms  Asthma-related physician visit, emergency visit, and/or hospitalization in past year, no self-reported allergies  Asthma medication use in past year, no self-reported allergies  Asthma-related physician visit, emergency visit, and/or hospitalization in past year, all children  Asthma medication use in past year, all children  Allergic asthma  Non-allergic asthma  IgE  Option for reviewer to add another answer if needed |
| Q2. What is the community health/animal exposure measure? | Radio | Number of goats within 5 km  Presence of farm within 1 km  Twice-daily odor  1-h average H_2_S per 1 ppb  1-h average H_2_S PM_10_ per 10ug/m^3^  1-h average semi-volatile PM_10_ per 10g/m^3^  Interpolated ammonia exposure ≥19.71 µg/m  PM_10_ emission from farms within 500 m  Distance to nearest farm  One or more farms within 500 m  Number of farms within 500 m  Presence of farm animals within 500 m  Could smell hog odor  12-h average odor  12-h average H_2_S per 1 ppb  12-h average PM_10_ per 10 µg/m^3^  12-h average semi-volatile PM_10_ per 10 µg/m^3^  Hydrogen sulfide (ppb)  PM_10_ (ug/m^3^)  Semi-volatile PM_10_ (ug/m^3^)  Twice daily odor rating (0-8)  12-h average odor  Level of odor annoyance  No. of animal houses within 500 m  Exposure to cattle  Odor (0-8)  H_2_S ppb  PM_10_ (µg/m^3^)  Semi-volatile PM_10_ (µg/m^3^)  Diluted aerial emissions from swine house  Nearby residence  Number of animal stalls within a radius of 500 m of the home  Log of the endotoxin  Odor nuisance  Permitted swine per square mile  Permitted farrowing swine per square mile of block group  Permitted non-farrowing swine per square mile of block group  Live within 1 mile of a CAFO  Ever smell odor from a farm with animals when at home  Hog odor (continuous variable recoded as 1-3, 4,5,6,7,8,9)  Hog odor coded as 1= odor rating 4-9; 0 = odor rating 1-3)  Miles to nearest swine CAFO  Hog pounds (in millions) within 3 miles of school  Exposure category (low, medium, high; based on tertiles of the distribution of values among schools with > 1 swine CAFOs within 3 miles of the school)  Livestock odor  PR (95% CI) for < 3 vs >3 miles from nearest swine CAFO  Livestock odor reported outside or inside school building vs no reported odor  12-h PM_2.5-10_ per 10 µg/m^3^  12-h PM_2.5_ per 10 µg/m^3^  12-h endotoxin per 10 µg/m^3^  Option for reviewer to add another category |
| Exposure subcategory | Radio | 0-2250 (number of goats within 5 km)  2251-7250  7251-17,190  17,191-20,960  Swine (presence of farm animals within 1 km)  Poultry  Cattle  Sheep  Mink  >640 m (reference) (distance to nearest farm)  440-640 m  280-440 m  50-280 m  Goat  Odor < 1  1 ≤ odor < 2  2 ≤ odor < 3  3 ≤ odor < 5  Odor ≥ 5  Not at all (level of odor annoyance)  Somewhat  Moderately  Strongly  ≤5  ≤10  ≤12  >12  0-5 (number of stalls)  6-10  11-12  Non-atopic parents  Atopic parents  None (odor nuisance)  A little  Considerable  Strong  0 (permitted farrowing swine per square mile of block group)  > 0-149  > 149  No (ever smell odors from a farm with animals when at home)  Yes  > 3 (miles to nearest swine CAFO)  < 3  2 to < 3  < 2  0.1 to < 2 (hog pounds in millions within 3 miles of school)  2.0 to <5.0  > 5.0  Low (exposure category)  Medium  High  Outside school only (livestock odor)  Outside AND inside <2 times/mo  Outside AND inside > 2 times/mo  Group differences at Time 3 (2 h after end of exposure)  Option for reviewer to add a new answer as needed |
| Type of effect measure | Radio | PR  OR  beta  p  Mean difference in continuous outcome  Option for reviewer to add a new answer as needed |
| Effect measure | Text | Enter the value of the reported effect measure. |
| Upper limit of 95% CI point estimate (calculate as needed effect size +/- 1.96*SE, then round to 2 decimal places, round 5 up. i.e., 2.345 will become 2.35, and 2.344 will become 2.34) | Text |  |
| Lower limit of 95% Ci point estimate | Text |  |
| List all of the covariates adjusted for (include in alphabetical order, separated by ","). If none - say none | Text |  |

SM 3: Risk-of-bias tool for non-randomized studies of exposures (case-control and cross-sectional studies) for a systematic review of the association between proximity to animal feeding operations and the health of individuals in nearby communities (modified from a preliminary version of the ROBINS-I tool.

| Risk of bias for non-randomized studies (case control/cross-sectional studies) | | |
| --- | --- | --- |
| Question | Style | Response |
| Q1. What is the study design? | Radio | Observational: Cross-sectional  Observational Case-control |
| Q2. Describe the exposure / outcome grouping. | Radio | Objective exposures / Objective outcomes  Objective exposures / Subjective outcomes  Subjective exposures / Subjective outcomes  Subjective exposures / Objective outcomes |
| Confounding Bias | | |
| Q3. Outcome confounding domains | Checkbox | Respiratory  Gastrointestinal  Mood  Eye  Dermatologic  Lifestyle  Neurologic  MRSA  Otologic  Other |
| Q4. List the critically important respiratory outcome confounding domains that the authors controlled for in this study. | Checkbox (only appears if "Respiratory" was checked for Q3) | Not reported  Family history of allergies or respiratory disease  Presence of wood-burning stove(s)  Exposure to tobacco smoke  Socioeconomic status (may be indicated by race, education, employment status)  Concurrent disease  Presence of cats in the house |
| Q5. List the critically important gastrointestinal outcome confounding domains that the authors controlled for in this study. | Checkbox (only appears if Gastrointestinal was checked for Q3) | Pre-existing disease |
| Q6. List the critically important mood outcome domains that the authors controlled for in this study. | Checkbox (only appears if Mood was checked for Q3) | Socioeconomic status  None of the above |
| Q7. List the critically important eye outcome domains that the authors controlled for in this study. | Checkbox (only appears of Eye was checked for Q3) | Pet ownership  Atopic parents  Factors associated with allergies |
| Q8. List the critically important lifestyle outcome domains that the authors controlled for in this study. | Checkbox (only appears if Lifestyle was checked for Q3) | The "Lifestyle" category was considered too broad to have any specific critical confounders. |
| Q9. List the critically important neurologic outcome domains that the authors controlled for in this study. | Checkbox (only appears if Neurologic was checked for Q3) | Socio-economic status  Pre-existing disease |
| Q10. List the critically important MRSA outcome domains that the authors controlled for in this study. | Checkbox (only appears if MRSA was checked for Q3) | Livestock ownership (horses, cattle)  None of the above |
| Q11. List the critically important Dermatologic outcome domains that the authors controlled for in this study. | Checkbox (only appears if Dermatologic was checked for Q3) | Ownership of pets  Atopic parents  Factors associated with allergies |
| Q12. List the critically important otologic outcome domains that the authors controlled for in this study. | Checkbox (only appears if Otologic was checked for Q3) | Some pesticide usage |
| Q13. List the critically important "Other" outcome domains that the authors controlled for in this study. | Checkbox (only appears if Other was checked for Q3) | The "Other" category was considered too broad to have any specific critical confounders. |
| Q14. Is confounding of the effect of exposure likely in this study?  If No or Probably No the study can be considered to be at low risk of bias due to confounding and no further signalling questions need be considered. | Radio | Yes  Probably Yes  Probably No  No  No information |
| Provide justification for your answer to Q14. | Text |  |
| Q15. Did the authors use an appropriate analysis method that adjusted for all of the critically important confounding domains?  NB: Keep in mind that based on the study, some confounders may overlap. | Radio | Yes  Probably Yes  Probably No  No  Not applicable  No information |
| Provide justification for your answer to Q15. | Text |  |
| Q16. If Y or PY to Q15 Were critical confounding domains that were adjusted for measured *validly* and *reliably* by the variables available in this study? | Radio | Yes  Probably Yes  Probably No  No  Not applicable  No information |
| Provide justification for your answer to Q16. | Text |  |
| Q17. What is the Confounding Bias for this study? (Please see p. 20, Sterne et al. 2014) | Radio | Low  Moderate  Serious  Critical |
| Provide justification for your answer to Q17. | Text |  |
| Q18. What is the predicted direction of bias due to confounding?  For guidance, please refer to: Mehio-Sibai et al. 2005. | Radio | Towards the null  Away from the null  Unpredictable |
| Provide justification for your answer to Q18. | Text |  |
| Selection Bias | | |
| Q19. Were the controls/unexposed subjects sampled from the population that gave rise to the cases/diseased, or using another method that avoids selection bias? (Good studies would get a "Yes".)  Selection bias is an issue if there is differential enrollment of a group in one of the cells in the 2X2 table. Differential enrollment based on exposure only would not be an issue of selection bias, nor would it be an issue if the investigators differentially enrolled subjects based on disease status only. | Radio | Yes  Probably Yes  Probably No  No |
| Provide an explanation for your answer to Q19. | Text |  |
| Q20. What is the risk of Selection Bias in this study? (Refer to p. 25 in Sterne et al. 2014.)  NB: Low risk of bias means the study is comparable to a well-performed randomized trial. This is unlikely in an observational study. Moderate risk of bias means selection into the study may have been related to exposure and outcome status but the investigators used appropriate methods to adjust for selection bias. | Radio | Low  Moderate  Serious  Critical |
| Justify your answer to Q20. | Text |  |
| Q21. What is the predicted direction of bias due to selection of participants into the study? | Radio | Towards the null  Away from the null  Unpredictable |
| Please give an explanation of your answer to Q21. | Text |  |
| Bias in exposure measurement | | |
| Q22. Was the exposure status well defined? | Radio | Yes  No |
| Any additional comments for your answer to Q22. | Text |  |
| Q23. How accurately did the investigators measure the specified exposure(s)? | Radio | Relatively accurate  Inaccurate  No information |
| Provide justification for your answer to Q23. | Text |  |
| Q24. Was information on exposure status recorded at the time of exposure? | Radio | Yes  Probably Yes  Probably No  No  Can't tell |
| Justify your answer to Q24. | Text |  |
| Q25. Was information on exposure status unaffected by knowledge of the outcome or risk of the outcome?  (Good studies get a Yes.) | Radio | Yes  Probably Yes  Probably No  No  Not applicable  No information |
| Provide justification for your answer to Q25. | Text |  |
| Q26. What is the risk of bias due to measurement of exposure? (Refer to p. 27 in Sterne et al. 2014). | Radio | Low  Moderate  Serious  Critical  No information |
| Justify your answer to Q26. | Text |  |
| Q27. What is the predicted direction of bias due to measurement of exposures? | Radio | Towards the null  Away from the null  Unpredictable |
| Provide justification for your answer to Q27. | Text |  |
| Bias due to missing data | | |
| Q28. Was the outcome status reasonably complete for those in whom it was sought?  < 10% data missing constitutes reasonably complete | Radio | Yes  Probably Yes  Probably No  No  No information/numbers not reported |
| Provide justification for your answer to Q28. | Text |  |
| Q29. Were data on exposure status reasonably complete?  < 10% data missing constitutes reasonably complete | Radio | Yes  Probably Yes  Probably No  No  No information/numbers not reported |
| Justify your answer to Q29. | Text |  |
| Q30. Are data reasonably complete for other variables in the analysis?  < 10% data missing is reasonably complete | Radio | Yes  Probably Yes  Probably No  No  No information/numbers not reported |
| Provide justification for your answer to Q30. | Text |  |
| Q31. If you answered "No" to Q28, Q29 or Q30, were the proportion of participants and reasons for missing data similar across cases/diseased and controls/non-diseased? | Radio | Yes  Probably Yes  Probably No  No  No information  Can't tell |
| Provide an explanation for your answer to Q31. | Text |  |
| Q32. If you answered "No" to Q28, Q29 or Q30, were appropriate statistical methods used to account for missing data (i.e. do author's describe missing data imputation)? | Radio | Yes  Probably Yes  Probably No  No  Not applicable  No information |
| Provide an explanation for your answer to Q32. | Text |  |
| Q33. What is the risk of bias due to missing data? (refer to p. 36 in Sterne et al. 2014) | Radio | Low  Moderate  Serious  Critical  Unable to assess |
| Provide justification for your answer to Q33. | Text |  |
| Q34. What is the predicted direction of bias due to missing data?  Depends on what's missing, and most of the time you won't be able to tell the direction of bias. | Radio | Towards the null  Away from the null  Unpredictable |
| Provide justification for your answer to Q34. | Text |  |
| Bias in measurement of outcomes | | |
| Q35. Was the definition of case/disease (and control/non-diseased status, if applicable) based on objective criteria? | Radio | Yes  Probably Yes  Probably No  No |
| Provide an explanation for your answer to Q35. | Text |  |
| Q36. Was the definition of case/disease status (and control/non-disease status, if applicable) applied without knowledge of the exposure received? (This will likely be "no" for subjective outcomes and "yes" for objective outcomes.) | Radio | Yes  No  Can't tell |
| Provide an explanation for your answer to Q36. | Text |  |
| Q37. What is the risk of bias due to measurement of outcomes? (Refer to p.39 in Sterne et al. 2014.) | Radio | Low  Moderate  Serious  Critical  No information |
| Provide an explanation for your answer to Q37. | Text |  |
| Q38. What is the predicted direction of bias due to measurement of outcomes? | Radio | Towards the null  Away from the null  Unpredictable |
| Provide justification for your answer to Q38. | Text |  |
| Bias in selection of reported results | | |
| Q39. Is the reported effect estimate unlikely to be selected on the basis of the results due to multiple definitions of the exposure?  Answer "Yes" or "Probably Yes" if, for example, the variable was continuous but was treated as categorical. | Radio | Yes  Probably Yes  Probably No  No |
| Provide an explanation for your answer to Q39. | Text |  |
| Q40. Is the reported effect estimate unlikely to be selected on the basis of the results due to multiple analyses of the exposure-outcome relationship? | Radio | Yes  Probably Yes  Probably No  No  Can't tell |
| Provide an explanation for your answer to Q40. | Text |  |
| Q41. Is the reported effect estimate unlikely to be selected on the basis of results due to different subgroups? | Radio | Yes  Probably Yes  Probably No  No  Not applicable |
| Provide an explanation for your answer to Q41. | Text |  |
| Q42. What is the risk of bias due to selection of reported results? (Refer to p.43 in Sterne et al. 2014.) | Radio | Low  Moderate  Serious  Critical  Unable to assess |
| Provide justification for your answer to Q42. Selective reporting will lead to bias if it is based on the direction, magnitude or statistical significance of exposure effect estimates | Text |  |
| Q43. What is the predicted direction of bias due to selection of the reported result? | Radio | Towards the null  Away from the null  Unpredictable |
| Provide justification for your answer to Q43. | Text |  |
| Overall risk of bias | | |
| Q44. What is the overall risk of bias for the study (Note that the overall risk of bias for the study should be as bad as the worst bias for any of the individual bias domains, above.) | Radio | Low  Moderate  Serious  Critical |
| Provide justification for your answer to Q44. | Text |  |
| Q45. What is the overall predicted direction of bias for this outcome? | Radio | Towards the null  Away from the null  Unpredictable |
| Provide justification for your answer to Q45. | Text |  |
|  |  |  |

Notes about ROB assessment and ROBINS—I tool

The preliminary version of ROBINS-I forms asked reviewers to specify if the outcome is considered beneficial or harmful. This question was dropped from our risk-of-bias assessment because in this review, the investigators were looking only for potentially harmful outcomes, not beneficial ones, hence the referent category was always the unexposed or least exposed group.

For assessment of confounding bias, reviewers were asked to list the applicable outcome categories in the risk-of-bias form. These outcome categories had been identified in the previous review and comprised: respiratory, gastrointestinal, eye, mood, dermatologic, neurologic, MRSA, otologic, Q Fever, and "other". The category "other" pertained to outcomes that did not fit into the previous categories, for example, aching joints or fever. Before risk-of-bias assessment began, co-authors with expertise in the area were consulted to identify any known critically important confounding domains for each of the outcome categories.

Information on other (i.e. non-critical) confounders was captured by the Outcome-level data extraction form (SM 2) as described above. Questions regarding confounding in the risk-of-bias tools for observational studies therefore relate to critical confounders only. One of the questions in the section pertaining to "bias due to confounding" in the ROBINS-I tool was revised for clarity; we found the original wording in the ROBINS-I form "Is confounding of the effect of intervention unlikely in this study?" could result in a double-negative for an answer, so we reworded this question to "Is confounding of the effect of exposure likely in this study?" For determining the direction of bias due to confounding we found Mehio-Sibai et al. (2005) {Mehio-sabai, 2005} a useful reference for this purpose and so advised reviewers to consult it when making their decision.

Questions about co-interventions found in the section pertaining to bias due to confounding in ROBINS-I were not included in our risk of bias assessment, as they were not considered relevant to the topic of this review. Questions in the ROBINS-I tool regarding post-intervention variables were also not considered applicable for this review. In the domain "bias due to measurement of exposures", we added a question that was not in the ROBINS-I tool: "How accurately did the investigators measure the specified exposure(s)?" as we felt that this was important information to capture for the current review. Two issues arose with respect to this question. First, how well did the exposure measured by the investigators reflect exposure to animal facilities and second, how accurately was the exposure measured? To illustrate this issue, we provide an example; one group of authors used agricultural census data to measure the number of animal houses near each subject's residence. These data are likely an accurate measure of the number of animal houses; however, given topography, directionality of residences relative to prevailing wind direction, etc., this metric may not be an accurate measure of the actual exposure of residents. The section on "bias due to departure from intended interventions" in the ROBINS-I tools was not included in our risk-of-bias tools as this type of bias was not thought to be particularly relevant to the review topic. This bias domain seems to relate to a particular source of mis-measurement of exposure, which is far less relevant than actual mis-measurement.

For "bias due to missing data", as the ROBINS-I tool did not provide guidance in this area, the reviewers used an arbitrary cut-off whereby if < 10% of the reported sample size was missing, the data were considered to be reasonably complete.

SM 4: Risk-of-bias tool for non-randomized studies of exposures (cohort studies) for a systematic review of the association between proximity to animal feeding operations and the health of individuals in nearby communities (modified from a preliminary unpublished version of the ROBINS-I tool).

| Risk of bias for non-randomized studies (cohort studies) | | |
| --- | --- | --- |
| Question | Style | Response |
| Q1. Describe the exposure / outcome grouping. | Radio | Objective exposures / Objective outcomes  Objective exposures / Subjective outcomes  Subjective exposures / Subjective outcomes  Subjective exposures / Objective outcomes |
| Confounding Bias | | |
| Q2. Outcome confounding domains | Checkbox | Respiratory  Gastrointestinal  Mood  Eye  Dermatologic  Lifestyle  Neurologic  MRSA  Otologic  Other |
| Q3. List the critically important confounding domains for respiratory outcomes that the investigators controlled for in this study. | Checkbox (only appears if "Respiratory" was checked for Q2) | Not reported  Family history of allergies or respiratory disease  Presence of wood-burning stove(s)  Exposure to tobacco smoke  Socioeconomic status (may be indicated by race, education, employment status)  Concurrent disease  Presence of cats in the house |
| Q4. List the critically important gastrointestinal outcome confounding domains that the investigators controlled for in this study. | Checkbox (only appears if Gastrointestinal was checked for Q2) | Pre-existing disease |
| Q5. List the critically important mood outcome domains that the investigators controlled for in this study. | Checkbox (only appears if Mood was checked for Q2) | Socioeconomic status  None of the above |
| Q6. List the critically important eye outcome domains that the investigators controlled for in this study. | Checkbox (only appears of Eye was checked for Q2) | Pet ownership  Atopic parents  Factors associated with allergies |
| Q7. List the critically important lifestyle outcome domains that the authors controlled for in this study. | Checkbox (only appears if Lifestyle was checked for Q2) | The lifestyle category was considered too broad to have any specific critical confounders. |
| Q8. List the critically important neurologic outcome domains that the authors controlled for in this study. | Checkbox (only appears if Neurologic was checked for Q2) | Socio-economic status  Pre-existing disease |
| Q9. List the critically important MRSA outcome domains that the authors controlled for in this study. | Checkbox (only appears if MRSA was checked for Q2) | Livestock ownership (horses, cattle)  None of the above |
| Q10. List the critically important dermatologic outcome domains that the authors controlled for in this study. | Checkbox (only appears if Dermatologic was checked for Q2) | Ownership of pets  Atopic parents  Factors associated with allergies |
| Q11. List the critically important otologic outcome domains that the authors controlled for in this study. | Checkbox (only appears if Otologic was checked for Q2) | Some pesticide usage |
| Q12. List the critically important "Other" outcome domains that the authors controlled for in this study. | Checkbox (only appears if Other was checked for Q2) | This category was considered so broad that critically confounding domains could not be identified for this review. |
| Q13. Is confounding of the effect of exposure *likely* in this study?  If No or Probably No the study can be considered to be at low risk of bias due to confounding and no further signalling questions need be considered. | Radio | Yes  Probably Yes  Probably No  No  No information |
| Provide justification for your answer to Q13. | Text |  |
| Q14. Were participants analysed according to their initial exposure group throughout follow up? | Radio | Yes  Probably Yes  Probably No  No  Not applicable  No information |
| Provide justification for your answer to Q14. | Text |  |
| Q15. If you answered "no" or "probably no" to Q14, were exposure discontinuations or switches unlikely to be related to factors that are prognostic for the outcome? | Radio | Yes  Probably Yes  Probably No  No  Not applicable  No information |
| Provide justification for your answer to Q15. | Text |  |
| Q16. If you answered "yes" or "probably yes" to Q14 or Q15, did the investigators use an appropriate analysis method that adjusted for all the critically important confounding domains? | Radio | Yes  Probably Yes  Probably No  No  No information |
| Provide justification for your answer to Q16. | Text |  |
| Q17. If you answered "yes" or "probably yes" to Q16, were critical confounding domains that were adjusted for measured *validly* and *reliably* by the variables available in this study? | Radio | Yes  Probably Yes  Probably No  No  Not applicable  No information |
| Provide an explanation for your answer to Q17. | Text |  |
| Q18. If you answered "yes" or "probably yes" to Q13, did the authors avoid adjusting for post-intervention variables? | Radio | Yes  Probably Yes  Probably No  No  Not applicable  No information |
| Provide an explanation for your answer to Q18. | Text |  |
| Q19. If you answered "no" or "probably no" to Q14 or Q15, did the authors use an appropriate analysis method that adjusted for all the critically important confounding domains and for time-varying confounding? | Radio | Yes  Probably Yes  Probably No  No  Not applicable  No information |
| Provide an explanation to your answer to Q19. | Text |  |
| Q20. If you answered "yes" or "probably yes" to Q19, were confounding domains that were adjusted for measured *validly* and *reliably* by the variables available in this study? | Radio | Yes  Probably Yes  Probably No  No  Not applicable  No information |
| Provide an explanation for your answer to Q20. | Text |  |
| Q21. What is the risk of bias due to confounding in this study? (Refer to p. 20 in Sterne et al. 2014.) | Radio | Low  Moderate  Serious  Critical |
| Please provide justification for your answer to Q21. | Text |  |
| Q22. What is the predicted direction of bias due to confounding?  (For guidance, please refer to Mehio-Sibai et al. 2005.) | Radio | Towards the null  Away from the null  Unpredictable |
| Provide justification for your answer to Q22. | Text |  |
| Selection Bias | | |
| Q23. Was selection into the study unrelated to the exposure or unrelated to outcome? (Good studies would get a "Yes")  Selection bias is an issue if there is differential enrollment of a group in 1 of the cells in the 2X2 table. Differential enrollment based on exposure only would not be an issue of selection bias, nor would it be an issue if they differentially enrolled based on disease-status only. | Radio | Yes  Probably Yes  Probably No  No |
| Provide an explanation for your answer to Q23. | Text |  |
| Q24. Do start of follow-up and start of exposure coincide for all or most subjects? | Radio | Yes  Probably Yes  Probably No  No |
| Provide an explanation for your answer to Q24. | Text |  |
| Q25. If you answered "no" or "probably no" to Q24, were adjustment techniques used that are likely to correct for the presence of selection biases? | Radio | Yes  Probably Yes  Probably No  No |
| Provide an explanation for your answer to Q25. | Text |  |
| Q26. What is the risk of bias due to selection in this study? (Refer to p. 24 in Sterne et al. 2014.)  NB: Low risk of bias means the study is comparable to a well-performed randomized trial. This is unlikely in an observational study. Moderate risk of bias means selection into the study may have been related to exposure and outcome status but the investigators used appropriate methods to adjust for selection bias. | Radio | Low  Moderate  Serious  Critical |
| Justify your answer to Q26. | Text |  |
| Q27. What is the predicted direction of bias due to selection of participants into the study? | Radio | Towards the null  Away from the null  Unpredictable |
| Please give an explanation of your answer to Q27. | Text |  |
| Bias in exposure measurement | | |
| Q28. Was the exposure status well defined? | Radio | Yes  No |
| Provide an explanation for your answer to Q28. | Text |  |
| Q29. How accurately did the investigators measure the specified exposure(s)? | Radio | Relatively accurate  Inaccurate  No information |
| Provide justification for your answer to Q29. | Text |  |
| Q30. Was information on exposure status recorded at the time of exposure? | Radio | Yes  Probably Yes  Probably No  No  Can't tell |
| Justify your answer to Q30. | Text |  |
| Q31. Was information on exposure status unaffected by knowledge of the outcome or risk of the outcome?  (Good studies get a Yes.) | Radio | Yes  Probably Yes  Probably No  No  Not applicable  No information |
| Provide justification for your answer to Q31. | Text |  |
| Q32. What is the risk of bias due to measurement of the exposure? (Refer to p. 27 in Sterne et al. 2014). | Radio | Low  Moderate  Serious  Critical  No information |
| Justify your answer to Q32. | Text |  |
| Q33. What is the predicted direction of bias due to measurement of exposures? | Radio | Towards the null  Away from the null  Unpredictable |
| Provide justification for your answer to Q33. | Text |  |
| Bias due to missing data | | |
| Q40. Was the outcome status reasonably complete for those in whom it was sought?  < 10% data missing constitutes reasonably complete | Radio | Yes  Probably Yes  Probably No  No  No information/numbers not reported |
| Provide justification for your answer to Q40. | Text |  |
| Q41. Were data on exposure status reasonably complete for those in whom it was sought?  < 10% data missing constitutes reasonably complete | Radio | Yes  Probably Yes  Probably No  No  No information/numbers not reported |
| Justify your answer to Q41. | Text |  |
| Q42. Are data reasonably complete for other variables in the analysis?  < 10% data missing is reasonably complete | Radio | Yes  Probably Yes  Probably No  No  No information/numbers not reported |
| Provide justification for your answer to Q42. | Text |  |
| Q43. If you answered "No" to Q40, Q41 or Q42, were the proportion of participants and reasons for missing data similar across exposures? | Radio | Yes  Probably Yes  Probably No  No  No information  Can't tell |
| Provide an explanation for your answer to Q43. | Text |  |
| Q44. If you answered "No" to Q40, Q41 or Q42, were appropriate statistical methods used to account for missing data (i.e. Do the investigators describe missing data imputation)? | Radio | Yes  Probably Yes  Probably No  No  Not applicable  No information |
| Provide an explanation for your answer to Q44. | Text |  |
| Q45. What is the risk of bias due to missing data? (refer to p. 36 in Sterne et al. 2014) | Radio | Low  Moderate  Serious  Critical  Unable to assess |
| Provide justification for your answer to Q45. | Text |  |
| Q46. What is the predicted direction of bias due to missing data?  Depends on what's missing, and most of the time you won't be able to tell the direction of bias. | Radio | Towards the null  Away from the null  Unpredictable |
| Provide justification for your answer to Q46. | Text |  |
| Bias in measurement of outcomes | | |
| Q47. Was the outcome measure objective? | Radio | Yes  Probably Yes  Probably No  No |
| Provide an explanation for your answer to Q47. | Text |  |
| Q48. Were outcome assessors unaware of the exposure received by study participants?  (Will likely be "no" for subjective outcome and "yes" for objective.) | Radio | Yes  No  Can't tell |
| Provide an explanation for your answer to Q48. | Text |  |
| Q49. Were the methods of outcome assessment comparable across exposure groups? | Radio | Yes  Probably Yes  Probably No  No |
| Provide an explanation for your answer to Q49. | Text |  |
| Q50. Were any systematic errors in measurement of the outcome unrelated to the exposure status? | Radio | Yes  No |
| Provide an explanation for your answer to Q50. | Text |  |
| Q51. What is the risk of bias due to measurement of outcomes? (Refer to p.39 in Sterne et al. 2014.) | Radio | Low  Moderate  Serious  Critical  No information |
| Provide an explanation for your answer to Q51. | Text |  |
| Q52. What is the predicted direction of bias due to measurement of outcomes? | Radio | Towards the null  Away from the null  Unpredictable |
| Provide justification for your answer to Q52. | Text |  |
| Bias in selection of reported results | | |
| Q53. Is the reported effect estimate unlikely to be selected, on the basis of the results, due to multiple definitions of the exposure?  Answer "Yes" or "Probably Yes" if, for example, the variable was continuous but was treated as categorical. | Radio | Yes  Probably Yes  Probably No  No |
| Provide an explanation for your answer to Q53. | Text |  |
| Q54. Is the reported effect estimate unlikely to be selected, on the basis of the results, due to multiple analyses of the exposure-outcome relationship? | Radio | Yes  Probably Yes  Probably No  No  Can't tell |
| Provide an explanation for your answer to Q54. | Text |  |
| Q55. Is the reported effect estimate unlikely to be selected, on the basis of the results, due to different subgroups? | Radio | Yes  Probably Yes  Probably No  No |
| Provide an explanation for your answer to Q55. | Text |  |
| Q56. What is the risk of bias due to selection of reported results? (Refer to p.43 in Sterne et al. 2014.) | Radio | Low  Moderate  Serious  Critical  Unable to assess |
| Provide justification for your answer to Q56. Selective reporting will lead to bias if it is based on the direction, magnitude or statistical significance of exposure effect estimates | Text |  |
| Q57. What is the predicted direction of bias due to selection of the reported result? | Radio | Towards the null  Away from the null  Unpredictable |
| Provide justification for your answer to Q57. | Text |  |
| Overall risk of bias | | |
| Q58. What is the overall risk of bias for the study (Note that the overall risk of bias for the study should be as bad as the worst bias for any of the individual bias domains, above.) | Radio | Low  Moderate  Serious  Critical |
| Provide justification for your answer to Q58. | Text |  |
| Q59. What is the overall predicted direction of bias for this outcome? | Radio | Towards the null  Away from the null  Unpredictable |
| Provide justification for your answer to Q59. | Text |  |

SM 5: Risk-of-bias tool used for non-randomized experimental studies in a systematic review of the association between proximity to animal feeding operations and the health of individuals in nearby communities (modified from the Cochrane Risk-of-Bias Tool, Table 8.5a in Higgins and Green 2011).

| Question | Style | Response |
| --- | --- | --- |
| Q1. Please select the exposure/outcome combination | Radio | Objective exposure/Objective outcome  Objective exposure/Subjective outcome  Subjective exposure/Objective outcome  Subjective exposure/Subjective outcome |
| Selection bias | | |
| Q2. What was the risk of bias due to allocation method? (If the authors did not describe the method used to randomize allocation, choose "Unclear". If the authors described the method used to achieve randomization, choose "Low", if the authors did not randomize allocation, choose, "High".) | Radio | Low  High  Unclear |
| Q3. What was the rationale for your assessment of the risk of bias due to allocation method? | Text |  |
| Performance Bias | | |
| Q4. Were measures to blind the subjects or investigators described? | Radio | Yes  No |
| Q5. What was the risk of bias due to knowledge of the allocated exposures by the subjects or investigators during the study? | Radio | Low  High  Unclear |
| Q6. What was the rationale for your assessment of the risk of bias due to blinding of subjects/investigators? | Text |  |
| Detection Bias | | |
| Q7. Did the authors describe measures to blind outcome assessors? | Radio | Yes  No |
| Q8. What is the risk of bias due to knowledge of the allocated exposure by the outcome assessors? | Radio | Low  High  Unclear |
| Q9. What was the rationale for your assessment of the risk of bias due to blinding of outcome assessors? | Text |  |
| Attrition Bias | | |
| Q10. Were there incomplete outcome data in the study? (If this was not reported, choose "unable to assess") | Radio | No loss to follow-up  Loss to follow-up present but explained  Loss to follow-up present but not explained  Unable to assess (numbers not reported comprehensively) |
| Q11. What was the risk of bias due to amount, nature, or handling of incomplete outcome data? (If the authors performed a sensitivity analysis to see how the missing or lost data would have affected the effect measure, then select "Low". If data/animals are missing and the authors do nothing to address this, the risk of bias is High). | Radio | Low  High  Unclear |
| Q12. What is the rationale for your assessment of the risk of bias due to incomplete outcome data? | Text |  |
| Reporting Bias | | |
| Q13. Was there selective reporting of outcomes? | Radio | Yes  No  Unable to discern |
| Q14. What was the risk of bias due to selective outcome reporting? (Might the funding source for the study affect the authors' motivation to report all results?) Answer "High" if it looks like the authors were "data-mining" in order to find any kind of significant difference between intervention and control groups or if the authors reported results in something other than the standard odds ratios or betas. | Radio | Low  High  Unclear |
| Q15. What was the rationale for your assessment of the risk of bias due to selective reporting of outcomes? | Text |  |
| Other Bias | | |
| Q16. Comment on any other potential sources of bias identified e.g. Did the analyses fail to take into account pseudo-replication? We acknowledge that this bias in truth affects precision, rather than a systematic direction bias. | Text |  |
| Q17. Are there concerns about multiplicity? (e.g. If the authors did an ANOVA then did an F-test and it's significant and then the authors looked at all the comparisons within the ANOVA and did a Bonferroni correction within the test, but not correct for multiple comparisons across the study (just within the ANOVA), there are still multiplicity problems if you do, say, 20 ANOVAs, there's still a problem with multiplicity.) | Radio | Yes  No  Unclear |
| Q18. What was the risk of bias due to other potential sources of bias not identified in the preceding questions? | Radio | Low  High  Unclear |
| Q19. What was the rationale for your assessment of the risk of bias due to other sources of bias? | Text |  |
| Q20. Additional Comments (any additional information you feel is relevant to the assessment of risk of bias that was not captured by the previous questions) | Text |  |

SM 6: Full search strategy and results for a database search of Ovid MEDLINE® In-Process & Other Non-Indexed Citations and Ovid MEDLINE® (dates covered: 1946 to Present; date of search 15 September 2014) for a systematic review of the association between proximity to animal feeding operations and the health of individuals in nearby communities.

| Search line | Search string | Number  of hits |
| --- | --- | --- |
| 1 | Animal Husbandry/ | 15,374 |
| 2 | Housing, Animal/ or Animal Feed/ | 44,514 |
| 3 | ((animal$1 or bovine or cow or cows or cattle or beef or pig or pigs or piglet$ or pork or swine or porcine or hog or hogs or finisher$ or sheep or murine or lamb or lambs or poultry or chicken$ or hen or hens or broiler$ or turkey$ or livestock or live stock or intensiv$ or industrial$ or confined or confinement or concentrated or large-scale or high density) adj4 (feed$ operation$ or feed$ facilit$)).ti,ab. | 287 |
| 4 | (cafo or cafos or afo or afos).ti,ab. | 543 |
| 5 | (feed lot$1 or feedlot$ or feedyard$ or feed yard$).ti,ab. | 2329 |
| 6 | ((animal$1 or bovine or cow or cows or cattle or beef or pig or pigs or piglet$ or pork or swine or porcine or hog or hogs or finisher$ or sheep or murine or lamb or lambs or poultry or chicken$ or hen or hens or broiler$ or turkey$ or livestock or live stock) adj (density or operation$ or facility or facilities or confined or confinement)).ti,ab. | 1897 |
| 7 | ((confined or confinement) adj3 (feed or feeding)).ti,ab. | 111 |
| 8 | ((intensive or intensively or intensity or large-scale or industrial or high-density) adj3 (farm or farms or farming or livestock or live stock)).ti,ab. | 994 |
| 9 | ((animal production or livestock production or live stock production) adj (operation$ or facility or facilities)).ti,ab. | 62 |
| 10 | or/1-9 | 60,675 |
| 11 | Environmental Health/ | 12,429 |
| 12 | environmental exposure/ or inhalation exposure/ | 64,342 |
| 13 | environmental pollutants/ or exp air pollutants/ or water pollutants/ | 100,951 |
| 14 | Environmental Illness/ | 939 |
| 15 | Environmental Monitoring/ | 69,965 |
| 16 | (public health$ or environmental health$ or environmental medicine or community health$).ti,ab,jn,jw. | 306,106 |
| 17 | ((public or community or communities or resident$ or residence$1 or living or neighbor$ or neighbour$ or family or families or local$1 or population$1 or populace or school$1 or preschool$ or highschool$ or nursery or nurseries or playgroup$ or play group$ or kindergarten$ or inhabitant$ or household$ or house hold$ or town$ or village$ or city or cities or settlement$) adj5 (proximity or vicinity or location$1 or located or nearby or near or close or closely)).ti,ab. | 37,110 |
| 18 | ((community or communities or resident$ or residence$1 or neighbor$ or neighbour$ or family or families or local$1 or populace$1 or school$1 or preschool$ or highschool$ or nursery or nurseries or playgroup$ or play group$ or kindergarten$ or inhabitant$ or household$ or house hold$ or town$ or village$ or city or cities or settlement$) adj5 (health or disease$1 or sickness$ or illness$ or infect$ or impact$ or effect$1 or exposure$1 or expose$1 or outcome$1 or symptom$1 or risk$1)).ti,ab. | 258,268 |
| 19 | or/11-18 | 726,465 |
| 20 | 10 and 19 | 3346 |
| 21 | exp animals/ not humans/ | 4,009,228 |
| 22 | (news or editorial or letter).pt. | 1,390,080 |
| 23 | foot ortho$.ti,ab. | 1239 |
| 24 | 20 not (21 or 22 or 23) | 1459 |
| 25 | remove duplicates from 24 | 1437 |

Notes about the search

Capturing concepts such as community health outcomes in a robust way is challenging due to the range of free-text and index terms that can be used to describe them. Developing a strategy to attempt to capture these concepts and adapting that strategy for other database interfaces involves inevitable trade-offs to ensure that the volume of search results returned is manageable within the context of the project. Whilst the search strategy was designed to be as sensitive as possible within the time and resource constraints, it is unlikely that any strategy would be able to fully capture these concepts, and there is always the risk of potentially missing relevant studies. However, the additional search techniques (such as checking reference lists) somewhat mitigated the risk of missing relevant studies by providing an alternative method to retrieve any eligible records that were missed by the bibliographic database searches.

Experience from the prior review conducted in this topic area suggested that specific health outcomes, such as respiratory or gastrointestinal diseases, were best characterized during the screening process rather than built into the search strategy. Including the specific health outcomes as part of the community health concept returned a large number of irrelevant records about these conditions in livestock, resulting in a volume of search results much larger than could be screened within the resource constraints of the project. It is recognized that this was a pragmatic decision that could have resulted in relevant studies that described a specific outcome in the title, abstract, or indexing of database records being missed.

SM 7: Full search strategy and results for a database search of Science Citation Index (Web of Knowledge; dates covered 1900 to 13 September 2014; search date 15 September 2014) for a systematic review of the association between proximity to animal feeding operations and the health of individuals in nearby communities.

| Search line | Search string | Number  of hits |
| --- | --- | --- |
| 1 | TS=((animal* or "bovine" or "cow" or "cows" or "cattle" or "beef" or "pig" or "pigs" or piglet* or "pork" or "swine" or "porcine" or "hog" or "hogs" or finisher* or "sheep" or "murine" or "lamb" or "lambs" or "poultry" or chicken* or "hen" or "hens" or broiler* or turkey* or "livestock" or "live stock" or intensiv* or industrial* or "confined" or "confinement" or "concentrated" or "large-scale" or "high density") NEAR/4 ("feed* operation*" or "feed* facilit*")) | 612 |
| 2 | TS=("cafo" or "cafos" or "afo" or "afos") | 692 |
| 3 | TS=("feed lot" or "feed lots" or feedlot* or feedyard* or "feed yard*") | 6886 |
| 4 | TS=((animal* or "bovine" or "cow" or "cows" or "cattle" or "beef" or "pig" or "pigs" or piglet* or "pork" or "swine" or "porcine" or "hog" or "hogs" or finisher* or "sheep" or "murine" or "lamb" or "lambs" or "poultry" or chicken* or "hen" or "hens" or broiler* or turkey* or "livestock" or "live stock") NEAR/1 (operation* or "facility" or "facilities" or "confined" or "confinement")) | 5083 |
| 5 | TS=(("confined" or "confinement") NEAR/3 ("feed" or "feeding")) | 287 |
| 6 | TS=(("intensive" or "intensively" or "intensity" or "large-scale" or "industrial" or "high-density") NEAR/3 ("farm" or "farms" or "farming" or "livestock" or "live stock")) | 3212 |
| 7 | TS=(("animal production" or "livestock production" or "live stock production") NEAR/1 (operation* or "facility" or "facilities")) | 89 |
| 8 | #7 OR #6 OR #5 OR #4 OR #3 OR #2 OR #1 | 15,436 |
| 9 | TS=("public health*" or "environmental health*" or "environmental medicine" or "community health*") | 113,749 |
| 10 | SO=(public health* or environmental health* or environmental medicine or community health*) | 64,163 |
| 11 | TS=(environment* NEAR/1 (exposure* OR expose* OR pollut* OR illness* OR sickness* OR disease*)) | 37,080 |
| 12 | TS=(("public" or "community" or "communities" or "living" or local* or population* or "populace" or resident* or residence* or neighbor* or neighbour* or inhabitant* or household* OR "house hold*" OR town* OR village* OR "city" OR "cities" OR settlement* OR "family" or "families" or school* or preschool* or highschool* or "nursery" or "nurseries" or playgroup* or "play group*" or kindergarten*) NEAR/5 ("proximity" or "vicinity" or location* or "located" or "nearby" or "near" or "close" or "closely")) | 81,235 |
| 13 | TS=(("public" or "community" or "communities" or "living" or local* or population* or "populace" or resident* or residence* or neighbor* or neighbour* or inhabitant* or household* OR "house hold*" OR town* OR village* OR "city" OR "cities" OR settlement* OR "family" or "families" or school* or preschool* or highschool* or "nursery" or "nurseries" or playgroup* or "play group*" or kindergarten*) NEAR/5 ("health" or disease* or sickness* or illness* or infect* or impact* or "effect" or "effects" or exposure* or expose* or outcome* or symptom* or "risk" or "risks")) | 629,523 |
| 14 | TS=("foot ortho*") | 1305 |
| 15 | #13 OR #12 OR #11 OR #10 OR #9 | 787,316 |
| 16 | #15 AND #8 | 870 |
| 17 | #16 not #14 | 869 |

SM 8: Full search strategy and results for a database search of CAB Abstracts (Web of Knowledge) (dates covered 1910 to present; date searched 3 October 2014) for a systematic review of the association between proximity to animal feeding operations and the health of individuals in nearby communities.

| Search line | Search string | Number  of hits |
| --- | --- | --- |
| 1 | TS=((animal* or "bovine" or "cow" or "cows" or "cattle" or "beef" or "pig" or "pigs" or piglet* or "pork" or "swine" or "porcine" or "hog" or "hogs" or finisher* or "sheep" or "murine" or "lamb" or "lambs" or "poultry" or chicken* or "hen" or "hens" or broiler* or turkey* or "livestock" or "live stock" or intensiv* or industrial* or "confined" or "confinement" or "concentrated" or "large-scale" or "high density") NEAR/4 ("feed* operation*" or "feed* facilit*")) | 607 |
| 2 | TS=("cafo" or "cafos" or "afo" or "afos") | 276 |
| 3 | TS=("feed lot" or "feed lots" or feedlot* or feedyard* or "feed yard*") | 7205 |
| 4 | TS=((animal* or "bovine" or "cow" or "cows" or "cattle" or "beef" or "pig" or "pigs" or piglet* or "pork" or "swine" or "porcine" or "hog" or "hogs" or finisher* or "sheep" or "murine" or "lamb" or "lambs" or "poultry" or chicken* or "hen" or "hens" or broiler* or turkey* or "livestock" or "live stock") NEAR/1 (operation* or "facility" or "facilities" or "confined" or "confinement")) | 7322 |
| 5 | TS=(("confined" or "confinement") NEAR/3 ("feed" or "feeding")) | 505 |
| 6 | TS=(("intensive" or "intensively" or "intensity" or "large-scale" or "industrial" or "high-density") NEAR/3 ("farm" or "farms" or "farming" or "livestock" or "live stock")) | 14,176 |
| 7 | TS=(("animal production" or "livestock production" or "live stock production") NEAR/1 (operation* or "facility" or "facilities")) | 178 |
| 8 | #7 OR #6 OR #5 OR #4 OR #3 OR #2 OR #1 | 28,541 |
| 9 | TS=("public health*" or "environmental health*" or "environmental medicine" or "community health*") | 135,343 |
| 10 | SO=(public health* or environmental health* or environmental medicine or community health*) | 8459 |
| 11 | TS=(environment* NEAR/1 (exposure* OR expose* OR pollut* OR illness* OR sickness* OR disease*)) | 77,387 |
| 12 | TS=(("public" or "community" or "communities" or "living" or local* or population* or "populace" or resident* or residence* or neighbor* or neighbour* or inhabitant* or household* OR "house hold*" OR town* OR village* OR "city" OR "cities" OR settlement* OR "family" or "families" or school* or preschool* or highschool* or "nursery" or "nurseries" or playgroup* or "play group*" or kindergarten*) NEAR/5 ("proximity" or "vicinity" or location* or "located" or "nearby" or "near" or "close" or "closely")) | 42,635 |
| 13 | TS=(("public" or "community" or "communities" or "living" or local* or population* or "populace" or resident* or residence* or neighbor* or neighbour* or inhabitant* or household* OR "house hold*" OR town* OR village* OR "city" OR "cities" OR settlement* OR "family" or "families" or school* or preschool* or highschool* or "nursery" or "nurseries" or playgroup* or "play group*" or kindergarten*) NEAR/5 ("health" or disease* or sickness* or illness* or infect* or impact* or "effect" or "effects" or exposure* or expose* or outcome* or symptom* or "risk" or "risks")) | 331,013 |
| 14 | TS=("foot ortho*") | 7 |
| 15 | #13 OR #12 OR #11 OR #10 OR #9 | 442,469 |
| 16 | #15 AND #8 | 2071 |
| 17 | #16 not #14 | 2071 |

SM 9: Results of multiple database searches for a systematic review of the association between proximity to animal feeding operations and the health of individuals in nearby communities

| Database | Number of records | Number of records remaining after de-duplication |
| --- | --- | --- |
| MEDLINE® and MEDLINE® In-Process (Ovid SP) | 1437 | 1437 |
| Science Citation Index (Web of Knowledge) | 869 | 679 |
| Centre for Agricultural Biosciences (CAB) Abstracts (Web of Knowledge) | 2071 | 1581 |
| Total | 4377 | 3697 |

SM 10: List of records excluded at Level 2 (full-text assessment) with reasons for exclusion in a systematic review of the association between proximity to animal feeding operations and the health of individuals in nearby communities.

| Ref ID # | Article | Exclusion Criteria |
| --- | --- | --- |
| 16 | **Ricardo Castillo Neyra, Jose Augusto Frisancho, Jessica L. Rinsky, Carol Resnick, Karen Colleen Carroll, Ana Maria Rule, Tracy Ross, Yaqi You, Lance B. Price, Ellen Kovner Silbergeld**. Multidrug-resistant and methicillin-resistant Staphylococcus aureus (MRSA) in hog slaughter and processing plant workers and their community in North Carolina (USA). *Environmental Health Perspectives.* 2014. 122:471-7 | The unit of analysis was not at the individual human level or the study looked at occupational exposure only. |
| 23 | **P. Levallois, P. Chevalier, S. Gingras, P. Dery, P. Payment, P. Michel, M. Rodriguez**. Risk of infectious gastroenteritis in young children living in Quebec rural areas with intensive animal farming: results of a case-control study (2004-2007). *Zoonoses & Public Health.* 2014. 61:28-38 | The unit of analysis was not at the individual human level or the study looked at occupational exposure only. |
| 24 | **Margaret Carrel, Marin L. Schweizer, Mary Vaughan Sarrazin, Tara C. Smith, Eli N. Perencevich**. Residential proximity to large numbers of swine in feeding operations is associated with increased risk of methicillin-resistant Staphylococcus aureus colonization at time of hospital admission in rural Iowa veterans. *Infection Control & Hospital Epidemiology.* 2014. 35:190-3 | The unit of analysis was not at the individual human level or the study looked at occupational exposure only. |
| 27 | **G. M. Troili, R. Businaro, F. Massoni, L. Ricci, L. Petrone, P. Ricci, S. Ricci**. [Investigation on a group of autistic children: risk factors and medical social considerations]. *Clinica Terapeutica.* 2013. 164:e273-8 | The unit of analysis was not at the individual human level or the study looked at occupational exposure only. |
| 53 | **Margaret A. Davis, Danna L. Moore, Katherine N. K. Baker, Nigel P. French, Marianne Patnode, Joni Hensley, Kathryn Macdonald, Thomas E. Besser**. Risk factors for campylobacteriosis in two washington state counties with high numbers of dairy farms. *Journal of Clinical Microbiology.* 2013. 51:3921-7 | The unit of analysis was not at the individual human level or the study looked at occupational exposure only. |
| 82 | **Monica Monaco, Palmino Pedroni, Andrea Sanchini, Annalisa Bonomini, Annamaria Indelicato, Annalisa Pantosti**. Livestock-associated methicillin-resistant Staphylococcus aureus responsible for human colonization and infection in an area of Italy with high density of pig farming. *BMC Infectious Diseases.* 2013. 13:258 | The unit of analysis was not at the individual human level or the study looked at occupational exposure only. |
| 87 | **Christiane Cuny, Robin Kock, Wolfgang Witte**. Livestock associated MRSA (LA-MRSA) and its relevance for humans in Germany. *Ijmm International Journal of Medical Microbiology.* 2013. 303:331-7 | The unit of analysis was not at the individual human level or the study looked at occupational exposure only. |
| 99 | **Joan A. Casey, Frank C. Curriero, Sara E. Cosgrove, Keeve E. Nachman, Brian S. Schwartz**. High-density livestock operations, crop field application of manure, and risk of community-associated methicillin-resistant Staphylococcus aureus infection in Pennsylvania. *JAMA Internal Medicine.* 2013. 173:1980-90 | The unit of analysis was not at the individual human level or the study looked at occupational exposure only. |
| 104 | **Henri Kabore, Alexandre Lebel, Patrick Levallois, Pascal Michel, Pierre Payment, Pierre Dery, Germain Lebel**. Multilevel analysis of childhood nonviral gastroenteritis associated with environmental risk factors in Quebec, 1999-2006. *Journal of Environmental Health.* 2013. 76:34-45 | The unit of analysis was not at the individual human level or the study looked at occupational exposure only. |
| 105 | **Barbara Zappe Pasturel, Raul Cruz-Cano, Rachel E. Rosenberg Goldstein, Amanda Palmer, David Blythe, Patricia Ryan, Brenna Hogan, Carrianne Jung, Sam W. Joseph, Min Qi Wang, Mei-Ling Ting Lee, Robin Puett, Amy R. Sapkota**. Impact of rurality, broiler operations, and community socioeconomic factors on the risk of campylobacteriosis in Maryland. *American Journal of Public Health.* 2013. 103:2267-75 | The unit of analysis was not at the individual human level or the study looked at occupational exposure only. |
| 126 | **Sara M. Morrow, Jeanette O'Quin, Armando E. Hoet, J. R. Wilkins, Fred DeGraves, Kathleen A. Smith**. Complaints associated with animal feeding facilities as reported to Ohio local health departments, 2006-2008. *Journal of Environmental Health.* 2013. 75:8-13 | The unit of analysis was not at the individual human level or the study looked at occupational exposure only. |
| 139 | **Brian T. Pavilonis, Wayne T. Sanderson, James A. Merchant**. Relative exposure to swine animal feeding operations and childhood asthma prevalence in an agricultural cohort. *Environmental Research.* 2013. 122:74-80 | The unit of analysis was not at the individual human level or the study looked at occupational exposure only. |
| 189 | **S. E. F. Spencer, J. Marshall, R. Pirie, D. Campbell, M. G. Baker, N. P. French**. The spatial and temporal determinants of campylobacteriosis notifications in New Zealand, 2001-2007. *Epidemiology & Infection.* 2012. 140:1663-77 | The unit of analysis was not at the individual human level or the study looked at occupational exposure only. |
| 209 | **Erjia Ge, Robert Haining, Chi Pang Li, Zuguo Yu, Miu Yee Waye, Ka Hou Chu, Yee Leung**. Using knowledge fusion to analyze avian influenza H5N1 in East and Southeast Asia. *PLoS ONE [Electronic Resource].* 2012. 7:e29617 | The unit of analysis was not at the individual human level or the study looked at occupational exposure only. |
| 226 | **Kaye H. Kilburn**. Human impairment from living near confined animal (hog) feeding operations. *Journal Of Environmental & Public Health.* 2012. 2012:565690 | Study did not include more than one unit of measurement of the exposure. |
| 264 | **Wim van der Hoek, Johannes Hunink, Piet Vellema, Peter Droogers**. Q fever in The Netherlands: the role of local environmental conditions. *International Journal of Environmental Health Research.* 2011. 21:441-51 | The unit of analysis was not at the individual human level or the study looked at occupational exposure only. |
| 313 | **Lisa A. King, Laurence Goirand, Herve Tissot-Dupont, Bruno Giunta, Christine Giraud, Claude Colardelle, Veronique Duquesne, Elodie Rousset, Michel Aubert, Richard Thiery, Laurence Calatayud, Gerald Daurat, Laurent Hocqueloux, Valerie Cicchelero, Franck Golliot, Henriette de Valk**. Outbreak of Q fever, Florac, Southern France, Spring 2007. *Vector Borne & Zoonotic Diseases.* 2011. 11:341-7 | The unit of analysis was not at the individual human level or the study looked at occupational exposure only. |
| 340 | **Tilahun Nigatu, Mulu Abraha**. Epidemiological analysis of tuberculosis in Ethiopia: a ten-year trend analysis. *East African Journal of Public Health.* 2010. 7:182-6 | Article could not be procured. |
| 348 | **H. Kabore, P. Levallois, P. Michel, P. Payment, P. Dery, S. Gingras**. Association between potential zoonotic enteric infections in children and environmental risk factors in Quebec, 1999-2006. *Zoonoses & Public Health.* 2010. 57:e195-205 | The unit of analysis was not at the individual human level or the study looked at occupational exposure only. |
| 352 | **K. G. J. Pollock, H. E. Ternent, D. J. Mellor, R. M. Chalmers, H. V. Smith, C. N. Ramsay, G. T. Innocent**. Spatial and temporal epidemiology of sporadic human cryptosporidiosis in Scotland. *Zoonoses & Public Health.* 2010. 57:487-92 | The unit of analysis was not at the individual human level or the study looked at occupational exposure only. |
| 379 | **Paul R. Bessell, Louise Matthews, Alison Smith-Palmer, Ovidiu Rotariu, Norval J. C. Strachan, Ken J. Forbes, John M. Cowden, Stuart W. J. Reid, Giles T. Innocent**. Geographic determinants of reported human Campylobacter infections in Scotland. *BMC Public Health.* 2010. 10:423 | The unit of analysis was not at the individual human level or the study looked at occupational exposure only. |
| 381 | **Jyotsna S. Jagai, Jeffrey K. Griffiths, Paul H. Kirshen, Patrick Webb, Elena N. Naumova**. Patterns of protozoan infections: spatiotemporal associations with cattle density. *Ecohealth.* 2010. 7:33-46 | The unit of analysis was not at the individual human level or the study looked at occupational exposure only. |
| 411 | **Yossi Febriani, Patrick Levallois, Suzanne Gingras, Pierre Gosselin, Shannon E. Majowicz, Manon D. Fleury**. The association between farming activities, precipitation, and the risk of acute gastrointestinal illness in rural municipalities of Quebec, Canada: a cross-sectional study. *BMC Public Health.* 2010. 10:48 | The unit of analysis was not at the individual human level or the study looked at occupational exposure only. |
| 447 | **Louis Anthony Tony Cox**. Why reduced-form regression models of health effects versus exposures should not replace QRA: livestock production and infant mortality as an example. *Risk Analysis.* 2009. 29:1664-71 | The unit of analysis was not at the individual human level or the study looked at occupational exposure only. |
| 462 | **Paul J. Villeneuve, Amira Ali, Laurel Challacombe, Sophie Hebert**. Intensive hog farming operations and self-reported health among nearby rural residents in Ottawa, Canada. *BMC Public Health.* 2009. 9:330 | Study did not include more than one unit of measurement of the exposure. |
| 463 | **S. J. Snel, M. G. Baker, V. Kamalesh, N. French, J. Learmonth**. A tale of two parasites: the comparative epidemiology of cryptosporidiosis and giardiasis. *Epidemiology & Infection.* 2009. 137:1641-50 | The unit of analysis was not at the individual human level or the study looked at occupational exposure only. |
| 464 | **D. L. Pearl, M. Louie, L. Chui, K. Dore, K. M. Grimsrud, S. W. Martin, P. Michel, L. W. Svenson, S. A. McEwen**. A multi-level approach for investigating socio-economic and agricultural risk factors associated with rates of reported cases of Escherichia coli O157 in humans in Alberta, Canada. *Zoonoses & Public Health.* 2009. 56:455-64 | The unit of analysis was not at the individual human level or the study looked at occupational exposure only. |
| 472 | **Christiane Cuny, Rolf Nathaus, Franziska Layer, Birgit Strommenger, Doris Altmann, Wolfgang Witte**. Nasal colonization of humans with methicillin-resistant Staphylococcus aureus (MRSA) CC398 with and without exposure to pigs. *PLoS ONE [Electronic Resource].* 2009. 4:e6800 | The unit of analysis was not at the individual human level or the study looked at occupational exposure only. |
| 489 | **Christine St-Pierre, Patrick Levallois, Suzanne Gingras, Pierre Payment, Marc Gignac**. Risk of diarrhea with adult residents of municipalities with significant livestock production activities. *Journal of Public Health.* 2009. 31:278-85 | The unit of analysis was not at the individual human level or the study looked at occupational exposure only. |
| 502 | **Saskia J. Snel, Michael G. Baker, Kamalesh Venugopal**. The epidemiology of giardiasis in New Zealand, 1997-2006. *New Zealand Medical Journal.* 2009. 122:62-75 | The unit of analysis was not at the individual human level or the study looked at occupational exposure only. |
| 528 | **Steve Wing, Rachel Avery Horton, Stephen W. Marshall, Kendall Thu, Mansoureh Tajik, Leah Schinasi, Susan S. Schiffman**. Air pollution and odor in communities near industrial swine operations. *Environmental Health Perspectives.* 2008. 116:1362-8 | The unit of analysis was not at the individual human level or the study looked at occupational exposure only. |
| 532 | **Sharon K. Greene, Andrew M. Stuart, Felicita M. Medalla, Jean M. Whichard, Robert M. Hoekstra, Tom M. Chiller**. Distribution of multidrug-resistant human isolates of MDR-ACSSuT Salmonella Typhimurium and MDR-AmpC Salmonella Newport in the United States, 2003-2005. *Foodborne Pathogens & Disease.* 2008. 5:669-80 | The unit of analysis was not at the individual human level or the study looked at occupational exposure only. |
| 562 | **Steve Wing, Rachel Avery Horton, Naeema Muhammad, Gary R. Grant, Mansoureh Tajik, Kendall Thu**. Integrating epidemiology, education, and organizing for environmental justice: community health effects of industrial hog operations. *American Journal of Public Health.* 2008. 98:1390-7 | The unit of analysis was not at the individual human level or the study looked at occupational exposure only. |
| 716 | **Sigurdur T. Sigurdarson, Joel N. Kline**. School proximity to concentrated animal feeding operations and prevalence of asthma in students. *Chest.* 2006. 129:1486-91 | The unit of analysis was not at the individual human level or the study looked at occupational exposure only. |
| 814 | **A. Odoi, S. W. Martin, P. Michel, J. Holt, D. Middleton, J. Wilson**. Determinants of the geographical distribution of endemic giardiasis in Ontario, Canada: a spatial modelling approach. *Epidemiology & Infection.* 2004. 132:967-76 | The unit of analysis was not at the individual human level or the study looked at occupational exposure only. |
| 833 | **Katja Radon, Astrid Peters, Georg Praml, Vera Ehrenstein, Anja Schulze, Oliver Hehl, Dennis Nowak**. Livestock odours and quality of life of neighbouring residents. *Annals of Agricultural & Environmental Medicine.* 2004. 11:59-62 | The unit of analysis was not at the individual human level or the study looked at occupational exposure only. |
| 848 | **Bernadette Hood, Terence Seedsman**. Psychosocial investigation of individual and community responses to the experience of Ovine Johne's Disease in rural Victoria. *Australian Journal of Rural Health.* 2004. 12:54-60 | The unit of analysis was not at the individual human level or the study looked at occupational exposure only. |
| 849 | **M. Waser, R. Schierl, E. von Mutius, S. Maisch, D. Carr, J. Riedler, W. Eder, M. Schreuer, D. Nowak, C. Braun-Fahrlander, Alex Study Team**. Determinants of endotoxin levels in living environments of farmers' children and their peers from rural areas. *Clinical & Experimental Allergy.* 2004. 34:389-97 | The unit of analysis was not at the individual human level or the study looked at occupational exposure only. |
| 870 | **Rachel Church Potter, John B. Kaneene, William N. Hall**. Risk factors for sporadic Campylobacter jejuni infections in rural michigan: a prospective case-control study. *American Journal of Public Health.* 2003. 93:2118-23 | The unit of analysis was not at the individual human level or the study looked at occupational exposure only. |
| 887 | **Punam Pahwa, Helen H. McDuffie, James A. Dosman, Diane Robson, John R. McLaughlin, John J. Spinelli, Shirley Fincham**. Exposure to animals and selected risk factors among Canadian farm residents with Hodgkin's disease, multiple myeloma, or soft tissue sarcoma. *Journal of Occupational & Environmental Medicine.* 2003. 45:857-68 | The unit of analysis was not at the individual human level or the study looked at occupational exposure only. |
| 891 | **J. A. Hoppin, D. M. Umbach, S. J. London, M. C. R. Alavanja, D. P. Sandler**. Animal production and wheeze in the Agricultural Health Study: interactions with atopy, asthma, and smoking. *Occupational & Environmental Medicine.* 2003. 60:e3 | The unit of analysis was not at the individual human level or the study looked at occupational exposure only. |
| 934 | **B. M. Sundblad, B. M. Larsson, L. Palmberg, K. Larsson**. Exhaled nitric oxide and bronchial responsiveness in healthy subjects exposed to organic dust. *European Respiratory Journal.* 2002. 20:426-31 | The unit of analysis was not at the individual human level or the study looked at occupational exposure only. |
| 958 | **E. J. Larrieu, M. T. Costa, M. del Carpio, S. Moguillansky, G. Bianchi, Z. E. Yadon**. A case-control study of the risk factors for cystic echinococcosis among the children of Rio Negro province, Argentina. *Annals of Tropical Medicine & Parasitology.* 2002. 96:43-52 | The unit of analysis was not at the individual human level or the study looked at occupational exposure only. |
| 1003 | **O. Vesterberg, L. Palmberg, K. Larsson**. Albumin, transferrin and alpha2-macroglobulin in bronchoalveolar lavage fluid following exposure to organic dust in healthy subjects. *International Archives of Occupational & Environmental Health.* 2001. 74:249-54 | The unit of analysis was not at the individual human level or the study looked at occupational exposure only. |
| 1052 | **S. Wing, S. Wolf**. Intensive livestock operations, health, and quality of life among eastern North Carolina residents. *Environmental Health Perspectives.* 2000. 108:233-8 | Study did not include more than one unit of measurement of the exposure. |
| 1053 | **S. Wing, D. Cole, G. Grant**. Environmental injustice in North Carolina's hog industry. *Environmental Health Perspectives.* 2000. 108:225-31 | The unit of analysis was not at the individual human level or the study looked at occupational exposure only. |
| 1067 | **H. Tissot-Dupont, S. Torres, M. Nezri, D. Raoult**. Hyperendemic focus of Q fever related to sheep and wind. *American Journal of Epidemiology.* 1999. 150:67-74 | The unit of analysis was not at the individual human level or the study looked at occupational exposure only. |
| 1071 | **R. M. Weigel, J. P. Dubey, D. Dyer, A. M. Siegel**. Risk factors for infection with Toxoplasma gondii for residents and workers on swine farms in Illinois. *American Journal of Tropical Medicine & Hygiene.* 1999. 60:793-8 | The unit of analysis was not at the individual human level or the study looked at occupational exposure only. |
| 1105 | **E. Melbostad, W. Eduard, P. Magnus**. Determinants of asthma in a farming population. *Scandinavian Journal of Work, Environment & Health.* 1998. 24:262-9 | The unit of analysis was not at the individual human level or the study looked at occupational exposure only. |
| 1201 | **K. A. Larsson, A. G. Eklund, L. O. Hansson, B. M. Isaksson, P. O. Malmberg**. Swine dust causes intense airways inflammation in healthy subjects. *American Journal of Respiratory & Critical Care Medicine.* 1994. 150:973-7 | The unit of analysis was not at the individual human level or the study looked at occupational exposure only. |
| 1218 | **I. Hatzissabas, G. R. Krueger, J. R. Medina, V. A. Bedoya, T. Papadakis**. Environmental pollution and malignant lymphomas: a tentative contribution to geographic pathology. *Anticancer Research.* 1993. 13:411-7 | The unit of analysis was not at the individual human level or the study looked at occupational exposure only. |
| 1257 | **S. M. Malkhazova, TYu Karimova**. Assessment of the medico-geographical consequences of farming (the case study of natural endemic diseases in Africa). *Geographia Medica.* 1990. 20:51-60 | The unit of analysis was not at the individual human level or the study looked at occupational exposure only. |
| 1293 | **A. Thelin**. Endotoxins in poultry production and human lung reactions. *European Journal of Respiratory Diseases - Supplement.* 1987. 154:65-70 | The unit of analysis was not at the individual human level or the study looked at occupational exposure only. |
| 1753 | **I. Karagiannis, B. Schimmer, A. van Lier, A. Timen, P. Schneeberger, B. van Rotterdam, A. de Bruin, C. Wijkmans, A. Rietveld, Y. van Duynhoven**. Investigation of a Q fever outbreak in a rural area of The Netherlands. *Epidemiology and Infection.* 2009. 137:1283-1294 | Study did not include more than one unit of measurement of the exposure. |
| 1758 | **Y. Febriani, P. Levallois, G. Lebel, S. Gingras**. Association between indicators of livestock farming intensity and hospitalization rate for acute gastroenteritis. *Epidemiology and Infection.* 2009. 137:1073-1085 | The unit of analysis was not at the individual human level or the study looked at occupational exposure only. |
| 1788 | **S. Sneeringer**. Does Animal Feeding Operation Pollution Hurt Public Health ? A National Longitudinal Study of Health Externalities Identified by Geographic Shifts in Livestock Production. *American Journal of Agricultural Economics.* 2009. 91:124-137 | The unit of analysis was not at the individual human level or the study looked at occupational exposure only. |
| 1888 | **M. Hoopmann, T. Werfel**. Identification of overreporting of allergic symptoms in relation to the neighborhood to livestock production facilities as a potential environmental risk factor. *Allergologie.* 2006. 29:307-314 | The unit of analysis was not at the individual human level or the study looked at occupational exposure only. |
| 2058 | **B. Steinheider**. Environmental odours and somatic complaints. *Zentralblatt Fur Hygiene Und Umweltmedizin.* 1999. 202:101-119 | Study did not include more than one unit of measurement of the exposure. |
| 2086 | **B. Steinheider, R. Both, G. Winneke**. Field studies on environmental odors inducing annoyance as well as gastric and general health-related symptoms. *Journal of Psychophysiology.* 1998. 12:64-79 | Study did not include more than one unit of measurement of the exposure. |
| 2155 | **F. Kovacs**. Public-Health Aspects of Environmental-Pollution by Modern Intensive Livestock Production. *Wiener Tierarztliche Monatsschrift.* 1990. 77:204-208 | English translation not available |
| 2210 | . Proceedings of a meeting on the protection of residents living near intensively managed animals, held at the Institute for Animal Hygiene and Animal Welfare of the Hannover Veterinary School on 16 October 1996 Sonderheft. Tagung "Anwohnerschutz bei Intensivtierhaltung" im Institut fur Tierhygiene und Tierschutz der Tierarztliche Hochschule Hannover, Hannover-Kirchrode, 16. Oktober 1996. *Deutsche Tierarztliche Wochenschrift.* 1998. 105:211-250 | The unit of analysis was not at the individual human level or the study looked at occupational exposure only. |
| 2533 | **L. B. DeLind**. Social consequences of intensive swine production: some effects of community conflict. *Culture & Agriculture.* 2004. 26:80-89 | The unit of analysis was not at the individual human level or the study looked at occupational exposure only. |
| 3562 | **G. P. da Silva, S. M. T. Marques**. Effect of odor emissions from pig livestock on the health of rural residents of Concordia, Santa Catarina, Brazil Impacto dos maus odores decorrentes da suinocultura na saude de moradores rurais no municipio de Concordia, Santa Catarina, Brasil. *Revista de Ciencias Agroveterinarias.* 2004. 3:135-141 | The unit of analysis was not at the individual human level or the study looked at occupational exposure only. |
| 3605 | **B. Steinheider, R. Both, G. Winneke**. Assessment of odour annoyance in the vicinity of livestock buildings: an effect-related comparison of exposure assessment by either dispersion calculation or systematic field observations Die Erfassung der Geruchsbelastigung durch Tierstallimmissionen bei Anwohnern. Ein wirkungsbezogener Vergleich der Expositionserfassung durch Ausbreitungsrechnung und Rasterbegehung. *Gefahrstoffe Reinhaltung der Luft.* 1998. 58:411-416 | Study did not include more than one unit of measurement of the exposure. |
| 3643 | **J. M. Sweeten, J. R. Miner**. Odor intensities at cattle feedlots in nuisance litigation. *Paper - American Society of Agricultural Engineers.* 1992. #volume#:23 pp. | The unit of analysis was not at the individual human level or the study looked at occupational exposure only. |
| 3679 | **K. Thu, K. Donham, R. Ziegenhorn, S. Reynolds, P. Subramanian, P. Whitten, J. Stookesberry, P. S. Thorne**. A control study of the physical and mental health of residents living near a large-scale swine operation. *Journal of Agricultural Safety and Health.* 1997. 3:13-26 | Study did not include more than one unit of measurement of the exposure. |
| 3789 | **M. S. Whittington, K. J. Warner**. Large-scale dairies and their neighbors: a case study of the perceived risk in two counties. *Journal of Extension.* 2006. 44:unpaginated | The unit of analysis was not at the individual human level or the study looked at occupational exposure only. |
| 3800 | **S. Wing**. Social responsibility and research ethics in community-driven studies of industrialized hog production. *Environmental Health Perspectives.* 2002. 110:437-444 | The unit of analysis was not at the individual human level or the study looked at occupational exposure only. |
| 4002 | **Heederik D.J.J.,IJzermans C.J.**. Potential effects of intensive livestock farming on neighboring residents. *#journal#.* 2011. #volume#:#pages# | English translation not available |
