# Supplementary material for: Updated systematic review: associations between proximity to animal feeding operations and health of individuals in nearby communities
Source: Syst Rev. 2017 Apr 18;6:86. doi: 10.1186/s13643-017-0465-z (PMC5395850; doi:10.1186/s13643-017-0465-z)

Study	Outcome variable	Exposure variable	Subcategory	95% CI
**Objective Exposures /**	**Subjective Outcomes**			
Mirabelli et al. 2006	Current wheeze children without Self−Reported Allergies	Exposure category	None	1.00
Mirabelli et al. 2006	Current wheeze children without Self−Reported Allergies	Exposure category	Low	1.09 [0.95, 1.23]
Mirabelli et al. 2006	Current wheeze children without Self−Reported Allergies	Exposure category	Medium	1.01 [0.89, 1.13]
Mirabelli et al. 2006	Current wheeze children without Self−Reported Allergies	Exposure category	High	0.97 [0.84, 1.10]
Mirabelli et al. 2006	Current wheeze children with Self−Reported Allergies	Exposure category	None	1.00
Mirabelli et al. 2006	Current wheeze children with Self−Reported Allergies	Exposure category	Low	1.10 [1.03, 1.18]
Mirabelli et al. 2006	Current wheeze children with Self−Reported Allergies	Exposure category	Medium	1.04 [0.97, 1.12]
Mirabelli et al. 2006	Current wheeze children with Self−Reported Allergies	Exposure category	High	1.01 [0.93, 1.08]
Mirabelli et al. 2006	Current wheeze in all children	Exposure category	None	1.00
Mirabelli et al. 2006	Current wheeze in all children	Exposure category	Low	1.09 [1.01, 1.18]
Mirabelli et al. 2006	Current wheeze in all children	Exposure category	Medium	1.03 [0.96, 1.11]
Mirabelli et al. 2006	Current wheeze in all children	Exposure category	High	1.00 [0.92, 1.08]
Mirabelli et al. 2006	Current wheeze children without Self−Reported Allergies	Hog pounds (in millions) within 3 miles of school	None	1.00
Mirabelli et al. 2006	Current wheeze children without Self−Reported Allergies	Hog pounds (in millions) within 3 miles of school	0.1 to <2.0	1.03 [0.93, 1.12]
Mirabelli et al. 2006	Current wheeze children without Self−Reported Allergies	Hog pounds (in millions) within 3 miles of school	2.0 to <5.0	0.99 [0.81, 1.16]
Mirabelli et al. 2006	Current wheeze children without Self−Reported Allergies	Hog pounds (in millions) within 3 miles of school	>=5.0	1.04 [0.85, 1.23]
Mirabelli et al. 2006	Current wheeze children with Self−Reported Allergies	Hog pounds (in millions) within 3 miles of school	None	1.00
Mirabelli et al. 2006	Current wheeze children with Self−Reported Allergies	Hog pounds (in millions) within 3 miles of school	0.1 to <2.0	1.07 [1.01, 1.12]
Mirabelli et al. 2006	Current wheeze children with Self−Reported Allergies	Hog pounds (in millions) within 3 miles of school	2.0 to <5.0	1.04 [0.93, 1.14]
Mirabelli et al. 2006	Current wheeze children with Self−Reported Allergies	Hog pounds (in millions) within 3 miles of school	>=5.0	1.00 [0.89, 1.11]
Mirabelli et al. 2006	Current wheeze in all children	Hog pounds (in millions) within 3 miles of school	None	1.00
Mirabelli et al. 2006	Current wheeze in all children	Hog pounds (in millions) within 3 miles of school	0.1 to <2.0	1.05 [1.00, 1.11]
Mirabelli et al. 2006	Current wheeze in all children	Hog pounds (in millions) within 3 miles of school	2.0 to <5.0	1.01 [0.91, 1.12]
Mirabelli et al. 2006	Current wheeze in all children	Hog pounds (in millions) within 3 miles of school	>=5.0	1.02 [0.91, 1.13]
Mirabelli et al. 2006	Current wheeze children without Self−Reported Allergies	Miles to nearest swine CAFO	>3	1.00
Mirabelli et al. 2006	Current wheeze children without Self−Reported Allergies	Miles to nearest swine CAFO	<=3	1.02 [0.94, 1.11]
Mirabelli et al. 2006	Current wheeze children without Self−Reported Allergies	Miles to nearest swine CAFO	2 to <=3	1.08 [0.95, 1.21]
Mirabelli et al. 2006	Current wheeze children without Self−Reported Allergies	Miles to nearest swine CAFO	<=2	0.99 [0.89, 1.09]
Mirabelli et al. 2006	Current wheeze children with Self−Reported Allergies	Miles to nearest swine CAFO	>3	1.00
Mirabelli et al. 2006	Current wheeze children with Self−Reported Allergies	Miles to nearest swine CAFO	<=3	1.05 [1.00, 1.10]
Mirabelli et al. 2006	Current wheeze children with Self−Reported Allergies	Miles to nearest swine CAFO	2 to <=3	1.12 [1.04, 1.19]
Mirabelli et al. 2006	Current wheeze children with Self−Reported Allergies	Miles to nearest swine CAFO	<=2	1.01 [0.95, 1.07]
Mirabelli et al. 2006	Current wheeze in all children	Miles to nearest swine CAFO	>3	1.00
Mirabelli et al. 2006	Current wheeze in all children	Miles to nearest swine CAFO	<=3	1.04 [0.99, 1.09]
Mirabelli et al. 2006	Current wheeze in all children	Miles to nearest swine CAFO	2 to <=3	1.10 [1.02, 1.18]
Mirabelli et al. 2006	Current wheeze in all children	Miles to nearest swine CAFO	<=2	1.01 [0.95, 1.07]
Mirabelli et al. 2006	Asthma medication use in past year all children	PR (95% CI) for <=3 vs >3 Miles From Nearest Swine CAFO		1.07 [1.00, 1.15]
Mirabelli et al. 2006	Asthma medication use in past year no self−reported allergies	PR (95% CI) for <=3 vs >3 Miles From Nearest Swine CAFO		1.03 [0.88, 1.18]
Mirabelli et al. 2006	Asthma medication use in past year self−reported allergies	PR (95% CI) for <=3 vs >3 Miles From Nearest Swine CAFO		1.09 [1.00, 1.18]
Mirabelli et al. 2006	Asthma−related physician visit emergency visit and/or hospitalization in past year all children	PR (95% CI) for <=3 vs >3 Miles From Nearest Swine CAFO		1.06 [1.00, 1.12]
Mirabelli et al. 2006	Asthma−related physician visit emergency visit and/or hospitalization in past year no self−reported allergies	PR (95% CI) for <=3 vs >3 Miles From Nearest Swine CAFO		1.03 [0.92, 1.13]
Mirabelli et al. 2006	Asthma−related physician visit emergency visit and/or hospitalization in the past year self−reported allergies	PR (95% CI) for <=3 vs >3 Miles From Nearest Swine CAFO		1.06 [1.00, 1.13]
Mirabelli et al. 2006	Current wheeze without physician diagnosis in all children	PR (95% CI) for <=3 vs >3 Miles From Nearest Swine CAFO		1.04 [0.98, 1.11]
Mirabelli et al. 2006	Current wheeze without physician diagnosis in children with no self−reported allergies	PR (95% CI) for <=3 vs >3 Miles From Nearest Swine CAFO		0.99 [0.90, 1.08]
Mirabelli et al. 2006	Current wheeze without physician diagnosis in children with self−reported allergies	PR (95% CI) for <=3 vs >3 Miles From Nearest Swine CAFO		1.08 [1.01, 1.15]
Mirabelli et al. 2006	Frequent severe wheeze in all children	PR (95% CI) for <=3 vs >3 Miles From Nearest Swine CAFO		1.01 [0.92, 1.09]
Mirabelli et al. 2006	Frequent severe wheeze in chidlren with no self reported allergies	PR (95% CI) for <=3 vs >3 Miles From Nearest Swine CAFO		0.97 [0.80, 1.14]
Mirabelli et al. 2006	Frequent severe wheeze in children wth self reported allergies	PR (95% CI) for <=3 vs >3 Miles From Nearest Swine CAFO		1.02 [0.92, 1.11]
Mirabelli et al. 2006	Missed school in past year as a result of asthma symptoms	PR (95% CI) for <=3 vs >3 Miles From Nearest Swine CAFO		1.06 [0.98, 1.14]
Mirabelli et al. 2006	Physician diagnoses asthma in all children	PR (95% CI) for <=3 vs >3 Miles From Nearest Swine CAFO		1.07 [1.01, 1.14]
Mirabelli et al. 2006	Physician diagnosied Asthma in children with no self reported allergies	PR (95% CI) for <=3 vs >3 Miles From Nearest Swine CAFO		1.14 [1.01, 1.26]
Mirabelli et al. 2006	Physician diagnosied Asthma in children with self reported allergies	PR (95% CI) for <=3 vs >3 Miles From Nearest Swine CAFO		1.06 [0.99, 1.12]
Mirabelli et al. 2006	Severe Wheeze in all children	PR (95% CI) for <=3 vs >3 Miles From Nearest Swine CAFO		1.02 [0.97, 1.07]
Mirabelli et al. 2006	Severe wheeze in children with no self reported allergies	PR (95% CI) for <=3 vs >3 Miles From Nearest Swine CAFO		1.05 [0.96, 1.14]
Mirabelli et al. 2006	Severe wheeze in children with self reported allergies	PR (95% CI) for <=3 vs >3 Miles From Nearest Swine CAFO		1.01 [0.96, 1.07]
**Subjective Exposures /**	**Subjective Outcomes**			
Mirabelli et al. 2006	Current wheeze children without Self−Reported Allergies	Livestock odor	None	1.00
Mirabelli et al. 2006	Current wheeze children without Self−Reported Allergies	Livestock odor	Outside school only	0.94 [0.85, 1.02]
Mirabelli et al. 2006	Current wheeze children without Self−Reported Allergies	Livestock odor	Outside AND inside <2 times/mo	1.04 [0.93, 1.15]
Mirabelli et al. 2006	Current wheeze children without Self−Reported Allergies	Livestock odor	Outside inside >= 2 times/mo	1.21 [0.85, 1.57]
Mirabelli et al. 2006	Current wheeze children with Self−Reported Allergies	Livestock odor	None	1.00
Mirabelli et al. 2006	Current wheeze children with Self−Reported Allergies	Livestock odor	Outside school only	1.04 [0.98, 1.09]
Mirabelli et al. 2006	Current wheeze children with Self−Reported Allergies	Livestock odor	Outside AND inside <2 times/mo	0.99 [0.93, 1.06]
Mirabelli et al. 2006	Current wheeze children with Self−Reported Allergies	Livestock odor	Outside inside >= 2 times/mo	1.24 [1.03, 1.44]
Mirabelli et al. 2006	Current wheeze in all children	Livestock odor	None	1.00
Mirabelli et al. 2006	Current wheeze in all children	Livestock odor	Outside school only	1.00 [0.95, 1.06]
Mirabelli et al. 2006	Current wheeze in all children	Livestock odor	Outside AND inside <2 times/mo	1.01 [0.94, 1.07]
Mirabelli et al. 2006	Current wheeze in all children	Livestock odor	Outside inside >= 2 times/mo	1.23 [1.01, 1.44]
Mirabelli et al. 2006	Asthma medication use in past year all children	Livestock Odor Reported Outside or Inside School Building Versus No Reported Odor		1.03 [0.96, 1.10]
Mirabelli et al. 2006	Asthma medication use in past year no self−reported allergies	Livestock Odor Reported Outside or Inside School Building Versus No Reported Odor		1.02 [0.89, 1.15]
Mirabelli et al. 2006	Asthma medication use in past year self−reported allergies	Livestock Odor Reported Outside or Inside School Building Versus No Reported Odor		1.03 [0.96, 1.11]
Mirabelli et al. 2006	Asthma−related physician visit emergency visit and\n/or hospitalization in past year all children	Livestock Odor Reported Outside or Inside School Building Versus No Reported Odor		1.00 [0.95, 1.05]
Mirabelli et al. 2006	Asthma−related physician visit emergency visit and\n/or hospitalization in past year no self−reported allergies	Livestock Odor Reported Outside or Inside School Building Versus No Reported Odor		1.01 [0.91, 1.10]
Mirabelli et al. 2006	Asthma−related physician visit emergency visit and\n/or hospitalization in the past year self−reported allergies	Livestock Odor Reported Outside or Inside School Building Versus No Reported Odor		0.99 [0.94, 1.05]
Mirabelli et al. 2006	Current wheeze children without Self−Reported Allergies	Livestock Odor Reported Outside or Inside School Building Versus No Reported Odor		0.99 [0.91, 1.06]
Mirabelli et al. 2006	Current wheeze children with Self−Reported Allergies	Livestock Odor Reported Outside or Inside School Building Versus No Reported Odor		1.03 [0.98, 1.07]
Mirabelli et al. 2006	Current wheeze in all children	Livestock Odor Reported Outside or Inside School Building Versus No Reported Odor		1.01 [0.97, 1.06]
Mirabelli et al. 2006	Current wheeze without physician diagnosis in all children	Livestock Odor Reported Outside or Inside School Building Versus No Reported Odor		1.01 [0.96, 1.07]
Mirabelli et al. 2006	Current wheeze without physician diagnosis in children with no self−reported allergies	Livestock Odor Reported Outside or Inside School Building Versus No Reported Odor		0.99 [0.90, 1.07]
Mirabelli et al. 2006	Current wheeze without physician diagnosis in children with self−reported allergies	Livestock Odor Reported Outside or Inside School Building Versus No Reported Odor		1.04 [0.97, 1.10]
Mirabelli et al. 2006	Frequent severe wheeze in all children	Livestock Odor Reported Outside or Inside School Building Versus No Reported Odor		1.06 [0.98, 1.14]
Mirabelli et al. 2006	Frequent severe wheeze in chidlren with no self reported allergies	Livestock Odor Reported Outside or Inside School Building Versus No Reported Odor		1.10 [0.92, 1.28]
Mirabelli et al. 2006	Frequent severe wheeze in children wth self reported allergies	Livestock Odor Reported Outside or Inside School Building Versus No Reported Odor		1.04 [0.95, 1.14]
Mirabelli et al. 2006	Missed school in past year as a result of asthma symptoms	Livestock Odor Reported Outside or Inside School Building Versus No Reported Odor		1.02 [0.94, 1.09]
Mirabelli et al. 2006	Physician diagnoses asthma in all children	Livestock Odor Reported Outside or Inside School Building Versus No Reported Odor		1.01 [0.95, 1.06]
Mirabelli et al. 2006	Physician diagnosied Asthma in children with no self reported allergies	Livestock Odor Reported Outside or Inside School Building Versus No Reported Odor		1.04 [0.93, 1.15]
Mirabelli et al. 2006	Physician diagnosied Asthma in children with self reported allergies	Livestock Odor Reported Outside or Inside School Building Versus No Reported Odor		1.00 [0.94, 1.06]
Mirabelli et al. 2006	Severe Wheeze in all children	Livestock Odor Reported Outside or Inside School Building Versus No Reported Odor		1.05 [1.00, 1.10]
Mirabelli et al. 2006	Severe wheeze in children with no self reported allergies	Livestock Odor Reported Outside or Inside School Building Versus No Reported Odor		1.00 [0.91, 1.08]
Mirabelli et al. 2006	Severe wheeze in children with self reported allergies	Livestock Odor Reported Outside or Inside School Building Versus No Reported Odor		1.06 [1.01, 1.12]

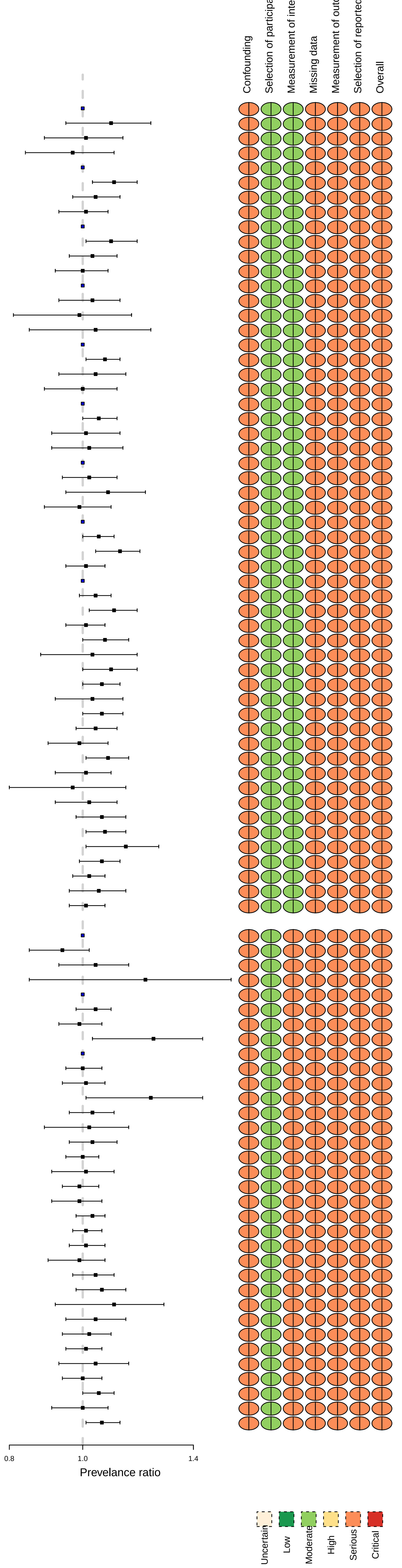

Supplement: Additional file 1: — Discussion about other outcomes included in the systematic review. Excel Spreadsheet with extracted data from review. Data extraction forms, risk of bias forms and search startergies used for review. Figure S1. Neurological and psychological symptoms and stress outcomes for which the effect size was reported as an odds ratio. Figure S2. Neurological symptoms for which the effect size was reported as a regression coefficient. Figure S3. Psychological outcomes for which the effect size was reported as a point estimate of the mean difference. Figure S4. Psychological outcomes for which the effect size was reported as a point estimate. Figure S5. Psychological outcomes for which the effect size was reported as a regression coefficient. Figure S6. Dermatologic, otologic, and optical outcomes for which the effect size was reported as a regression coefficient. Figure S7. Gastrointestinal and “Other” outcomes for which the effect size was reported as a regression coefficient (β). Figure S8. Stress outcomes for which the effect size was reported as a regression coefficient (β). Figure S9. Lower respiratory outcomes for which the effect size was reported as a prevalence ratio. (ZIP 1.40 mb) [file 13643_2017_465_MOESM1_ESM.zip › figS9R1.pdf]
