# Supplementary material for: Updated systematic review: associations between proximity to animal feeding operations and health of individuals in nearby communities
Source: Syst Rev. 2017 Apr 18;6:86. doi: 10.1186/s13643-017-0465-z (PMC5395850; doi:10.1186/s13643-017-0465-z)
Supplement: Additional file 1: — Discussion about other outcomes included in the systematic review. Excel Spreadsheet with extracted data from review. Data extraction forms, risk of bias forms and search startergies used for review. Figure S1. Neurological and psychological symptoms and stress outcomes for which the effect size was reported as an odds ratio. Figure S2. Neurological symptoms for which the effect size was reported as a regression coefficient. Figure S3. Psychological outcomes for which the effect size was reported as a point estimate of the mean difference. Figure S4. Psychological outcomes for which the effect size was reported as a point estimate. Figure S5. Psychological outcomes for which the effect size was reported as a regression coefficient. Figure S6. Dermatologic, otologic, and optical outcomes for which the effect size was reported as a regression coefficient. Figure S7. Gastrointestinal and “Other” outcomes for which the effect size was reported as a regression coefficient (β). Figure S8. Stress outcomes for which the effect size was reported as a regression coefficient (β). Figure S9. Lower respiratory outcomes for which the effect size was reported as a prevalence ratio. (ZIP 1.40 mb) [file 13643_2017_465_MOESM1_ESM.zip › figS5R1.pdf]

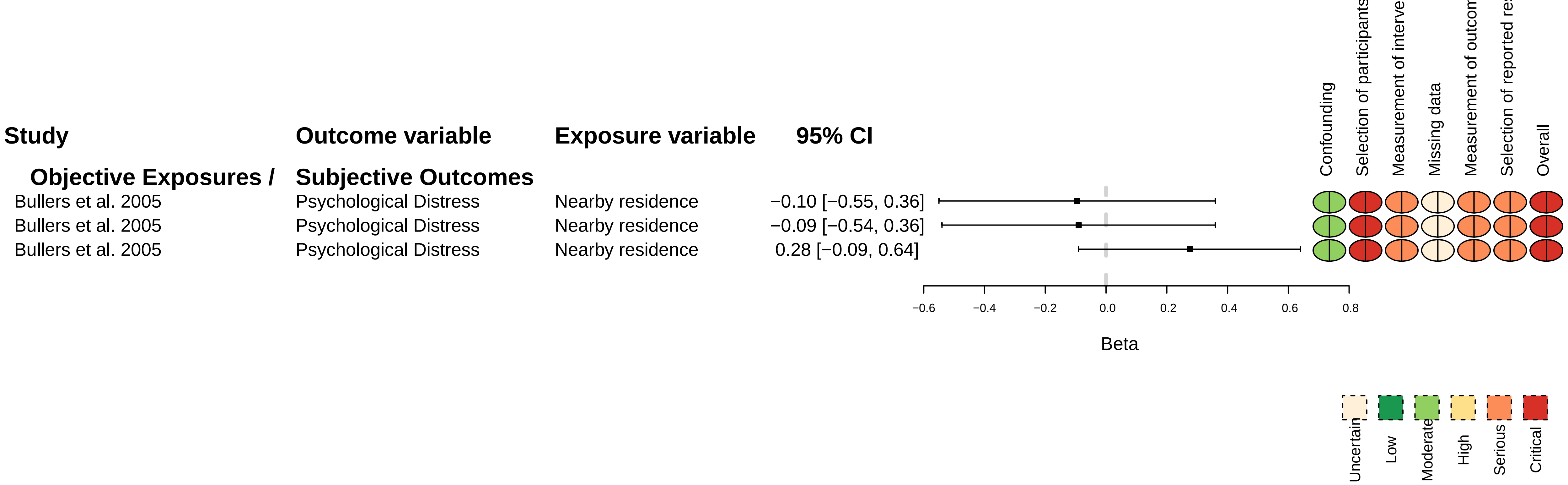
Study
Outcome variable
Exposure variable
95% CI
Confounding
Selection of participants
Measurement of interve
Missing data
Measurement of outcom
Selection of reported re
Overall
Objective Exposures /
Subjective Outcomes
Bullers et al. 2005
Psychological Distress
Nearby residence
−0.10 [−0.55, 0.36]
Bullers et al. 2005
Psychological Distress
Nearby residence
−0.09 [−0.54, 0.36]
Bullers et al. 2005
Psychological Distress
Nearby residence
0.28 [−0.09, 0.64]
−0.6
−0.4
−0.2
0.0
0.2
0.4
0.6
0.8
Beta
Uncertain
Low
Moderate
High
Serious
Critical
