# Supplementary material for: Updated systematic review: associations between proximity to animal feeding operations and health of individuals in nearby communities
Source: Syst Rev. 2017 Apr 18;6:86. doi: 10.1186/s13643-017-0465-z (PMC5395850; doi:10.1186/s13643-017-0465-z)

Study	Outcome variable	Exposure variable	Effect size
**Objective Exposures /**	**Subjective Outcomes**		
Schiffman et al. 1995	Anger based on POM score	Could smell hog odor	21.00
Schiffman et al. 1995	Confusion based on POM score	Could smell hog odor	8.00
Schiffman et al. 1995	Depression based on POM score	Could smell hog odor	19.00
Schiffman et al. 1995	Fatigue based on POM score	Could smell hog odor	10.00
Schiffman et al. 1995	TMD (total mood disturance score)	Could smell hog odor	88.00
Schiffman et al. 1995	Vigor based on POM score	Could smell hog odor	−3.00

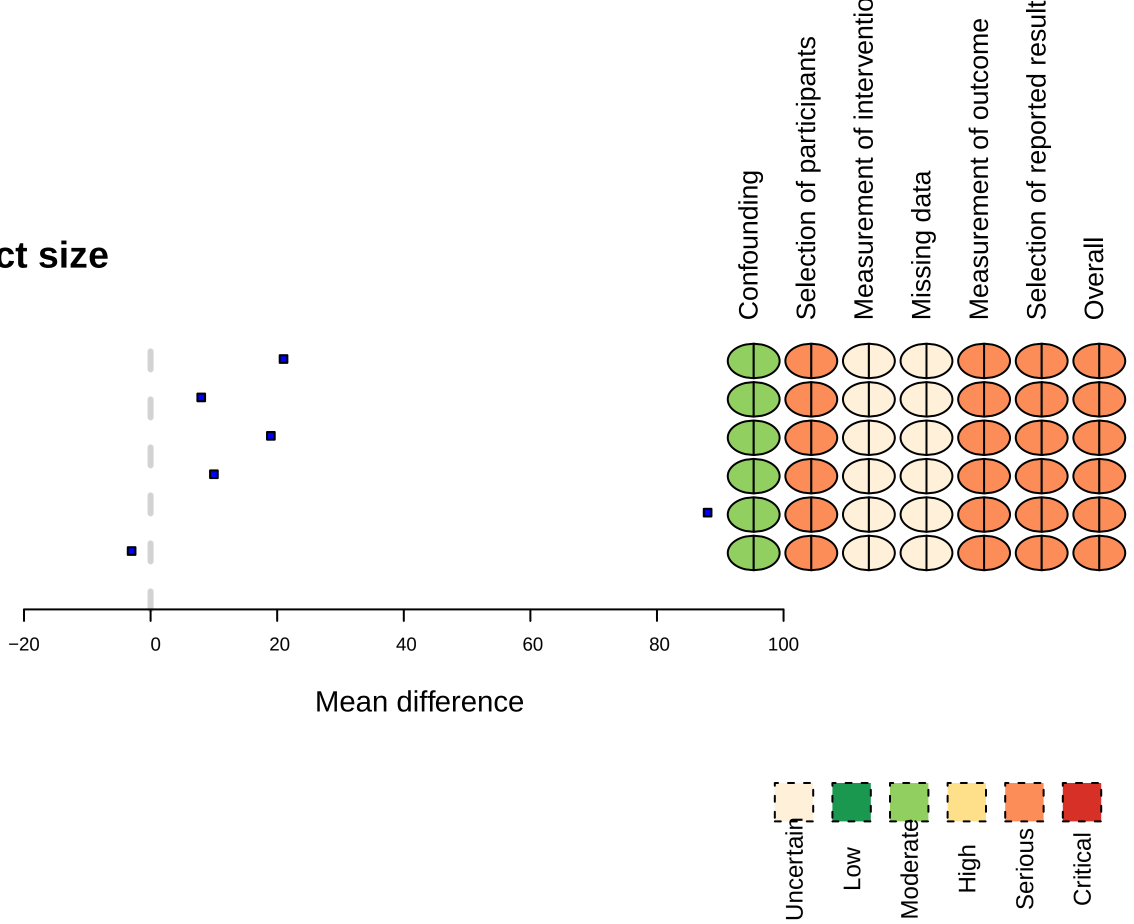

Supplement: Additional file 1: — Discussion about other outcomes included in the systematic review. Excel Spreadsheet with extracted data from review. Data extraction forms, risk of bias forms and search startergies used for review. Figure S1. Neurological and psychological symptoms and stress outcomes for which the effect size was reported as an odds ratio. Figure S2. Neurological symptoms for which the effect size was reported as a regression coefficient. Figure S3. Psychological outcomes for which the effect size was reported as a point estimate of the mean difference. Figure S4. Psychological outcomes for which the effect size was reported as a point estimate. Figure S5. Psychological outcomes for which the effect size was reported as a regression coefficient. Figure S6. Dermatologic, otologic, and optical outcomes for which the effect size was reported as a regression coefficient. Figure S7. Gastrointestinal and “Other” outcomes for which the effect size was reported as a regression coefficient (β). Figure S8. Stress outcomes for which the effect size was reported as a regression coefficient (β). Figure S9. Lower respiratory outcomes for which the effect size was reported as a prevalence ratio. (ZIP 1.40 mb) [file 13643_2017_465_MOESM1_ESM.zip › figS3R1.pdf]
