# Supplementary material for: Updated systematic review: associations between proximity to animal feeding operations and health of individuals in nearby communities
Source: Syst Rev. 2017 Apr 18;6:86. doi: 10.1186/s13643-017-0465-z (PMC5395850; doi:10.1186/s13643-017-0465-z)

Study	Outcome variable	Exposure variable	95% CI
**Neurologic**			
**Objective Exposures /**	**Subjective Outcomes**		
Horton et al. 2009	Confused or unable to concentrate	Hydrogen sulfide (ppb)	0.93 [0.73, 1.17]
Horton et al. 2009	Confused or unable to concentrate	PM10 (ug/m3)	1.01 [1.00, 1.02]
Horton et al. 2009	Confused or unable to concentrate	Semivolatile PM10 (ug/m3)	1.04 [0.97, 1.12]
**Subjective Exposures /**	**Subjective Outcomes**		
Horton et al. 2009	Confused or unable to concentrate	Twice daily odor rating (0−8)	1.31 [1.16, 1.50]
**Psychological**			
**Objective Exposures /**	**Subjective Outcomes**		
Horton et al. 2009	Angry grouchy or bad−tempered	Hydrogen sulfide (ppb)	1.04 [0.95, 1.14]
Horton et al. 2009	Gloomy blue or unhappy	Hydrogen sulfide (ppb)	1.01 [0.89, 1.15]
Horton et al. 2009	Nervous or anxious	Hydrogen sulfide (ppb)	1.12 [1.03, 1.22]
Horton et al. 2009	Angry grouchy or bad−tempered	PM10 (ug/m3)	1.00 [0.99, 1.01]
Horton et al. 2009	Gloomy blue or unhappy	PM10 (ug/m3)	1.01 [0.99, 1.03]
Horton et al. 2009	Nervous or anxious	PM10 (ug/m3)	1.00 [0.99, 1.01]
Horton et al. 2009	Angry grouchy or bad−tempered	Semivolatile PM10 (ug/m3)	1.03 [0.98, 1.08]
Horton et al. 2009	Gloomy blue or unhappy	Semivolatile PM10 (ug/m3)	1.06 [0.97, 1.16]
Horton et al. 2009	Nervous or anxious	Semivolatile PM10 (ug/m3)	1.10 [1.03, 1.17]
**Subjective Exposures /**	**Subjective Outcomes**		
Horton et al. 2009	Angry grouchy or bad−tempered	Twice daily odor rating (0−8)	1.52 [1.37, 1.70]
Horton et al. 2009	Gloomy blue or unhappy	Twice daily odor rating (0−8)	1.43 [1.25, 1.63]
Horton et al. 2009	Nervous or anxious	Twice daily odor rating (0−8)	1.60 [1.41, 1.81]
**Stress**			
**Objective Exposures /**	**Subjective Outcomes**		
Horton et al. 2009	Stressed or annoyed	Hydrogen sulfide (ppb)	1.18 [1.08, 1.30]
Horton et al. 2009	Stressed or annoyed	PM10 (ug/m3)	1.00 [0.99, 1.01]
Horton et al. 2009	Stressed or annoyed	Semivolatile PM10 (ug/m3)	1.06 [1.00, 1.11]
**Subjective Exposures /**	**Subjective Outcomes**		
Horton et al. 2009	Stressed or annoyed	Twice daily odor rating (0−8)	1.81 [1.63, 2.00]

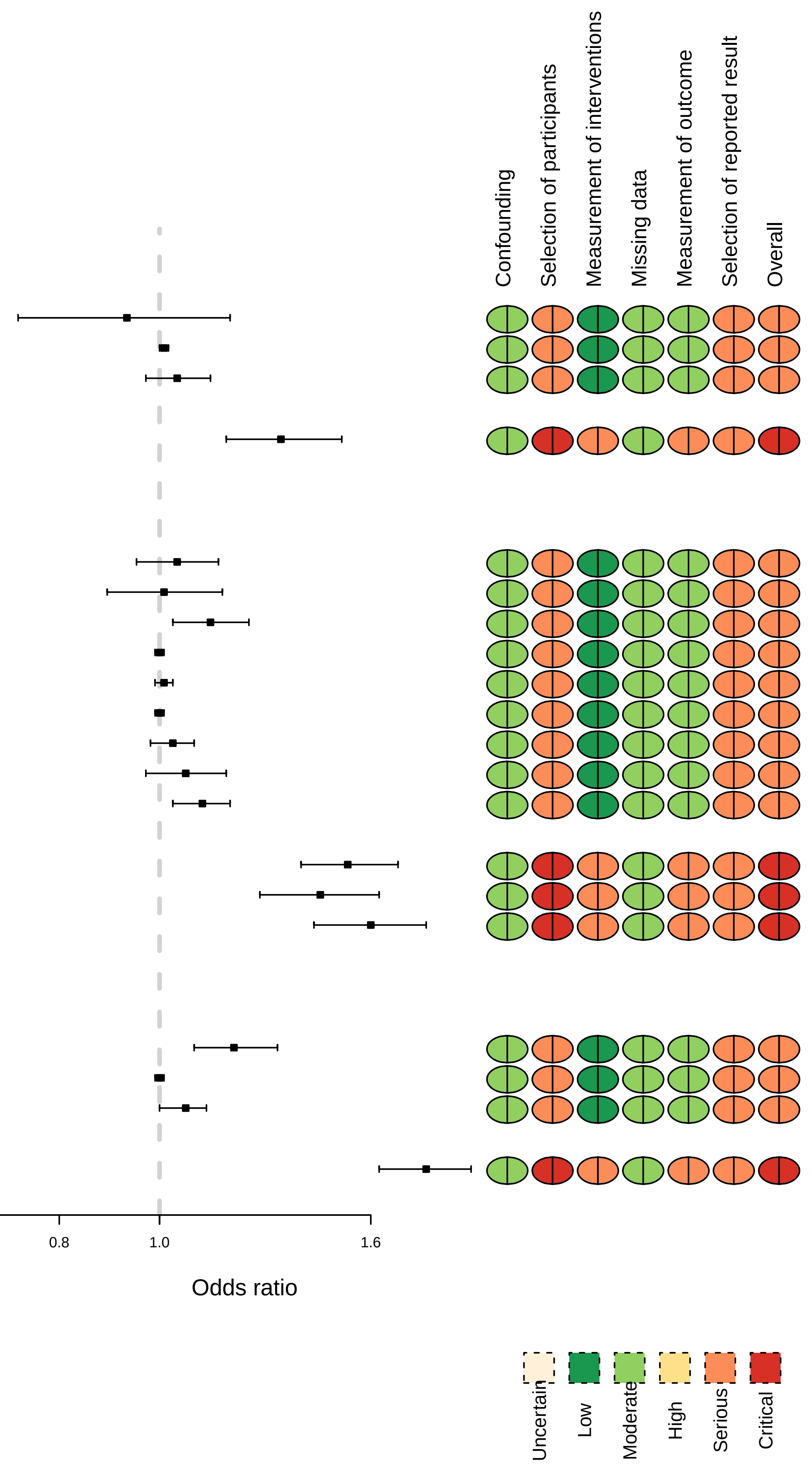

Supplement: Additional file 1: — Discussion about other outcomes included in the systematic review. Excel Spreadsheet with extracted data from review. Data extraction forms, risk of bias forms and search startergies used for review. Figure S1. Neurological and psychological symptoms and stress outcomes for which the effect size was reported as an odds ratio. Figure S2. Neurological symptoms for which the effect size was reported as a regression coefficient. Figure S3. Psychological outcomes for which the effect size was reported as a point estimate of the mean difference. Figure S4. Psychological outcomes for which the effect size was reported as a point estimate. Figure S5. Psychological outcomes for which the effect size was reported as a regression coefficient. Figure S6. Dermatologic, otologic, and optical outcomes for which the effect size was reported as a regression coefficient. Figure S7. Gastrointestinal and “Other” outcomes for which the effect size was reported as a regression coefficient (β). Figure S8. Stress outcomes for which the effect size was reported as a regression coefficient (β). Figure S9. Lower respiratory outcomes for which the effect size was reported as a prevalence ratio. (ZIP 1.40 mb) [file 13643_2017_465_MOESM1_ESM.zip › figS1R1.pdf]
